# A framework for the use of single-chemical transcriptomics data in predicting the hazards associated with complex mixtures of polycyclic aromatic hydrocarbons

**DOI:** 10.1007/s00204-016-1891-8

**Published:** 2016-11-17

**Authors:** Sarah Labib, Andrew Williams, Byron Kuo, Carole L. Yauk, Paul A. White, Sabina Halappanavar

**Affiliations:** Environmental Health Science and Research Bureau, Health Canada, Ottawa, ON K1A 0K9 Canada

**Keywords:** Polycyclic aromatic hydrocarbons, Toxicogenomics, Transcriptomics, Complex mixtures, Additivity

## Abstract

The assumption of additivity applied in the risk assessment of environmental mixtures containing carcinogenic polycyclic aromatic hydrocarbons (PAHs) was investigated using transcriptomics. MutaTMMouse were gavaged for 28 days with three doses of eight individual PAHs, two defined mixtures of PAHs, or coal tar, an environmentally ubiquitous complex mixture of PAHs. Microarrays were used to identify differentially expressed genes (DEGs) in lung tissue collected 3 days post-exposure. Cancer-related pathways perturbed by the individual or mixtures of PAHs were identified, and dose–response modeling of the DEGs was conducted to calculate gene/pathway benchmark doses (BMDs). Individual PAH-induced pathway perturbations (the median gene expression changes for all genes in a pathway relative to controls) and pathway BMDs were applied to models of additivity [i.e., concentration addition (CA), generalized concentration addition (GCA), and independent action (IA)] to generate predicted pathway-specific dose–response curves for each PAH mixture. The predicted and observed pathway dose–response curves were compared to assess the sensitivity of different additivity models. Transcriptomics-based additivity calculation showed that IA accurately predicted the pathway perturbations induced by all mixtures of PAHs. CA did not support the additivity assumption for the defined mixtures; however, GCA improved the CA predictions. Moreover, pathway BMDs derived for coal tar were comparable to BMDs derived from previously published coal tar-induced mouse lung tumor incidence data. These results suggest that in the absence of tumor incidence data, individual chemical-induced transcriptomics changes associated with cancer can be used to investigate the assumption of additivity and to predict the carcinogenic potential of a mixture.

**Electronic supplementary material:**

The online version of this article (doi:10.1007/s00204-016-1891-8) contains supplementary material, which is available to authorized users.

## Introduction

Human exposures to environmental chemicals occur via contact with complex mixtures rather than single chemicals in isolation. Currently, quantitative hazard and risk assessment of chemical mixtures is conducted using two approaches: (a) a whole mixture approach or (b) a component-based approach that only examines prioritized mixture components. The whole mixture approach is used when data are available for the mixture in question or for a surrogate mixture that is sufficiently similar to the mixture under investigation. The whole mixture approach is preferred by several regulatory agencies such as Health Canada and the US Environment Protection Agency (CCME 2010; USEPA 2010); however, due to difficulties in processing mixtures (e.g., atmospheric particulate matter) and the lack of standards for positive controls, the whole mixture approach cannot be used for routine mixture toxicity testing and risk assessment. Similarly, comparing each newly identified mixture to a sufficiently similar mixture is complicated because of the relative differences in mixture composition that may influence the overall toxicity of the mixture. Thus, assuming that the total risk and hazard associated with a mixture is the sum of the contributions from the known priority components, and the component-based approach has been deemed the most appropriate and practical for routine assessments. This approach is also referred to as the additivity approach, and it is currently employed by multiple regulatory agencies worldwide, including Health Canada (2010), the USEPA (2010), and the European Commission (2001).

The component-based approach relies on a series of established reference models centered on the assumption that individual components of the mixture do not interact, do not influence each other’s toxicity, and that their toxicological activity is additive. Accordingly, the toxicity of mixtures composed of similarly acting chemicals (e.g., those with similar biochemical mechanisms of toxicity or carcinogenic modes of action) is calculated as the sum of the concentrations/doses of selected chemicals, each adjusted using a relative potency scalar to convert amounts to equivalents of a potent reference substance (i.e., the concentration addition or CA model). For mixtures of dissimilarly acting chemicals, the total toxic response of the mixture is calculated by summing the toxic responses of each chemical component (i.e., the independent action or IA model, also known as response addition and effect addition models). Although pragmatic, the CA approach is limited in scope since it can only estimate the combinatorial toxicity of the mixture up to the maximal toxic response of the weakest mixture component. Consequently, recent advances in mixtures toxicology have led to the development of alternative models, such as generalized concentration addition model (GCA) (Howard and Webster 2009), which considers the maximal toxic response of each mixture component. The GCA model has been used effectively in predicting toxicological responses of mixtures of aryl hydrocarbon receptor (AHR) agonists (Howard et al. 2010). Together, these models have proven useful for identifying interactive behaviors in defined mixtures (i.e., deviations from additivity), as well as for understanding the influence of unknown substances on the additive behavior of known mixture components (European Commission 2012).

As the toxicology community embarks upon a paradigm shift toward more mechanistic testing strategies (NRC 2007), applications of omics tools, such as transcriptomic studies investigating global changes in gene expression, are providing insight into the toxic action of chemicals and comprehensive information on the biological pathways and processes disturbed by environmental chemicals. However, despite noteworthy advantages, few studies have applied transcriptomics to investigate the joint effects of chemicals in mixtures. The advantages of transcriptomic tools for mixture assessments relate to the high-content data that are generated, and the utility of such data for identifying and discriminating pathways and processes affected by individual mixture components and the mixtures themselves. Such analyses can determine if structurally similar chemicals indeed act via a common mechanism and can provide predictive insight regarding the likelihood of chemical interactions.

Polycyclic aromatic hydrocarbons (PAHs) are a class of structurally similar chemicals, many of which have been characterized as genotoxic carcinogens. They are formed during the incomplete combustion of organic materials and are ubiquitously found in many environmentally relevant complex mixtures to which humans are regularly exposed. Human health risk assessments of PAH mixtures commonly employ the CA approach that incorporates quantitative contributions from the 16 PAHs that were previously selected by the USEPA as priorities for concern and control (i.e., priority PAHs). In a recent study, we selected 8 of the 16 priority PAHs that are genotoxic carcinogens and applied global transcriptomic profiling, in combination with other apical endpoint analyses, to determine if these 8 PAHs share a similar mode of action (Labib et al. 2015). The results revealed that the eight structurally similar PAHs all induced DNA damage, mutation, and enzyme activity; however, at the transcriptional level, the response was markedly different. Indeed, the individual PAHs induced alterations in the expression of genes associated with diverse arrays of biological functions and processes that were not common to all eight compounds. Furthermore, the transcriptional profiles were tissue specific (Labib et al. 2012, 2013, 2015). The results suggested that the underlying genotoxic and carcinogenic mechanisms of action for these eight PAHs are not identical. This led us to hypothesize that the assumption regarding a similar mode of action may be inaccurate.

The primary objective of the present study is to examine the utility of transcriptomic data for investigating the combined effects of chemicals in mixtures. Since we have tissue-specific transcriptomic data for the eight individual PAHs, this study examined PAH-containing mixtures. More specifically, whole genome transcriptional profiling was used to examine pathways/processes perturbed by exposure to two defined mixtures of four and eight PAHs, and a coal tar extract (a highly complex mixture of PAHs). Since the lung transcriptome was the most perturbed of the three tissues studied in our previous work in terms of transcriptomic responses and DNA damage induction, in this study the pulmonary transcriptomes following exposure to the mixtures were compared to the pulmonary transcriptomes from our previous studies of eight PAHs individually (Labib et al. 2012, 2013, 2015). Furthermore, the comparisons were used to evaluate the CA, GCA, and IA models, to investigate interactions between priority PAHs and, lastly, to determine the influence of unknown substances on the activity of the complex mixtures via comparison of the simple and complex mixtures. The analyses specifically examined pathways associated with cancer since the PAHs examined are known or suspected carcinogens. The data were further used to assess whether the predicted and actual perturbations of cancer-associated pathways elicited by coal tar exposures are aligned with the lung tumor incidence observed in the Culp et al. murine carcinogenicity study (Culp et al. 1998).

## Methods

### Chemicals

BaP and chrysene (CHR) were purchased from Sigma-Aldrich Canada Ltd. (Canada). Benz(*a*)anthracene (BaA), benzo(*b*)fluoranthene (BbF), benzo(*ghi*)perylene (BghiP), benzo(*k*)fluoranthene (BkF), dibenz(*ah*)anthracene (DBahA), and indeno(123,*cd*)pyrene (IP) were purchased from Cambridge Isotopes Laboratories (USA). All compounds had purity ≥98.8%.

### Preparation of PAH mixtures and animal exposures

Coal tar extract (CT-Mix) was prepared as described previously (Wise et al. 1988; Long et al. 2016). The analytical assessment of the CT-Mix for volatile organic compounds and PAH content was conducted by Paracel Laboratories Ltd. (Ottawa, ON, Canada), a commercial laboratory accredited according to ISO/IEC 17025:2005 by the Canadian Association for Laboratory Accreditation, by gas chromatography mass spectrometry (EPA reference methods 624 and 625) (further details can be found in Online Resource 1). The relative content of the eight priority PAHs in the CT-Mix was 23983 mg PAH/kg CT-Mix. The relative concentration of each of these eight PAHs relative to total PAH content in the CT-Mix used in this study was within 0–1% of that found in the coal tar sample used in a previously published 2-year feeding study with coal tar (Culp et al. 1998) (Online Resource 1); the content of the eight PAHs in the Culp et al. (1998) study was 12499 mg PAH/kg coal tar.

The proportion of each PAH in the CT-Mix was then used to prepare two defined mixtures containing only four PAHs (4PAH-Mix) or only eight PAHs (8PAH-Mix). The PAHs selected for the 4PAH-Mix and 8PAH-Mix constitute the panel of four and eight PAHs recommended for routine examination of food samples by the European Food Safety Authority because they are the genotoxic PAHs that were measured in coal tar mixtures used in oral carcinogenicity studies (EFSA 2008). Exposure doses for the defined mixtures and the CT-Mix were selected based on the maximum tolerated dose of each mixture determined in a dose range-finding study. This stock was diluted twice, by twofold each, to obtain two lower doses. The specific doses in the 4PAH-Mix are as follows: BaP (9.9, 19.9, 39.7 mg/kg-day), BaA (12, 23.9, 47.8 mg/kg-day), BbF (12.6, 25.3, 50.6 mg/kg-day), and CHR (11.4, 22.7, 45.5 mg/kg-day). This provided doses of 12.5, 25, and 50 mg BaP equivalents/kg-day based on the CCME PAH-specific potency equivalence factors (CCME 2010). The specific doses of the 8PAH-Mix are as follows: BaP (10.5, 20.9, 41.9 mg/kg-day), BaA (12.6, 25.2, 50.4 mg/kg-day), BbF (13.3, 26.7, 53.4 mg/kg-day), CHR (12.0, 24.0, 48.0 mg/kg-day), BghiP (5.7, 11.4, 22.7 mg/kg-day), BkF (6.9, 13.7, 27.5 mg/kg-day), DBahA (1.2, 2.4, 4.8 mg/kg-day), and IP (5.2, 10.4, 20.9 mg/kg-day). This provided doses of 15, 30, and 60 mg BaP equivalents/kg-day. The CT-Mix was diluted to provide doses of 1.3, 2.5, and 5.1 mg BaP equivalents/kg-day.

The animal care, exposures, and tissue collection procedures have been previously described (Labib et al. 2012; Lemieux et al. 2011). Mice were bred, maintained, and treated in accordance with the Canadian Council for Animal Care Guidelines, and all protocols were approved by Health Canada’s Animal Care Committee. In brief, adult, male Muta™Mouse (transgenic mouse strain 40.6 on BALB/C-DBA/2 background) were exposed by oral gavage for 28 consecutive days to olive oil (vehicle control) or to three doses of each of the PAH-containing mixtures. The dosing regime follows the established Organisation for Economic Co-operation and Development (OECD) guideline for transgenic rodent mutation assays (i.e., TG #488) (OECD 2011). Each treatment group contained five animals. Three days after the final dose animals were sacrificed by cardiac puncture under isoflurane anesthesia. Two mice from the 8PAH-Mix died as a result of accidental pulmonary puncture during oral gavage. Thus, the final sample size for the 8PAH-Mix experiment was 5, 5, 4, and 4 in the control, low, medium, and high-dose groups, respectively. The right lobe of the lung was collected, flash-frozen in liquid nitrogen, and stored at −80 °C until use.

### Tissue RNA extraction and purification

Total RNA for gene expression analysis was isolated from the lung using TRIzol reagent (Invitrogen, Canada) and purified using RNeasy Mini Kit (Qiagen, Canada) as described previously (Halappanavar et al. 2011). All samples met quality control standards for RNA with A260/A280 ratios between 2.0 and 2.2 determined using a NanoDrop Spectrophotometer (Thermo Fisher Scientific, Canada) and RNA integrity numbers above 8.0 determined using an Agilent 2100 Bioanalyzer (Agilent Technologies Inc., Canada).

### Microarray hybridization and analysis

A detailed description of the microarray hybridization and statistical analysis protocols has been published previously (Labib et al. 2013). With the exception of the 8PAH-Mix (see above), 5 replicates were examined for each PAH mixture treatment group. Briefly, 200 ng of total RNA from each individual sample and 200 ng of Universal Mouse Reference RNA (Stratagene, Canada) were used to synthesize cDNA and cyanine-labeled cRNA using the Agilent Linear Amplification Kit (Agilent Technologies Inc., Canada). The labeled cRNAs (i.e., Cyanine-5 for experimental samples and Cyanine-3 for reference RNA) were purified using RNeasy Mini Kits (Qiagen, Canada). 300 ng of labeled cRNA from each experimental sample was hybridized with the same amount of labeled reference RNA to Agilent Sureprint G3 Mouse GE 8 × 60 K microarrays (Agilent Technologies Inc., Canada) at 65 °C for 17 h in the Agilent SureHyb hybridization chamber. The arrays were washed and scanned on an Agilent G2505B Scanner according to the manufacturer’s recommendations. Data were extracted using Feature Extraction 10.7.3.1 (Agilent Technologies Inc., Canada).

Details of the statistical normalization methods for the transcriptomic data were published previously (Labib et al. 2013). Briefly, a reference design (Kerr and Churchill 2001, 2007) was used to analyze microarray data. Non-background median signal intensities were normalized using LOWESS (Yang et al. 2002) using the R (R Development Core Team 2010) platform. A transcript was considered to be a differentially expressed gene (DEG: upregulated or downregulated relative to the vehicle treated controls) using the MAANOVA library in R (Wu et al. 2003). The Fs statistic was used to test for treatment effects (Cui et al. 2005). The P values for all statistical tests for each probe ID were estimated by the permutation method using residual shuffling followed by adjustment for multiple comparisons using the false discovery rate (FDR) approach (Benjamini and Hochberg 1995). The fold change calculations were based on the least-square means (Goodnight and Harvey 1978; Searle et al. 1980). All microarray results are available in the Gene Expression Omnibus database (http://www.ncbi.nlm.nih.gov/geo/) under the accession number GSE87691.

Biological replicates of each experimental condition were collapsed to an average expression value for each gene and normalized to the median of the control samples. This dataset was then filtered using the DEGs from the three independent MAANOVA analyses. Hierarchical clustering was then applied to the filtered data using the one minus correlation dissimilarity metric using the Spearman correlation with average linkage. Data were visualized using a heatmap.

### Bioinformatics and pathway analysis

The list of DEGs (FDR *P* ≤ 0.05, fold change ±1.5 in at least one dose group) from each PAH congener (Labib et al. 2012, 2015), the two defined PAH mixtures, and the coal tar extract were independently analyzed to identify biological functions or processes perturbed in response to the treatments. DAVID (Huang et al. 2009) Functional Annotation Charts were used to identify gene ontology (GO) terms (biological processes and cellular compartments) associated with the significant genes, and to classify the DEGs into biological pathways using KEGG pathways (Kanehisa and Goto 2000). Ingenuity Pathway Analysis (IPA, Ingenuity Systems, Redwood City, CA, USA) Canonical Pathway analysis, Biological Function analysis, and Network analysis were used to identify biological pathways and functions associated with the DEGs. Based on the tool used, different criteria were used to determine the significance of pathways, functions or biological processes perturbed. Any pathway or process that was associated with more than 3 DEGs was included in the interpretation of the results. In addition, an EASE score (right-tailed Fisher’s test) cutoff of *P* ≤ 0.05 was applied to DAVID ontologies and KEGG pathways, and *P* ≤ 0.05 to IPA canonical pathways. Redundancy in pathways and processes was reduced by collapsing multiple pathways or processes implying perturbation of the same biological function as described in Labib et al. (2015). Since our previous studies (Labib et al. 2012, 2013, 2015) focussed on carcinogenic effects of PAHs, pathways pertinent to cancer formation were the focus of the analysis in the present study.

### Transcriptional benchmark dose (BMD) analysis

BMDExpress version 1.4.1 (Yang et al. 2007) was used to perform BMD analysis on the transcriptomic data. Only genes that had “present” calls in at least one dose group (i.e., 4 out of 5 biological samples within at least one experimental group with signal intensities above the background non-murine control probes by at least three standard deviations), fold change ≥±1.5, and ANOVA *P* < 0.05 were modeled. Hill, Power, Linear, and Polynomial (2^0^ and 3^0^) models were used to fit the gene expression dose–response data. For each gene, the best fitting model was selected based on (1) a nested Chi-square test (cutoff of 0.05) to choose between linear and polynomial models, (2) the lowest Akaike information criterion (AIC) values for the nested, Hill, and power models, and (3) curve goodness-of-fit *P* > 0.1. Genes with BMD values higher than the highest dose were excluded. The resulting gene BMD datasets were mapped to all pathways/processes described in Labib et al. (2015). Median BMD and BMDL (lower confidence limit) were reported for pathways with at least three DEGs.

### Applications of the component-based reference models to evaluate additivity

**Fig. 1:**
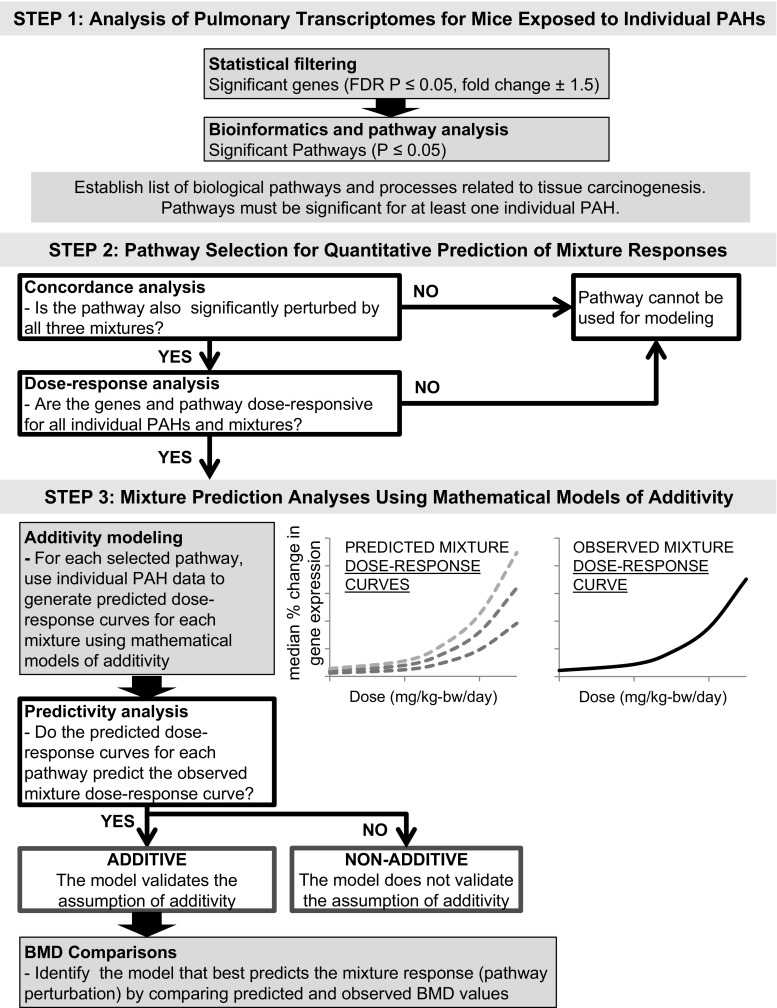
Workflow to select pathways for mathematical modeling and mixture prediction analyses. In step 1: analysis of pulmonary transcriptomes for mice exposed to individual PAHs, following statistical filtering and bioinformatics and pathway analysis, pathways were selected based on cancer-related biological pathways and processes perturbed by the individual PAHs examined in Labib et al. (2015). The number of significant genes and significant pathways induced by each PAH are shown. In step 2: pathway selection for quantitative prediction of mixture responses, pathways were selected following concordance and dose–response analyses (*white boxes*). Pathways that did not pass concordance and dose–response analyses were not used for mathematical modeling. In step 3: mixture prediction analyses using mathematical models of additivity, the individual PAH data were used to generate predicted dose–response curves for each mixture using three established mathematical models of additivity—concentration addition (CA), generalized concentration addition (GCA), and independent action (IA). The predicted dose–response curves and the BMDs for the predicted dose–response curves were compared with the observed mixture dose–response curves and BMDs for the observed dose response curves in the predictivity analysis and BMD comparison

The transcriptomic data for individual PAHs (including the degrees of pathway perturbations induced by each PAH and transcriptional BMDs for each gene and pathway induced by each PAH) were used to generate predicted dose–response curves for the perturbed pathways for each PAH-containing mixture using three models of additivity (CA, GCA, and IA). Thus, when we refer to predicted pathway perturbation or predicted dose–response curves, we are referring to those generated using the CA, GCA, and IA models. The complete strategy is described in detail in Fig. 1.

*Step 1: Analysis of pulmonary transcriptional data for mice exposed to individual PAHs*. In the first step, lung-specific transcriptional changes for eight individual PAHs were analyzed at each dose to identify significantly altered pathways associated with pulmonary carcinogenesis (analyses are described previously by Labib et al. (2012, 2013, 2015)); of these pathways, eight were identified as specifically important for PAH-induced carcinogenesis, and these were considered for mathematical modeling as described below.

*Step 2: Pathway selection for quantitative prediction of mixture responses.* For the selection of pathways to be used in the additivity modeling, we first assessed if these eight pathways were also significantly perturbed following exposure to the three PAH-containing mixtures (concordance analysis). Since alteration in expression of individual genes can be associated with several redundant biological functions and processes, the ability of a PAH or a PAH mixture to perturb biological pathways (reflective of collective expression changes in several genes) was selected as the metric for prediction calculations (Fig. 1). More specifically, the median percent change in gene expression for genes annotated to a pathway (that passed the BMDExpress filter), relative to controls, was used as the effect level to represent pathway perturbations for comparisons of predicted and observed responses. For example, a 50% change in gene expression corresponds to an absolute 1.5-fold change. The maximal effect level was defined as the largest median percent gene expression change for a particular pathway at any of the doses tested (this value was necessary for the GCA model described below). BMD modeling was used to conduct pathway dose–response assessment as described above, and pathway median BMD values were used in lieu of ED50 values that are traditionally used in predictive models of mixture toxicity.

*Step 3: Mixture prediction analyses using mathematical models of additivity.* For each selected pathway, CA (also known as dose addition), GCA, and IA models were then employed to predict pathway perturbations associated with mixture exposures. The formulas and methodologies employed for CA, GCA, and IA modeling are described below.

1. The CA model is used to predict the concentrations of the mixture that would elicit a predetermined effect. In principle, concentrations of individual mixture components that are capable of eliciting the same effect on their own are added, after being multiplied by a scaling factor that accounts for potency differences. CA predictions were modeled according to Eq. 1.
  1 $$D_{{{\text{pred}} . {\text{mix}}}} = \left( {\sum \frac{{p_{\text{PAH}} }}{{d_{\text{PAH}}^{\text{e}} *{\text{PEF}}_{\text{PAH}} }}} \right)^{ - 1}$$
  where $D_{{{\text{pred}} . {\text{mix}}}}$ is the predicted dose of the mixture across a range of effect levels, which were determined starting with the maximal effect level of the weakest inducer for that pathway and declining down to zero (the effect levels correspond to the benchmark response (BMR) used to generate BMDs). $d_{PAH}^{e}$ is the dose level (BMD) at which each PAH on its own exerts the effect level (BMR); ${\text{PEF}}_{\text{PAH}}$ is the potency equivalence factor (PEF) for each PAH scaled for potency relative to BaP (CCME 2010) (BaP = 1; BaA = 0.1; BbF = 0.1; Chr = 0.01; BghiP = 0.01; BkF = 0.1; DBahA = 1; and IP = 0.1); and $p_{\text{PAH}}$ is the fraction of each PAH in the mixture. $D_{{{\text{pred}} . {\text{mix}}}}$ values were calculated for a range of effect levels (up to the maximal effect level of the weakest inducer of that pathway) and a prediction dose–response curve was established. In the case of PAHs for which selected pathway BMDs could not be calculated (due to a lack of genes that passed the BMDExpress filters described above) mathematical modeling proceeded without data input for that PAH. Statistical uncertainties for the predicted effects were presented as the most conservative estimate of the upper and lower bounds for each mixture component using two-sided confidence intervals for the BMD.
2. The GCA model is an extension of the CA model that accounts for the maximal effect level of the mixture components by including a parameter ($e_{\text{PAH}}^{ \hbox{max} }$) for the maximum effects induced by each mixture component. GCA predictions were modeled according to Eq. 2.
  2 $$E_{{{\text{pred}} . {\text{mix}}}} = \frac{{\sum \left( {{\raise0.7ex\hbox{${{\text{e}}_{\text{PAH}}^{ \hbox{max} } *\left[ {\text{PAH}} \right]}$} \!\mathord{\left/ {\vphantom {{{\text{e}}_{\text{PAH}}^{ \hbox{max} } *\left[ {\text{PAH}} \right]} {d_{\text{PAH}} }}}\right.\kern-0pt} \!\lower0.7ex\hbox{${d_{\text{PAH}} }$}}} \right)}}{{1 + \sum \left( {{\raise0.7ex\hbox{${\left[ {\text{PAH}} \right]}$} \!\mathord{\left/ {\vphantom {{\left[ {\text{PAH}} \right]} {d_{\text{PAH}} }}}\right.\kern-0pt} \!\lower0.7ex\hbox{${d_{\text{PAH}} }$}}} \right)}}$$
  where $E_{{{\text{pred}} . {\text{mix}}}}$ is the predicted pathway perturbation (as above, the percent change in gene expression for responding genes in a pathway) induced by the mixture at a specific dose; ${\text{e}}_{\text{PAH}}^{ \hbox{max} }$ is the maximal effect level, which is the maximum pathway perturbation (median percent change in gene expression) induced by each PAH mixture component; $\left[ {\text{PAH}} \right]$ is the concentration of the individual PAH in the mixture at a specific mixture concentration; $d_{\text{PAH}}$ is the dose level (BMD) at which each PAH exerts a 50% change in gene expression, which corresponds to an absolute 1.5-fold change. $E_{{{\text{pred}} . {\text{mix}}}}$ values were calculated for the mixture concentrations and a prediction dose–response curve was established. In the case of PAHs for which selected pathway BMDs could not be calculated (due to a lack of genes that passed the BMDExpress filters described above) mathematical modeling proceeded without data input for that PAH. Statistical uncertainties for the predicted effects were expressed as the most conservative estimate of the upper and lower bounds for each mixture component using two-sided confidence intervals for the BMDs.
3. The IA model is commonly used for prediction of the mixture toxicity when the mixture under consideration contains compounds with diverse modes of action. IA predictions were modeled according to Eq. 3.
  3 $$E_{{{\text{pred}} . {\text{mix}}}} = 1 - \prod \left( {1 - e_{\text{PAH}} *p_{\text{PAH}} } \right)$$
  where $E_{{{\text{pred}} . {\text{mix}}}}$ is the predicted pathway perturbation (as above, the percent change in gene expression for responding genes in a pathway) induced by the mixture at a specific mixture concentration; $e_{\text{PAH}}$ is the median percent change in gene expression for responding genes in a pathway for each PAH at that concentration; and $p_{\text{PAH}}$ is the fraction of each PAH in the mixture. For a range of mixture concentrations, $E_{{{\text{pred}} . {\text{mix}}}}$ values were calculated and a prediction curve was established. Statistical uncertainties for the predicted effects were expressed as 95% confidence bands.

#### Predictivity analysis

For each of the pre-selected pathways, the predicted dose–response curves were compared to the actual, observed mixture dose–response curves (Fig. 1). If the confidence bands around the predicted dose–response curve generated using each model overlapped with the 95% confidence band around the observed dose–response curve, then the assumption of additivity under that specific model was considered validated. If the confidence bands did not overlap, the model was considered invalid with respect to the assumption of additivity, which suggests chemical interactions.

#### BMD comparisons

As a quantitative measure of similarity or dissimilarity between dose–response curves, BMD and BMDL values calculated for the predicted dose–response curves generated by each of the models were compared with the observed BMD values. BMD values were calculated using the USEPA’s Benchmark Dose Software BMDS version 2.5.1 (http://www.epa.gov/ncea/bmds/) (Davis et al. 2011). Data points (doses and effect levels) across the predicted dose–response curves generated using the CA, GCA, and IA models were selected from the mathematical modeling output for BMD modeling. These data were modeled as continuous data and were run against Exponential, Hill, Power, Polynomial, and Linear models of dose–response. The BMR was set to 50% corresponding to a 1.5-fold change in gene expression, which is thought to represent a biologically meaningful critical effect level for gene expression changes. The best model for each dataset was selected based on the lowest AIC value, excluding models with a goodness-of-fit *P* < 0.1.

### BMD analysis of lung tumor incidence data

The transcriptomic data were further used to assess whether the predicted and actual perturbations of cancer-associated pathways following exposures to CT-Mix are reflective of the actual lung tumor incidence observed in mice exposed to coal tar. The lung tumor incidence data from a published 2-year feeding study in mice (Culp et al. 1998) were used. In this study, coal tar extracts were added to the feed of 5-week old female B6C3F1 (C57BL/6 N/X/C3H/HeNMTV) mice at 0, 0.01, 0.03, 0.1, 0.3, 0.6, and 1.0% per day, or 0, 0.22, 0.66, 2.2, 6.6, 13.4, 22.0 mg BaP equivalents/kg-bw/day (Benford et al. 2010). Tumors formed in the tongue, esophagus, forestomach, liver, and lung tissues. The lung tumor incidence numbers as reported by Culp et al. (1998) were used to model the cancer dose–response using BMDS. Since the tumor incidence plateaus in the highest dose groups (13.4 and 22.0 mg BaP equivalents/kg-bw/day), those dose groups were removed from the BMD analysis to improve model fit. The data were modeled as dichotomous tumor incidence and were run against all degrees of the multistage model. The BMR was set to 10% extra risk as recommended by the Benchmark Dose Technical Guidance document (USEPA 2012). The best model for each dataset was selected based on the lowest AIC value, excluding models with a BMD higher than the highest dose, a BMD/BMDL ratio greater than 5.0, and a goodness-of-fit *P* < 0.1.

## Results

In three separate studies, adult male Muta™Mouse specimens were administered three doses of three PAH-containing mixtures (i.e., 4PAH-Mix, 8PAH-Mix, and CT-Mix) for 28 consecutive days by gavage. For the defined mixtures, the PAHs were mixed according to their proportional amounts in the complex coal tar extract. The exposure regime did not cause any overt signs of toxicity and there was no significant body weight loss in any of the exposed mice compared to vehicle treated controls. Lung tissue was collected three days after the final exposure, and global changes in the lung transcriptome were assessed. PAH mixture-specific transcriptomic profiles were established, and the results were quantitatively compared with transcriptomic profiles from pulmonary tissue of mice similarly exposed to eight individual PAH congeners that are constituents of the mixtures examined herein (Labib et al. 2012, 2015). The comparisons of the transcriptomes were used to investigate whether individual mixture components (i.e., individual PAHs) induce transcriptomic responses that are similar to those induced by PAH mixtures, and whether pathway perturbations induced by individual PAHs and an assumption of additivity can be used to predict the extent of pathway perturbations elicited by the examined mixtures.

### General overview of mixture microarray results

**Fig. 2:**
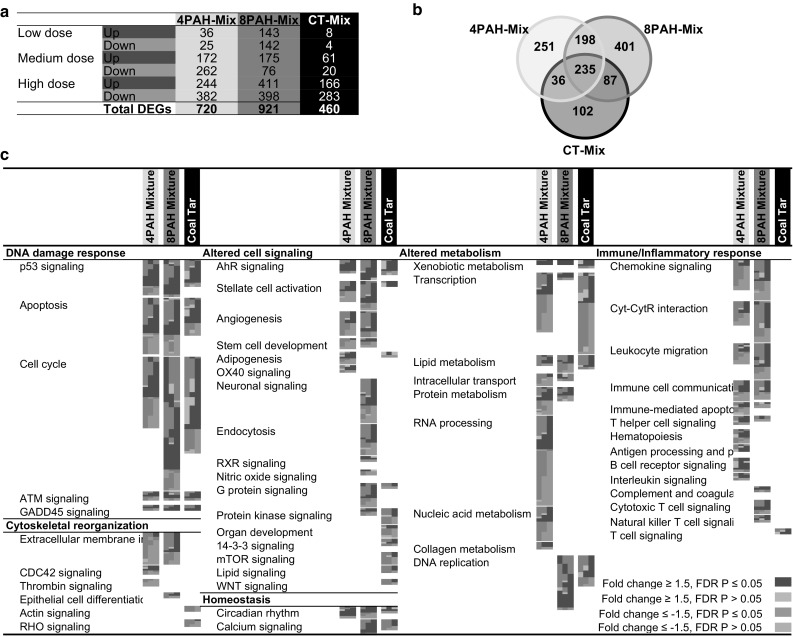
**a** Table of pulmonary DEGs for each dose group of each PAH-containing mixture. **b** VENN diagram showing overlap between DEGs in at least one dose group of the three PAH-containing mixtures. **c** All pathways significantly enriched by each of the PAH-containing mixtures, and their commonalities. *Each column* represents a dose group, and each row represents a gene. All *red* and *green* colored cells represent genes with fold change ≥1.5 in either direction (color figure online)

The 4PAH-Mix, 8PAH-Mix, and CT-Mix induced significant changes in the expression of many genes in lung tissue. MAANOVA analysis revealed 720, 921, and 460 unique DEGs in at least one dose group following exposures to the 4PAH-Mix, 8PAH-Mix, and CT-Mix, respectively (Fig. 2a). Online Resource 2 provides details of all DEGs, including gene names, gene accession numbers, P values, and fold changes. Hierarchical cluster analysis on all DEGs revealed that each PAH mixture group clustered separately from the other (Online Resource 3A), thus a distinct treatment effect was observed as a result of exposure to each of the mixtures. A VENN analysis of the DEGs from all treatment groups revealed 235 DEGs common between the 4PAH-Mix, 8PAH-Mix, and CT-Mix exposures (Fig. 2b).

Figure 2c summarizes all of the molecular pathways and biological processes that are altered in lung tissue following exposure to each PAH mixture. This analysis includes all significantly altered gene ontologies (EASE *P* ≤ 0.05), KEGG pathways (EASE *P* ≤ 0.05), and IPA canonical pathways (*P* ≤ 0.05), as described in the Methods. Six main functional categories of pathways and processes were perturbed: DNA damage response, altered cell signaling, altered metabolism, immune/inflammatory response, cytoskeletal reorganization, and cellular homeostasis.

Clustering analysis of DEGs induced by the mixtures and individual PAH exposures (Labib et al. 2012, 2015) revealed that each PAH mixture clustered separately from the individual PAHs (Online Resource 3B), indicating that the mixture effect is distinct from that elicited by the individual PAHs and that the mixtures are more similar to each other than any other individual PAHs. Furthermore, the three mixtures clustered on the same branch as DBahA and BbF, indicating that there is greater similarity between these two PAHs and the mixtures compared to the other PAHs tested.

### Prediction of mixture pathway perturbations

*Step 1: Transcriptomic profiles of individual PAHs.* The pulmonary transcriptome induced following exposures to eight individual PAHs contained in the 4PAH-Mix and 8PAH-Mix, including details of perturbed pathways and processes, are published previously by Labib et al. (2012, 2013, 2015). Of the many pathways enriched by the individual PAHs, eight biological pathways and processes, which were significantly enriched in at least one individual PAH-treated group, were identified as being relevant to PAH-induced carcinogenesis (i.e., AHR Signaling, Angiogenesis Signaling, Apoptosis Signaling, B Cell Receptor Signaling, Cell Cycle Signaling, Circadian Rhythm Signaling, P53 Signaling, and Xenobiotic Metabolism Signaling pathways). These pathways can be grouped under five main functional categories: DNA damage response, altered cell signaling, altered metabolism, immune/inflammatory response, and cellular homeostasis, all of which play an important role in initiation and/or promotion of cancer. These pathways are employed for the quantitative analyses in steps 2 and 3 below.

**Fig. 3:**
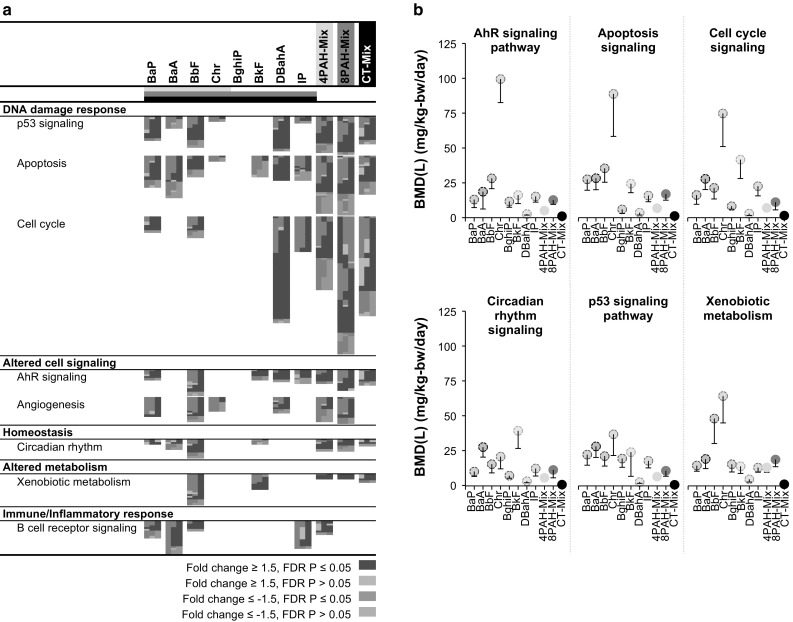
Comparison of cancer-related pathways for individual PAHs and PAH-containing mixtures. **a** Selected cancer-related pathways significantly enriched by each of the individual PAHs and PAH-containing mixtures, and their commonalities. *Each column* represents a dose group and each row represents a gene. All *red* and *green* colored cells represent genes with fold changes ≥1.5 in either direction. **b** BMD10 values in mg/kg-bw/day for each PAH and PAH-containing mixture derived using BMDExpress. The *circles* represent the BMD, and the *lower bars* represent the BMDL (color figure online)

*Step 2: Pathway selection for quantitative prediction of mixture responses.* Pathways associated with cancer formation that were identified in our earlier PAH studies (Labib et al. 2012, 2015) were scrutinized to determine if they were also perturbed following exposures to the 4PAH-Mix, the 8PAH-Mix, and the CT-Mix. Each of the eight cancer-related pathways were perturbed by at least one PAH-containing mixture, with both similarities and differences noted in terms of the identity of the pathways perturbed, and the number of genes perturbed for each pathway (Fig. 3a). Of the eight pathways, Angiogenesis Signaling was significantly affected only by the 4PAH-Mix and the 8PAH-Mix, B Cell Receptor Signaling was affected only by the 4PAH-Mix, and P53 Signaling, Apoptosis Signaling, Cell Cycle Signaling, AHR Signaling, Circadian Rhythm Signaling, and Xenobiotic Metabolism Signaling were significantly affected by all three PAH mixtures. Thus, the six latter pathways, which were perturbed by all three types of mixtures, were selected for further analysis.

Following selection of the relevant pathways, the median percent change in gene expression relative to controls for genes associated with a pathway and the maximal effect levels (i.e., largest median percent change in gene expression relative to controls for genes associated with a pathway across all tested doses) for each pathway were derived for each treatment. Online Resource 4 presents the median and mean gene expression changes for each PAH for all doses and pathways, as well as the fold change of the most affected gene for each pathway. Differences in maximal effects at the doses tested ranged from 48% for CHR and BghiP to 108% for BbF for AHR Signaling, from 56% for BkF to 71% for IP for Apoptosis Signaling, from 59% for BghiP, BkF, and CHR to 75% for BaP for Cell Cycle Signaling, from 58% for DBahA to 167% for IP for Circadian Rhythm Signaling, from 62% for BaA to 174% for CHR for the P53 Signaling, and from 48% for BaA to 106% for IP for Xenobiotic Metabolism Signaling.

Curve fitting and BMD analysis for both probes and pathways altered following exposure to individual PAHs was conducted using BMDExpress. BMD analysis was conducted for a range of BMRs based on the maximum effect levels for the individual PAHs, and median pathway BMD values were calculated for each BMR. Median BMD values at BMR 10% are shown in Fig. 3b. The median BMD values for AHR Signaling ranged from 2.77 mg/kg-bw/day for DBahA to 106.6 mg/kg-bw/day for CHR; Apoptosis Signaling from 3.83 mg/kg-bw/day for DBahA to 94.71 mg/kg-bw/day for CHR; Cell Cycle Signaling from 3.11 mg/kg-bw/day for DBahA to 79.74 mg/kg-bw/day for CHR; Circadian Rhythm Signaling from 3.61 mg/kg-bw/day for DBahA to 42.15 mg/kg-bw/day for BkF; P53 Signaling from 2.92 mg/kg-bw/day for DBahA to 39.51 mg/kg-bw/day for CHR; and Xenobiotic Metabolism Signaling from 5.02 mg/kg-bw/day for DBahA to 68.73 mg/kg-bw/day for CHR.

*Step 3: Mixture prediction using mathematical models of additivity.* In order to determine if pathway perturbations elicited by exposures to individual PAHs, and an assumption of additivity, can be used to realistically predict pathway perturbations elicited by PAH mixtures, we compared predicted mixture pathway perturbations obtained using CA, GCA, and IA component-based models to pathway perturbations induced by the three tested mixtures. Next, the best predictive model was identified by comparing the BMD values for the predicted and observed dose–response functions. The model (CA, GCA, or IA) that yielded a predicted BMD that is closest to the observed BMD was denoted the best model. The results obtained using each of the component-based models are outlined below.

#### Predictivity analysis

**Fig. 4:**
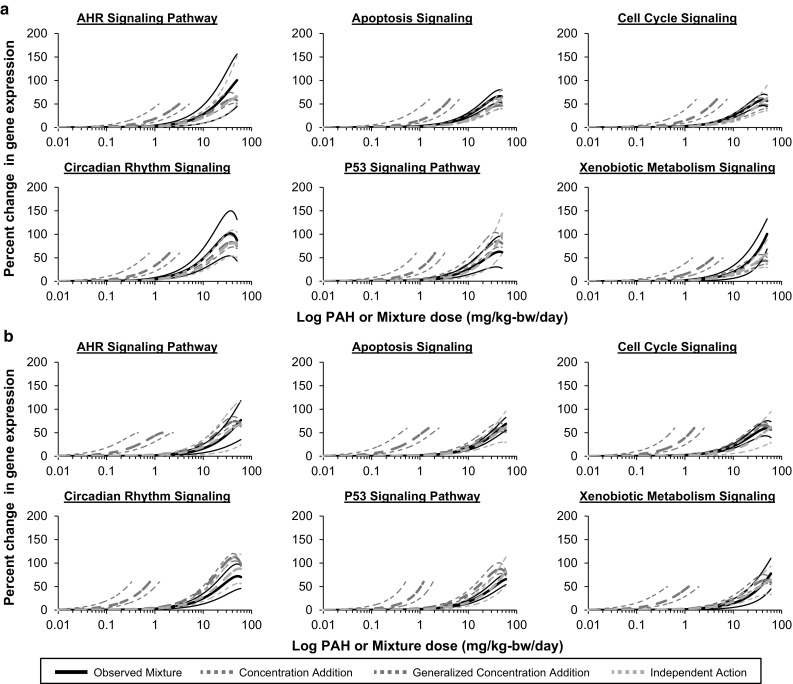
Comparisons of predicted and observed mixture effects for **a** a mixture of four PAHs and **b** a mixture of eight PAHs. Using individual, single-PAH dose–response relationships, additive effects were predicted using the models of concentration addition (CA; *blue dotted lines*), generalized concentration addition (GCA; *purple dotted lines*), and independent action (IA; *orange dotted lines*). Observed dose–response curves are represented by *solid black lines*. *Thin lines* represent 95% confidence bands (color figure online)

**Fig. 5:**
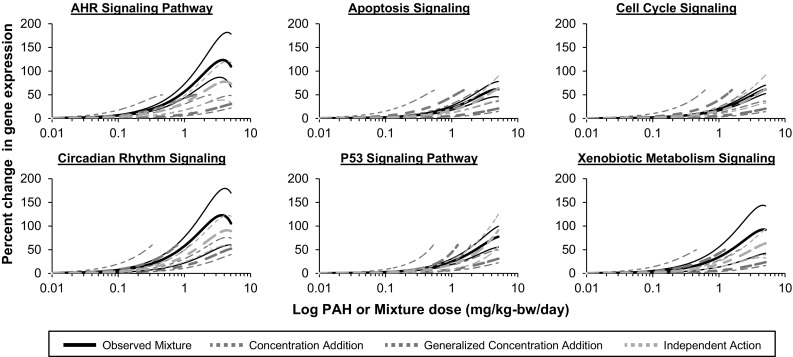
Comparisons of predicted and observed effects of a PAH-containing coal tar extract. Based on the individual single-PAH dose–response relationships, additive effects were predicted using the models of concentration addition (CA; *blue dotted lines*), generalized concentration addition (GCA; *purple dotted lines*), and independent action (IA; *orange dotted lines*). Observed dose–response curves are represented by *solid black lines*. *Thin lines* represent 95% confidence bands (color figure online)

As expected, the CA model (Eq. 1) predicted dose–responses for pathway perturbations up to the maximal effect induced by the least responding individual PAH in the mixture (i.e., weak inducer of gene expression changes). For the 4PAH and 8PAH simple mixtures (Fig. 4a, b), the CA model-predicted dose–response curves are positioned to the left (at lower doses) of the observed dose–response curve for all six pathways tested, and the confidence bands for the predicted and observed pathway perturbations do not overlap. The CA predicted dose–response curves for the CT-Mix were also shifted to the left of the observed pathway perturbations induced by the CT-Mix (Fig. 5); however, the CA model predictions correspond with the observed pathway perturbations of the AHR Signaling, Circadian Rhythm Signaling, P53 Signaling, and Xenobiotic Metabolism Signaling pathways. Additionally, there is a tendency for additivity for the Apoptosis Signaling and Cell Cycle Signaling pathways as the confidence bands between the predicted and observed responses are approaching each other in the low dose regions.

Next we used the GCA model (Eq. 2) to predict mixture-induced pathway perturbations (Figs. 4, 5). This model yielded curves across the full range of doses for each mixture. For the 4PAH-Mix, the GCA model prediction corresponds with the observed pathway perturbations for all six pathways tested (Fig. 4a). For the 8PAH-Mix, the GCA model prediction corresponds with the observed pathway perturbations for AHR Signaling, Apoptosis Signaling, Cell Cycle Signaling, and Xenobiotic Metabolism Signaling pathways (Fig. 4b). For the CT-Mix, GCA model predictions correspond with the observed perturbations for the Circadian Rhythm Signaling pathway and a tendency for additivity was observed for the P53 Signaling and Xenobiotic Metabolism Signaling pathways as the confidence bands between the predicted and observed responses are approaching each other (Fig. 5).

Finally, we predicted the mixture-induced pathway perturbations using the IA model (Eq. 3) generally used for chemicals with dissimilar modes of action. The IA model provided reasonable predictions that corresponded to observed pathway perturbations for all six pathways (Figs. 4, 5) for the 4PAH-Mix, 8PAH-Mix, and CT-Mix.

Please note that in order to assess the impact of filtering the transcriptome data by P value and fold change Steps 2 and 3 were repeated without the application of any filters. More specifically, as in the analysis described above, pathway perturbations were measured as the change in gene expression for all genes annotated to a pathway that passed the BMDExpress filter relative to controls; however, only genes that had a “present” call in at least one dose group were modeled. The results of this analysis were comparable with the results detailed above (Online Resource 5). Briefly, the CA model-predicted dose–response curves were positioned to the left of the observed dose–response curves for all pathways and mixtures tested, the GCA model predictions corresponded with the observed pathway perturbations for several pathways for the defined mixtures but did not correspond with the observed pathway perturbations for the CT-Mix, and the IA model yielded the most reasonable predictions that corresponded to the observed pathway perturbations for all pathways and mixtures tested.

#### BMD comparisons

For the CA model, the predicted BMD50 values for the 4PAH-Mix ranged from a factor of 2.7- (AHR Signaling) to 9.3-fold (P53 Signaling) less than the observed values. For the 8PAH-Mix, the predicted BMD50 values ranged from a factor of 14.5- (Cell Cycle Signaling) to 30.2-fold (Circadian Rhythm Signaling) less than the observed values. For the CT-Mix, the predicted BMD50 values ranged from a factor of 1.3- (AHR Signaling) to 3.5-fold (Circadian Rhythm Signaling) less than the observed values (Table 1).

**Table 1:** Predicted and observed benchmark dose (BMD50) values for selected pathways perturbed by each mixture

Pathway	Observed (mg/kg-day)		CA (mg/kg-day)		GCA (mg/kg-day)		IA (mg/kg-day)	
	BMD	BMDL	BMD	BMDL	BMD	BMDL	BMD	BMDL
*4PAH*-*Mix*								
AHR signaling	8.30	5.60	3.04	2.30	27.54	13.24	29.04	19.57
Apoptosis	15.84	9.54	3.41	2.76	68.29	14.73	37.13	30.12
Cell cycle	15.07	8.67	3.72	3.01	84.43	26.59	36.17	26.23
Circadian rhythm	14.53	6.93	1.61	1.30	10.71	5.59	16.79	12.58
P53 signaling	16.06	9.94	1.73	1.40	12.47	8.21	24.03	20.80
Xenobiotic metabolism	20.97	12.16	3.63	2.77	66.67	46.38	41.25	28.22
*8PAH*-*Mix*								
AHR signaling	25.71	16.04	1.63	0.85	24.27	15.39	31.05	22.07
Apoptosis	32.33	22.24	1.13	0.73	34.50	15.64	40.84	30.96
Cell cycle	18.63	10.93	1.28	1.04	20.97	1.36	43.96	31.57
Circadian rhythm	19.54	13.77	0.65	0.53	4.78	1.53	20.07	15.57
P53 signaling	23.68	16.09	1.04	0.88	11.03	5.34	34.19	28.97
Xenobiotic metabolism	32.86	15.90	1.13	0.86	26.86	15.25	36.41	24.86
*CT*-*Mix*								
AHR signaling	2.24	1.32	1.71	0.79	7.19	5.03	1.73	1.28
Apoptosis	2.53	1.30	1.14	0.80	10.88	6.14	3.52	2.47
Cell cycle	3.02	1.47	1.29	1.05	11.11	6.19	3.69	2.78
Circadian rhythm	2.23	1.21	0.64	0.52	4.72	3.22	1.70	1.34
P53 signaling	1.61	1.03	1.09	0.92	7.29	5.08	2.71	2.32
Xenobiotic metabolism	1.85	1.09	1.18	0.90	9.67	5.87	3.24	2.24

BMDL is the lower 95% confidence limit

For the GCA model, the predicted BMD50 values for the 4PAH-Mix and 8PAH-Mix were close to the observed BMD50 values. For the 4PAH-Mix, the BMD50 values ranged from a factor of 1.3- (P53 Signaling) to 1.4-fold (Circadian Rhythm Signaling) less than the observed values to a factor of 3.2- (Xenobiotic Metabolism Signaling) to 5.6-fold (Cell Cycle Signaling) greater than observed. For the 8PAH-Mix, the BMD50 values ranged from a factor of 1.1- (AHR Signaling) to 4.1-fold (Circadian Rhythm Signaling) less than observed to 1.1-fold (Apoptosis Signaling and Cell Cycle Signaling) greater than the observed values. For the CT-Mix, the predicted BMD50 values ranged from a factor of 2.1- (Circadian Rhythm Signaling) to 5.2-fold (Xenobiotic Metabolism Signaling) greater than observed.

For the IA model, the predicted BMD50 values for the 4PAH-Mix, 8PAH-Mix, and CT-Mix were close to the observed BMD50 values. Specifically, for the 4PAH-Mix BMD50 values ranged from a factor of 1.2- (Circadian Rhythm Signaling) to 3.5-fold (AHR Signaling) greater. For the 8PAH-Mix, the predicted BMD50 values were the same as observed for Circadian Rhythm Signaling and 1.1- (Xenobiotic Metabolism Signaling) to 2.4-fold (Cell Cycle Signaling) greater than the observed values. Similarly, for the CT-Mix, the predicted BMD50 values ranged from a factor of 1.3-fold (AHR Signaling, Circadian Rhythm Signaling) less than observed to 1.2- (Cell Cycle Signaling) to 1.7-fold (P53 Signaling, Xenobiotic Metabolism Signaling) greater than observed.

### Benchmark dose modeling of published cancer data

BMD modeling was conducted using the coal tar-induced lung tumor incidence data from the Culp et al. (1998) study. BMD and BMDL values of 2.47 and 1.50 mg/kg-bw/day, respectively, were determined using the best fit model (i.e., Multistage-Cancer 2°).

## Discussion

In this study, we analyzed the pulmonary transcriptome of mice administered defined- and complex PAH mixtures by gavage daily for 28 days and compared these results with pulmonary transcriptomes of mice similarly exposed to individual PAHs (Labib et al. 2015) to evaluate several component-based models of PAH additivity. We found that (1) PAH-containing mixtures induce expression changes in genes associated with a wide variety of pathways and processes that may be implicated in carcinogenesis, and that (2) with few exceptions, the perturbed pathways were similar to those observed following exposure to individual PAHs. We then used our earlier work on individual PAHs to evaluate the ability of three component-based models of additivity to predict mixture responses at the pathway level. More specifically, we compared pathway perturbations predicted using these models with observed pathway perturbations following exposures to the PAH-containing mixtures. The results were used to assess the validity of the assumptions related to the toxicological additivity of PAHs in simplified and complex PAH mixtures.

Previous studies have used transcriptomic data to evaluate the validity of additivity assumptions and to provide evidence to support toxicological interactions of mixture components (Kopec et al. 2011; Staal et al. 2007; Zucchi et al. 2014). For example, Kopec et al. (2011) applied individual gene expression data to evaluate additivity models using a select set of genes (*Nqo1*, *Dysf*, *Pla2g12a*, *Serpinb6a*, and *Srxn1*) differentially expressed in livers of C57BL/6 mice treated with 2,3,7,8-tetrachlorodibenzo-p-dioxin (TCDD), 2,2′,4,4′,5,5′-hexachlorobiphenyl (PCB153), or a binary mixture of the two. The authors applied statistical modeling of the gene expression data and noted non-additive gene expression responses for several of the individual genes. Similarly, Zucchi et al. (2014) employed individual gene expression values from the ovaries and brains of female zebrafish exposed to drospirenone, progesterone, and a binary mixture of the two to investigate additivity of the substances. The study reported additive and less than additive effects for the three transcripts selected for analysis. In addition to single-gene data, Staal et al. (2007) employed gene expression changes for all DEGs from human hepatoma cells (HEPG2) exposed to binary mixtures of BaP and either DBahA, BbF, fluoranthene, or 1-methylphenanthrene to evaluate an additivity model based on the sum of the gene expression ratios (treated/controls) for individual mixture components. Depending on the genes examined, the authors found additive, antagonistic, and synergistic effects. These studies provide excellent examples of early applications of toxicogenomics in mixtures toxicology, but they fail to address issues related to concentration and effect additivity. Indeed, the concentration-effect additivity dichotomy is germane to mixtures toxicology, and an improved understanding of the toxicological hazards of mixtures must examine both CA and IA approaches, thereby scrutinizing toxicological behaviors of substances present in complex mixtures and the biological processes driving these behaviors.

Although single genes may play important roles in different biological events, they do not reflect the complex interactions and associations that occur among and between genes that trigger biological signaling cascades and cellular pathways. Analysis at the pathway level reduces the complexity of large transcriptomic datasets by relating gene expression changes to mechanistically relevant events. This approach reduces the dimensionality of the data to facilitate comparisons between different exposure scenarios by categorizing individual genes that are related to specific functions. Thus, in the present study we used pathway perturbations to scrutinize assumptions of additivity by evaluating several models of additivity using simple and complex mixtures of PAHs.

As stated earlier, ED50 values are traditionally used in these types of analyses; however, ED50 calculations require knowledge of the maximal effect level of a chemical. Thus, this can pose challenges when comparing chemicals with different capacities to induce a maximal effect. Given these limitations, in place of ED50 modeling we used BMD modeling of the gene and pathway data, which does not require a maximal response and allows the generation of values based on non-asymptotic models (Burgoon and Zacharewski 2008). Thus, BMD analysis using a constant BMR enables comparisons of both strong and weak inducers of a measured effect.

### Pathway perturbations induced by PAHs or PAH-containing mixtures

We found that several key pathways involved in PAH-induced responses, including AHR binding, metabolic activation, DNA damage, and increased cellular proliferation, were all affected at the transcription level by individual PAHs, the 4PAH-Mix, the 8PAH-Mix, and the CT-Mix (Fig. 3a). The results suggest that, in general, the core transcriptomic responses elicited by exposures to PAHs are similar to the responses observed following exposures to the PAH-containing mixtures and supported the original hypothesis that carcinogenic PAHs and PAH mixtures act via mutagenic mode of action. However, some non-mutagenic pathways were also observed to be perturbed. For example, PPAR Signaling, PI3 K/AKT Signaling, PTEN Signaling, IGF-1 Signaling, Glucocorticoid Receptor Signaling, Steroid Biosynthesis, Integrin Signaling, FAK Signaling, and Paxillin Signaling were only perturbed by individual PAHs; whereas, Collagen Metabolism (4PAH-Mix), Leukocyte Migration (4PAH-Mix, 8PAH-Mix), T Cell Signaling (CT-Mix), and mTOR Signaling (CT-Mix) were uniquely perturbed by one or more of the mixtures examined. These pathways have been previously linked to exposures to BaP and other PAH-containing complex mixtures. For example, increased collagen synthesis, which is suggested to play an early role in carcinogenesis, also occurs in lung organ cultures exposed to BaP (Bhatnagar et al. 1980). Leukocyte migration, also known as transepithelial leukocyte migration, was induced by CT-Mix and is altered in human umbilical vein endothelial cells (HUVEC) following exposure to cigarette smoke, a complex mixture containing PAHs (Shen et al. 1996). T cell signaling, which was induced by the CT-Mix, is known to be disrupted by BkF, a PAH that is known to reduce T cell counts (CD4+ and CD8+) in mouse spleen (Jeon et al. 2005). MTOR signaling induced by the CT-Mix has been shown to play an important role in BaP-induced cellular responses (Baumann et al. 2014), as demonstrated by proteomic analysis of BaP-exposed Jurkat T cells. In addition, application of the mTOR inhibitor rapamycin reduced BaP-induced lung tumor formation, growth, and progression in A/J mice (Yan et al. 2006); however, the role of this signaling in BaP-induced tumor formation remains unclear. Thus, in addition to the pathways related to known mutagenic modes of action, perturbation of distinct signaling pathways uniquely induced by individual PAHs, or alternatively, by PAH-containing mixtures, suggests contributions of additional sub-cellular phenomena in PAH-induced carcinogenesis. The perturbation of pathways and mechanisms other than those consistent with the mutagenic mode-of action may explain non-additive behavior of the PAHs observed or predicted by the concentration addition model.

### Predicting pathway perturbations for mixtures of PAHs

In risk assessment, the carcinogenic risks associated with incidental ingestion of materials contaminated with PAHs are typically estimated using the CA approach, which requires an assumption that structurally similar PAHs with similar toxicological outcomes share a common mode of action, and moreover, that their contributions to the toxicological properties of the mixture are additive. The approach employs the concentration of each PAH, and its potency relative to the well characterized reference compound BaP, to determine the contribution of each PAH to the overall toxicity of the mixture. However, the mathematical modeling of pathway perturbations using the CA model described herein, which applied pathway-level gene expression data to the traditional CA model, did not support the assumption that the effective doses of the 4PAH-Mix and the 8PAH-Mix can be determined by the contributions of each PAH in the mixture. This is consistent with previous studies reporting that the CA model can over-estimate the effective concentration (e.g., BaP equivalents) of a PAH mixture relative to the actual toxicological response elicited by the mixture itself. For example, Lemieux et al. (2015) noted that the effective concentration determined by the CA approach yields a level of BaP equivalents that exceed that required to elicit effects observed in cultured cells. In other words, the actual toxicological properties of the mixture are far lower than those predicted by summing the contributions from priority PAHs. Such deviations from additivity emphasize the likely occurrence of antagonistic chemical interactions at the doses employed. Whereas Lemieux et al. (2015) argued that the sub-additive responses of cultured cells to complex PAH mixtures from contaminated soils are indicative of metabolic insufficiency related to competition for the enzymatic machinery required to generate reactive PAH metabolites, and comparison of the predictivity of the CA model for the synthetic mixtures and CT-Mix in this study suggests the potential presence of unknown mixture components that are skewing the estimates for the mixture. As such, PAHs make up only 15% of the entire CT-mix studied and in specific, the 8 PAHs attribute to a small fraction (2%) of the CT-mix. In this case, the CA model tended toward additivity in the degree of pathway perturbations associated with CT-Mix exposures, whereas it over-estimated the degree of pathway perturbations in the synthetic mixtures. The eight PAHs included in this study cover a wide range of genotoxic and carcinogenic potencies, and the published PEFs for these PAHs span two orders of magnitude (USEPA 1993; CCME 2010). In addition, their ability to induce DNA damage and mutations is also variable (Labib et al. 2015; Long et al. 2016). However, previous studies have also shown that other PAHs such as dibenzo[*def,p*]chrysene (Chepelev et al. 2016; Long et al. 2016), which are not regularly measured in PAH-contaminated samples, are more mutagenic than the priority PAHs investigated in the present study. The presence of such PAHs in the CT-Mix may explain the poor predictive power of CA for the CT-Mix, which can result in inaccurate risk estimates for materials contaminated with PAHs.

The GCA model is an extension of the original CA model that takes into consideration the inability of CA model to account for differences in maximal effects between mixture components (Howard and Webster 2009). This approach includes a function that accounts for maximal effect level of each component as well as the Hill slope values applicable to receptor-mediated interactions. Howard et al. (2010) showed that this model was able to accurately predict joint effects of full agonist, partial agonist, and near-competitive antagonist combinations of AHR ligands. In this study, the GCA model yielded improved predictivity compared to the CA model for the synthetic mixtures, but was a poor predictor of the response for the CT-Mix. In the context of the current work, binding and activation of the AHR by PAHs results in transcriptional activation of cytochrome P450 genes 1A1 and 1B1, which encode enzymes that catalyze the formation of dihydrodiol epoxides that react with DNA to form mutagenic bulky adducts. In addition, PAH-related AHR binding induces perturbations in a variety of toxicity and biochemical pathways involved in carcinogenesis (Dietrich and Kaina 2010). Unfortunately, the addition of a metric to represent the maximal effect level may be a limitation of the GCA model in predicting complex mixture responses. The poor predictivity of the GCA model for the CT-Mix may reflect the presence of unknown mixture components with potentially higher maximal effect levels. Thus, despite GCA’s inability to predict the CT-Mix pathway perturbations, for synthetic mixtures it provided improved predictions, relative to the CA approach.

The IA model provides predictions of mixture outcomes by summing the toxic responses of each mixture component. In this study, the IA model provided the best predictions of pathway perturbations for both synthetic and complex PAH mixtures. More specifically, comparison of predicted and observed dose–response curves and BMD values across each of the selected pathways showed the closest correspondence in comparison to what was observed for the CA and GCA models. The IA model assumes that the individual mixture components act via dissimilar modes of action, and this contention is supported by our previous PAH study that provided evidence of distinct compound-specific biochemical mechanisms leading to carcinogenesis (Labib et al. 2015). The combined results from this study and our previous studies suggest that application of CA alone may not be sufficient to predict the effects of the PAHs in mixtures and question the suitability of CA in human health risk assessment of PAH-containing mixtures. Thus, the results of this study support the use of the IA model for PAH-containing mixtures.

It is important to note that although the dose of the coal tar mixture was far below the doses used for the synthetic PAH mixture (in BaP equivalents), at the molecular level they induced similar perturbations of pathways related to carcinogenesis. This suggests that the mechanisms underlying the carcinogenicity of PAH-containing mixtures are concordant. Previously, we showed that the pathways perturbed by the individual PAHs varied across PAHs and tissues (Labib et al. 2015), yet the commonality in the pathways perturbed between the PAH mixtures, as well as between the mixtures and the individual components of the mixtures, suggests that the transcriptomic responses are sensitive enough to identify differences while still being specific enough to tease out commonalities related to carcinogenic transformation.

The identification of which PAH (if any) is the main contributor to a PAH-containing mixture’s carcinogenicity is of particular interest. BaP has traditionally been used as a point of reference in the estimation of excess lifetime cancer risk posed by PAH-containing mixtures (CCME 2010; EFSA 2008; Health Canada 2010). This is primarily based on the fact that BaP’s carcinogenicity and toxicological properties are well characterized and because of the abundance of information related to its occurrence in environmental media. In the present study, hierarchical cluster analysis showed that DBahA and BbF co-clustered on the same branch as the three mixtures, whereas BaP clustered on an adjacent branch. These results suggest that DBahA and BbF may play a strong role in the response induced by the PAH-containing mixtures compared to the other PAHs tested, including BaP, and that perhaps BaP may not be an adequate representative for PAH-containing mixtures. These results are in alignment with the conclusions of our previously published study stating that BaP may not be an appropriate representative of carcinogenic PAHs (Labib et al. 2015). Thus, comparison of the pulmonary transcriptomes from mice exposed to individual PAHs and PAH-containing mixtures showed that DBahA and BbF may have greater influence in PAH-mixture-induced toxicological response.

### Quantitative comparisons of coal tar-induced mouse pulmonary tumors with transcriptomic-based pathway perturbations using predictive models of additivity

This work scrutinized the utility of transcriptomic data for evaluating different models of additivity to assess the toxicological properties of PAHs and PAH mixtures. Since the analyses are based on pathway perturbations related to carcinogenesis, the results have implications for the quantitative risk assessment of PAH-containing mixtures. To enhance the utility of transcriptomic profiling for quantitative risk assessment, we also compared the transcriptional BMD values with those for a relevant apical endpoint (e.g., coal tar-induced lung tumor incidence). The CT-Mix transcriptional BMDs for the cancer-related pathways (Table 1) observed here (ranging from 1.61 to 3.02 mg/kg-day) were similar to the Culp et al. (1998) lung tumor BMD value of 2.47 mg/kg-bw/day. Similarly, the BMDs for the cancer-related pathways generated by the IA model (ranging from 1.70 to 3.69 mg/kg-day) were also close to the lung tumor BMD value. It is important to note that the proportion of non-PAH content in the coal tar used in the Culp et al. (1998) study is not available and thus, it is difficult to accurately conclude if the observed carcinogenic responses in the feeding study are reflected in the transcriptomic responses. Moreover, the background of the B6C3F1 mouse strain used in the Culp et al. (1998) study differs from the background of the Muta™Mouse (BALB/C-DBA/2) used in this study. Despite of the stated differences, there was a high degree of concordance between the transcriptional and lung tumor BMD values. Thus, although based on only one PAH-containing mixture, the concordance between the predicted cancer-pathway BMDs and lung tumor BMDs suggests that this type of cancer-related pathway data predicted using the IA model of additivity involving the eight genotoxic-carcinogenic PAHs might be a useful screening tool for the initial estimation of a PAH-containing mixture’s cancer risk.

## Conclusions

This study used transcriptomics-derived pathway perturbations induced by individual PAHs to evaluate the applicability of such data in deriving predictions of pathway perturbations for synthetic and complex mixtures of PAHs. We demonstrate a strategy that used pathway-level transcriptional data and conventional mathematical models to evaluate several approaches to predict the effects of PAH-containing mixtures. In contrast to the assumption of dose additivity that is commonly employed for PAH-containing mixtures (i.e., CA-based approach), our results imply that perturbations of toxicity pathways, some of which have been implicated in carcinogenesis, are best predicted by a model that assumes dissimilar modes of action for mixture components (i.e., IA-based approach). The median change in expression value for DEGs associated with perturbed pathways and dose–response analyses of the gene expression data for perturbed pathways were successfully incorporated into existing predictive models of additivity. The transcriptional BMDs generated using the mathematical models of additivity resulted in values consistent with lung tumor development induced by coal tar (BMD: 2.5 mg/kg-bw/day; BMDL: 1.5 mg/kg-bw/day). Since the pathway perturbations noted in our study are biologically linked to PAH-induced carcinogenesis, it is biologically plausible that the doses at which these pathways are affected might approximate the dose at which adverse outcome would be expected. As the public repository of genomics datasets for individual chemicals is populated, and the underlying mechanisms of toxicity are revealed, these types of data can be used for prioritizing mixtures for further toxicity testing.

Our comparisons of observed and predicted responses under different additive models indicate that CA, which is traditionally used for risk assessment of PAH mixtures, over-estimates the responses compared to what is actually observed. This suggests that the traditional additive paradigm, which employs CA and PEFs to determine the effective amount of a reference compound (e.g., BaP), may yield conservative risk estimates. Additional work examining other PAH mixtures would be required to validate this conclusion.

## Electronic supplementary material

Below is the link to the electronic supplementary material.

Online Resource 1 Analytical assessment of purified coal tar (PDF 296 kb)

Online Resource 2 Gene lists for PAH-containing mixtures in lung tissues (PDF 6214 kb)

Online Resource 3 Hierarchical cluster analyses (PDF 330 kb)

Online Resource 4 List of median and mean gene expression changes for each pathway in lungs from mice exposed to the 4PAH-Mix, 8PAH-Mix, CT-Mix, and the individual PAHs (PDF 274 kb)

Online Resource 5 Impact of no statistical filtering on mixture predictions using mathematical models of additivity (PDF 185 kb)
