# Supplementary material for: A framework for the use of single-chemical transcriptomics data in predicting the hazards associated with complex mixtures of polycyclic aromatic hydrocarbons
Source: Arch Toxicol. 2016 Nov 17;91(7):2599–616. doi: 10.1007/s00204-016-1891-8 (PMC5489644; doi:10.1007/s00204-016-1891-8)
Supplement: Supplementary file 1 — Analytical assessment of purified coal tar (PDF 296 kb) [file 204_2016_1891_MOESM1_ESM.pdf]

Article Title: A framework for the Use of Single Chemical Transcriptomics Data in Predicting the Hazards Associated with Complex Mixtures of Polycyclic Aromatic Hydrocarbons
Journal: Archives of Toxicology
Authors: Sarah Labib, Andrew Williams, Byron Kuo, Carole L Yauk, Paul A White, Sabina Halappanavar*
Corresponding Author: *Sabina Halappanavar, Health Canada, sabina.halappanavar@hc-sc.gc.ca

Online Resource 1. Analytical assessment of purified coal tar.

	Maximum Detection Limit (mg/L)	Purified Coal Tar in DMSO (mg/L)	Culp et al. Coal Tar Mixture 1 (mg/kg)†
**Volatiles**			
Methylene Chloride	0.005	<25.0	n/a
**Semi-Volatiles**			
Acenaphthene	0.00005	1780	2049
Acenaphthylene	0.00005	11500	3190
Anthracene	0.00001	25000	2524
Benzo[a]anthracene	0.00001	14000	2374
Benzo[a]pyrene	0.00001	12200	1837
Benzo[b]fluoranthene	0.00005	17900	2097
Benzo[g,h,i]perylene	0.00005	7160	1493
Benzo[k]fluoranthene	0.00005	5660	699
Biphenyl	0.00005	4270	n/a
Chrysene	0.00005	12200	2379
Dibenzo[a,h]anthracene	0.00005	1840	267
Fluoranthene	0.00001	84900	4965
Fluorene	0.00005	16100	3692
Indeno[1,2,3-cd]pyrene	0.00005	6890	1353
1-Methylnaphthalene	0.00005	5370	6550
2-Methylnaphthalene	0.00005	10700	11289
Methylnaphthalene (1&2)	0.00001	16100	n/a
Naphthalene	0.00005	76600	22203
Phenanthrene	0.00005	98900	7640
Pyrene	0.00001	72000	5092

n/a - this component was not reported in the Culp et al. (1998) study.
* The coal tar extract in DMSO was sent to Paracel Laboratories Ltd. (Ottawa, ON, Canada) for analytical assessment for volatile organic compounds and PAHs by EPA reference methods 624 and 625. Briefly, the samples were warmed and diluted with dichloromethane and an aliquot was analyzed on an Agilent 6890N gas chromatograph with a split/splitless injector and Restek Rxi-5Sil MS column (30m x 0.25mm ID, film thickness 0.25um, Chromatographic Specialties, Brockville, ON). Target analytes were identified and quantified using an Agilent 5973N mass spectrometer operating in SIM (selected ion monitoring)mode.
† The PAH composition of the Culp et al. (1998) coal tar mixture was assessed by gas chromatography/mass spectrometry.
Note that the relative concentration of each of the eight priority PAHs in the CT-Mix used in this study was comparable to that in the coal tar mixture used by Culp et al. (1998). For example, the proportion of BaP in each coal tar mixture is approximately 2.2% (w/w) and 2.4% (w/v), BaA is 2.9% and 2.8%, BbF is 2.6% and 3.6%, BghiP is 1.8% and 1.4%, BkF is 0.9% and 1.1%, CHR is 2.9% and 2.4%, DBahA is 0.3% and 0.4%, and IP is 1.7% and 1.4% of the Culp et al. (1998) coal tar and the CT-Mix used in the present study, respectively.
