# Supplementary material for: A framework for the use of single-chemical transcriptomics data in predicting the hazards associated with complex mixtures of polycyclic aromatic hydrocarbons
Source: Arch Toxicol. 2016 Nov 17;91(7):2599–616. doi: 10.1007/s00204-016-1891-8 (PMC5489644; doi:10.1007/s00204-016-1891-8)
Supplement: Supplementary file 2 — Gene lists for PAH-containing mixtures in lung tissues (PDF 6214 kb) [file 204_2016_1891_MOESM2_ESM.pdf]

Article Title: A framework for the Use of Single Chemical Transcriptomics Data in Predicting the Hazards Associated with Complex Mixtures of Polycyclic Aromatic Hydrocarbons
Journal: Archives of Toxicology
Authors: Sarah Labib, Andrew Williams, Byron Kuo, Carole L Yauk, Paul A White, Sabina Halappanavar*
Corresponding Author: *Sabina Halappanavar, Health Canada, sabina.halappanavar@hc-sc.gc.ca

Online Resource 2 - Gene lists for PAH mixtures in lung tissues

	Page number
2.1 - 4PAH-Mix	1
2.2 - 8PAH-Mix	19
2.3 - CT-Mix	42

2.1 4PAH-Mix. Significant probe list. List of all significantly differentially expressed probes in at least one treatment group (FDR P ≤ 0.05, fold change ± 1.5) in response to sub-chronic oral exposure to 12.5, 25, and 50 mg BaP equivalents /kg-bw/day of a mixture of 4 PAHs in the lungs. The list is sorted from highest to lowest fold change in the 50 mg BaP equivalents/kg-bw/day treatment group.

			Low dose 12.5 mg BaP equivalents/ kg-day		Medium dose 25 mg BaP equivalents/ kg-day		High dose 50 mg BaP equivalents/ kg-day	
Agilent Probe	Accession Number	Gene Symbol	FDR P value	Fold change	FDR P value	Fold change	FDR P value	Fold change
A_55_P1986282	NM_001111099	Cdkn1a	0	3.4	0	5.9	0	7.6
A_55_P1960735	NM_011819	Gdf15	0	4.1	0	6.9	0	7.4
A_55_P1959500	NM_172759	Ces5	0	4	0	4.8	0	6.2
A_51_P363947	NM_007669	Cdkn1a	0	2.6	0	4.2	0	5.4
A_55_P2032079	NM_016974	Dbp	0	3.7	0	4.9	0	5.4
A_55_P2032081	NM_016974	Dbp	0	3.6	0	4.8	0	5
A_51_P414396	NM_153127	Mmrn2	0.04	1.8	0	2.9	0	3.9
A_55_P1972948	NM_176954	Brunol5	0.11	1.6	0	2.4	0	3.4
A_51_P329928	NM_013750	Phlda3	0	2.1	0	2.8	0	3.4
A_52_P612803	NM_009831	Ccng1	0.02	1.9	0	2.5	0	3.3
A_51_P255456	NM_009994	Cyp1b1	0	1.8	0	2.2	0	3
A_55_P2002577	NM_010145	Ephx1	0	2.2	0	2.8	0	3
A_55_P2031999	NM_145448	9030617O03Rik	0.02	2.1	0	2.5	0	2.9
A_55_P2002578	NM_010145	Ephx1	0	1.9	0	2.2	0	2.8
A_55_P2145804	NM_026531	Aen	0	1.9	0	2.4	0	2.7
A_55_P2005213	NM_145603	Ces2	0	1.8	0	2.5	0	2.7
A_30_P01026536			0	1.6	0	2.1	0	2.6
A_55_P2141860	NM_026531	Aen	0	1.8	0	2.3	0	2.6
A_51_P254425	NM_009644	Ahrr	0	1.6	0	1.9	0	2.6
A_51_P323620	NM_144543	Thyn1	0.05	1.6	0	2.2	0	2.6
A_52_P29953	NM_175398	6530418L21Rik	0	1.6	0	2	0	2.5
A_52_P675395	NM_007722	Cxcr7	0.41	1.3	0	1.6	0	2.5
A_51_P307168	NM_026993	Ddah1	0.02	1.7	0	2.2	0	2.5
A_55_P2137406	NM_007527	Bax	0	2	0	2.2	0	2.4
A_55_P1954835	XM_001475752	LOC100046186	0.06	1.6	0	1.8	0	2.4
A_52_P686785	NM_053247	Lyve1	0.35	1.5	0	2.1	0	2.4

A_51_P371750	NM_010766	Marco	0.54	1.6	0.03	2.1	0.02	2.4
A_51_P282760	NM_011066	Per2	0.25	1.6	0.02	1.8	0	2.4
A_52_P539310	NM_001160326	Serp2	0	1.8	0	2.3	0	2.4
A_51_P175580	NM_021897	Trp53inp1	0	1.7	0	2.2	0	2.4
A_51_P426353	NM_009463	Ucp1	0.99	-1	0.99	1	0.01	2.4
A_30_P01020960			0	1.9	0	2.1	0	2.3
A_30_P01024344			0.11	1.4	0	1.9	0	2.3
A_30_P01025511			0.24	1.4	0	1.8	0	2.3
A_55_P2143572	AK020725		0.13	1.4	0	1.7	0	2.3
A_51_P350403	NM_009148	Exoc4	0	1.6	0	2	0	2.3
A_55_P2101340	NM_019511	Ramp3	0.16	1.4	0	1.7	0	2.3
A_55_P2007713	NM_053082	Tspan4	0	1.8	0	2.2	0	2.3
A_51_P415220	NM_009517	Zmat3	0.07	1.4	0	1.8	0	2.3
A_30_P01027010			0	1.7	0	2	0	2.2
A_30_P01025790			0.08	1.4	0	1.7	0	2.2
A_30_P01032002			0.19	1.3	0	1.7	0	2.2
A_30_P01026923			0.08	1.8	0.05	1.6	0	2.2
A_30_P01019901			0.26	1.7	0.25	1.4	0.01	2.2
A_51_P110471	NM_026993	Ddah1	0.41	1.4	0	1.8	0	2.2
A_55_P2148534	BC096461	Nr1d2	0	2	0	2.3	0	2.2
A_52_P303891	NM_011584	Nr1d2	0	1.8	0	2	0	2.2
A_51_P329332	NM_054087	Slc19a2	0.2	1.4	0	1.8	0	2.2
A_30_P01029956			0	1.4	0	1.7	0	2.1
A_55_P1979893	NM_017376	Tef	0	1.9	0	1.9	0	2.1
A_51_P487073	NM_138684	Wfdc12	0.42	1.5	0	2.3	0	2.1
A_66_P115580	AK076360		0.02	1.5	0	1.7	0	2
A_66_P113325	XM_990419	5430427G11Rik	0.98	1.1	0.83	1.1	0.02	2
A_51_P279693	NM_009992	Cyp1a1	0.82	-1.2	0.4	-1.3	0.01	2
A_52_P311853	NM_030143	Ddit4l	0.14	1.3	0	1.5	0	2
A_55_P2269819	NM_183187	Fam107a	0.2	1.5	0	1.9	0	2
A_55_P2009752	NM_172563	Hlf	0.11	1.7	0.03	1.7	0.01	2
A_55_P2052563	NM_010495	Id1	0.63	1.4	0.07	1.6	0.01	2
A_55_P2071858	NM_008598	Mgmt	0.77	1.3	0.01	1.8	0	2
A_51_P258493	NM_011067	Per3	0	1.7	0	1.9	0	2
A_55_P2000533	NM_012048	Polk	0.02	1.5	0	1.6	0	2
A_51_P246903	NM_026467	Rps27l	0.09	1.5	0	1.8	0	2
A_55_P2094925	NM_011157	Srgn	0	1.5	0	1.7	0	2
A_30_P01025143			0.02	1.4	0	1.6	0	1.9
A_55_P1976204	U09507		0.05	1.4	0	1.6	0	1.9
A_51_P453736	NM_001143686	Apol11b	0.29	1.6	0.01	1.8	0.02	1.9
A_52_P627269	NM_198171	BC015286	0.31	1.5	0	2	0.01	1.9
A_55_P2052062	NM_010818	Cd200	0.99	1	0.04	1.5	0	1.9
A_51_P413785	NM_147778	Commd3	0	1.5	0	1.7	0	1.9
A_55_P2344608	AK081893	D7Wsu130e	0.24	1.4	0	1.6	0	1.9
A_51_P231320	NM_008611	Mmp8	0.53	1.3	0.01	1.7	0	1.9
A_51_P247184	NM_008728	Npr3	0.33	1.5	0.08	1.5	0.01	1.9
A_51_P290576	NM_152804	Plk2	0.04	1.4	0	1.6	0	1.9
A_52_P679105	NM_029614	Prss23	0.93	1.1	0.09	1.6	0.01	1.9
A_52_P198435	NM_207246	Rasgrp3	0.34	1.3	0	1.6	0	1.9
A_55_P2143025	NM_013657	Sema3c	0.55	1.2	0.38	1.2	0	1.9
A_55_P2119257	NM_008871	Serpine1	0.46	1.3	0	1.9	0	1.9
A_55_P2008936	NM_001102414	Slc2a9	0.11	1.4	0	1.8	0	1.9
A_55_P1965154	NM_025565	Spc25	0.07	1.5	0	1.9	0	1.9
A_55_P1973906	NM_021897	Trp53inp1	0.02	1.5	0	1.7	0	1.9
A_66_P136186	NM_009516	Wee1	0	1.4	0	1.5	0	1.9

A_55_P2039320	NM_178679	Zfp365	0.9	1.1	0.02	1.4	0	1.9
A_55_P2027812	NM_001166369	2900062L11Rik	0.89	1.2	0.56	1.3	0.04	1.8
A_55_P2136121	NM_145448	9030617O03Rik	0.02	1.8	0	1.9	0	1.8
A_55_P2405784	AK141429	BC023202	0.27	1.3	0	1.7	0	1.8
A_51_P460954	NM_009139	Ccl6	0.98	1	0.07	1.5	0.01	1.8
A_51_P109258	NM_138686	Cys1	0.79	1.3	0.37	1.3	0.03	1.8
A_66_P106132	XM_358529	E030044B06Rik	0.14	1.5	0.01	1.5	0	1.8
A_51_P455807	NM_133838	Ehd4	0.21	1.3	0	1.5	0	1.8
A_55_P2114779	NM_015764	Greb1	0.82	1.2	0.08	1.4	0	1.8
A_51_P516133	NM_015786	Hist1h1c	0.76	1.2	0.04	1.4	0	1.8
A_51_P354706	NM_010094	Lefty1	0.29	1.4	0	1.7	0	1.8
A_52_P167278	NM_172308	Mthfd1l	0.02	1.5	0	1.6	0	1.8
A_55_P1992084	NM_008981	Ptprg	0.17	1.4	0.04	1.4	0	1.8
A_55_P2107155	NM_029182	Rasd2	0.35	1.4	0.01	1.5	0	1.8
A_52_P236705	NM_133229	Ripply3	0.37	1.3	0.14	1.3	0	1.8
A_55_P2003813	NM_153522	Scn3b	0.72	1.2	0.1	1.4	0.01	1.8
A_51_P156438	NM_027460	Slc25a33	0.28	1.4	0.07	1.4	0	1.8
A_52_P40504	AK162948		0.14	1.4	0	1.6	0	1.7
A_30_P01026167			0.02	1.3	0	1.5	0	1.7
A_30_P01024850			0.41	1.2	0.01	1.4	0	1.7
A_51_P113178	NM_175398	6530418L21Rik	0.59	1.3	0	1.6	0	1.7
A_52_P151393	NM_198860	AI646023	0.3	1.2	0	1.3	0	1.7
A_55_P2110758	NM_177083	B430306N03Rik	0.68	1.3	0.12	1.4	0.01	1.7
A_55_P1964648	NM_001037719	Btla	0.09	1.7	0	1.8	0.02	1.7
A_52_P39083	NM_178793	Ccbe1	0.54	1.3	0.08	1.4	0.01	1.7
A_51_P204740	NM_133654	Cd34	0.6	1.3	0.14	1.3	0.01	1.7
A_52_P56636	NM_023186	Chia	0.27	1.6	0.47	1.3	0.03	1.7
A_55_P2038106	NM_009978	Cst8	0.11	1.6	0.32	1.3	0.02	1.7
A_52_P592909	NM_026384	Dgat2	0.73	1.2	0.07	1.4	0.01	1.7
A_55_P1998001	NM_001025384	DXBay18	0.82	1.2	0.11	1.4	0.01	1.7
A_51_P480328	NM_133222	Eltd1	0.77	1.2	0.06	1.4	0.01	1.7
A_55_P1988048	NM_001163522	Emcn	0.27	1.3	0.01	1.5	0	1.7
A_51_P214269	NM_023580	Epha1	0.5	1.3	0	1.7	0	1.7
A_51_P351896	NM_133187	Fam198b	0.52	1.2	0	1.6	0	1.7
A_51_P179697	NM_026884	Fam57b	0.7	1.2	0	1.6	0	1.7
A_55_P2180096	NR_003373	Gm15772	0.98	1	0.94	1	0.01	1.7
A_55_P2037343	NM_010391	H2-Q10	0.45	1.3	0.06	1.5	0.01	1.7
A_55_P2142251	NM_054045	Hist2h3c2	0.96	1.1	0.58	1.2	0.02	1.7
A_55_P2021114	NM_010500	Ier5	0	1.6	0	1.6	0	1.7
A_51_P294555	NM_001033632	Ifitm6	0.57	1.3	0	1.7	0.01	1.7
A_52_P281145	NM_172872	Kank4	0.15	1.4	0	1.8	0	1.7
A_55_P2006261	NM_008469	Krt15	0.24	1.3	0.01	1.5	0	1.7
A_55_P1954231	NM_172492	Lrtm2	0.54	1.3	0.13	1.4	0.02	1.7
A_55_P2015292	NM_008521	Ltc4s	0.99	1	0.02	1.6	0.01	1.7
A_55_P1975475	NM_010786	Mdm2	0.02	1.3	0	1.5	0	1.7
A_55_P1967291	NM_144818	Ncaph	0	1.5	0	1.7	0	1.7
A_52_P393314	NM_011027	P2rx7	0.06	1.3	0	1.5	0	1.7
A_55_P2037812	NM_023245	Palmd	0.02	1.5	0	1.5	0	1.7
A_55_P2087087	NM_001032378	Pecam1	0.08	1.3	0	1.6	0	1.7
A_55_P2095271	NM_153805	Pkn3	0.73	1.1	0.2	1.2	0	1.7
A_55_P2173313	NM_019677	Plcb1	0.35	1.4	0.06	1.4	0	1.7
A_51_P477682	NM_008939	Prss12	0.42	1.2	0.02	1.4	0	1.7
A_55_P2429225	NM_019976	Psrc1	0.93	1.1	0.05	1.3	0	1.7
A_55_P2071726	NM_029004	Rasgef1c	0.72	1.2	0	1.6	0	1.7
A_55_P2011146	NM_178608	Reep1	0.19	1.4	0.01	1.5	0	1.7

A_55_P2168628	NM_133678	Sac3d1	0.11	1.3	0	1.5	0	1.7
A_52_P87839	NM_013657	Sema3c	0.73	1.2	0.97	1	0.02	1.7
A_55_P2006008	NM_025429	Serpinb1a	0.45	1.2	0	1.5	0	1.7
A_51_P300572	NM_009579	Slc30a1	0.25	1.4	0.01	1.5	0.01	1.7
A_51_P299805	NM_027872	Slc46a3	0.02	1.4	0	1.5	0	1.7
A_51_P236267	NM_009183	St8sia4	0.3	1.3	0	1.6	0	1.7
A_55_P1963017	NM_001082543	Stfa1	0.79	1.2	0.09	1.4	0	1.7
A_55_P2168168	NM_031384	Tex11	0.02	1.6	0	1.8	0	1.7
A_55_P1986833	NM_054040	Tulp4	0.25	1.3	0.01	1.5	0	1.7
A_55_P1957245	XM_884146	Vmn2r-ps134	0.94	1.1	0.67	1.1	0.01	1.7
A_30_P01022991			0.04	1.4	0	1.5	0	1.6
A_52_P502849	BC108385		0.85	1.2	0.11	1.5	0.03	1.6
A_55_P2059765	AK087349		0.14	1.4	0.07	1.3	0.01	1.6
A_30_P01027569			0.91	-1.1	0.75	1.1	0	1.6
A_66_P138053	AK044848		0.42	1.2	0.17	1.2	0	1.6
A_30_P01033274			0.28	1.3	0	1.4	0	1.6
A_51_P369252	NM_001080995	4632434I11Rik	0.59	1.2	0.01	1.4	0	1.6
A_52_P489778	NM_178688	Ablim1	0.33	1.3	0.08	1.3	0	1.6
A_52_P487686	NM_001082546	BC100530	0.8	1.1	0.11	1.3	0.01	1.6
A_66_P111562	NM_007631	Ccnd1	0.72	1.1	0	1.4	0	1.6
A_51_P181286	NM_001033122	Cd69	0.66	1.2	0	1.5	0	1.6
A_52_P624434	NM_175526	Clec1a	0.02	1.6	0	1.8	0	1.6
A_55_P2107731	NM_028518	Col20a1	0.59	1.2	0.1	1.3	0.01	1.6
A_55_P2089488	NM_001039710	Coq10b	0.46	1.2	0	1.5	0	1.6
A_55_P2002968	NM_178893	Coro2a	0.85	1.1	0.42	1.2	0.01	1.6
A_52_P269942	NM_153679	Cpt1c	0.92	1	0.01	1.3	0	1.6
A_55_P1971729	NM_007758	Cr2	0.12	1.7	0.01	1.7	0.05	1.6
A_55_P2164534	NM_029766	Dtl	0.87	1.1	0.11	1.4	0.02	1.6
A_55_P1958887	NM_001025384	DXBay18	0.94	1.1	0.32	1.2	0.01	1.6
A_66_P104815	NM_007899	Ecm1	0.67	1.2	0.02	1.5	0.01	1.6
A_51_P405397	NM_007899	Ecm1	0.94	1.1	0.53	1.1	0.01	1.6
A_51_P449824	XM_001471750	Exoc3l2	0.56	1.2	0.03	1.4	0	1.6
A_55_P2043108	NM_009148	Exoc4	0.2	1.3	0.01	1.4	0	1.6
A_51_P172054	NM_019521	Gas6	0.82	1.2	0.19	1.3	0.02	1.6
A_55_P2056995	NM_008101	Gcgr	0.86	1.1	0.52	1.2	0.01	1.6
A_55_P2114776	NM_015764	Greb1	0.54	1.3	0.05	1.4	0.01	1.6
A_51_P449325	NM_008206	H2-Oa	0.11	1.6	0	1.9	0.01	1.6
A_55_P2082929	NM_010389	H2-Ob	0.59	1.3	0.05	1.5	0.04	1.6
A_51_P271603	NM_019424	Hps1	0.1	1.6	0.08	1.4	0.01	1.6
A_55_P1981949	NM_175174	Klhl5	0.28	1.3	0.11	1.3	0	1.6
A_55_P2192662	NM_001122899	Lepr	0.5	1.3	0.16	1.4	0.02	1.6
A_55_P2055257	NM_173740	Maoa	0.68	1.1	0.01	1.3	0	1.6
A_55_P1987914	NM_008696	Map4k4	0.27	1.2	0	1.6	0	1.6
A_52_P483336	NM_007641	Ms4a1	0.25	1.5	0.03	1.6	0.05	1.6
A_51_P479230	NM_023455	Nat8	0.66	1.2	0.17	1.3	0.02	1.6
A_51_P270184	NM_025811	Nhlrc2	0.09	1.4	0.06	1.3	0	1.6
A_51_P223776	NM_145434	Nr1d1	0.03	1.8	0	1.9	0.01	1.6
A_52_P111031	NM_001013753	Pcdh17	0.84	1.2	0.19	1.3	0.01	1.6
A_52_P327588	NM_148937	Plcd4	0.96	1	0.15	1.2	0	1.6
A_55_P2089233	NM_011136	Pou2af1	0.23	1.5	0.01	1.6	0.02	1.6
A_51_P332652	NM_172574	Pqlc3	0.63	1.1	0	1.4	0	1.6
A_55_P1995055	NM_001030296	Prr7	0.7	1.2	0.05	1.4	0.01	1.6
A_55_P2059010	NM_011254	Rbp1	0.09	1.5	0.07	1.3	0	1.6
A_55_P2051094	NM_011281	Rorc	0.61	1.2	0.68	1.1	0	1.6
A_55_P2083023	NM_133678	Sac3d1	0.25	1.2	0	1.4	0	1.6

A_51_P161354	NM_144907	Sesn2	0.13	1.4	0	1.5	0	1.6
A_51_P156434	NM_027460	Slc25a33	0.08	1.3	0	1.6	0	1.6
A_55_P1960238	NM_172659	Slc2a6	0.56	1.3	0.01	1.6	0	1.6
A_55_P1953301	NM_146126	Sord	0.12	1.4	0.01	1.4	0	1.6
A_55_P2027836	NM_020275	Tnfrsf10b	0.58	1.2	0.02	1.3	0	1.6
A_52_P220810	NM_144551	Trib2	0.26	1.3	0	1.4	0	1.6
A_51_P341918	NM_009366	Tsc22d1	0.12	1.3	0.01	1.3	0	1.6
A_55_P1985788	NM_198092	Usp2	0.24	1.3	0.21	1.2	0	1.6
A_30_P01028612			0.25	1.4	0	1.7	0.02	1.5
A_55_P2108693			0.85	1.1	0.32	1.2	0.01	1.5
A_30_P01026445			0.5	1.2	0.01	1.4	0	1.5
A_51_P378381	AK017076		0.16	1.3	0.02	1.3	0	1.5
A_30_P01026716			0.97	-1	0.14	1.2	0	1.5
A_30_P01019169			0.7	1.1	0.01	1.4	0	1.5
A_30_P01021048			0.23	1.2	0	1.4	0	1.5
A_30_P01018981			0.37	1.2	0.01	1.4	0	1.5
A_30_P01027299			0.29	1.3	0.02	1.3	0	1.5
A_55_P2026982	NM_173744	2610019F03Rik	0.21	1.4	0.06	1.3	0	1.5
A_55_P2115136	NM_028732	4632428N05Rik	0.6	1.2	0.01	1.4	0	1.5
A_51_P276479	NM_178098	4930486L24Rik	0.31	1.4	0.02	1.6	0.04	1.5
A_66_P122086	NM_001039720	9030619P08Rik	0.63	1.3	0.01	1.6	0.05	1.5
A_52_P301374	NM_153416	Aaas	0.71	1.2	0.02	1.4	0	1.5
A_55_P1988623	NM_001164099	Add3	0.2	1.3	0.01	1.3	0	1.5
A_55_P2169227	NM_177716	AI836003	0.21	1.4	0.01	1.5	0.01	1.5
A_55_P2408355	AK037728	BB166591	0.78	1.1	0.05	1.2	0	1.5
A_55_P1981836	NM_145536	BC020535	0.3	1.5	0.04	1.6	0.09	1.5
A_51_P424959	NM_007528	Bcl6b	0.85	1.1	0.06	1.3	0.01	1.5
A_51_P272553	NM_011498	Bhlhe40	0.8	1.1	0.09	1.3	0	1.5
A_51_P221651	NM_023341	Cabc1	0.03	1.4	0	1.4	0	1.5
A_52_P53906	NM_009829	Ccnd2	0.93	1.1	0.07	1.3	0	1.5
A_55_P2023542	NM_007719	Ccr7	0.58	1.3	0.02	1.5	0.04	1.5
A_55_P2040805	NM_130903	Cd209c	0.68	1.2	0.23	1.3	0.03	1.5
A_55_P2174490	NM_007645	Cd37	0.51	1.2	0	1.7	0.01	1.5
A_51_P342652	NM_008339	Cd79b	0.31	1.5	0.02	1.6	0.12	1.5
A_51_P272106	NM_007705	Cirbp	0.31	1.3	0.03	1.4	0.01	1.5
A_52_P162099	NM_001004140	Ckap2	0.17	1.4	0	1.6	0.02	1.5
A_52_P424784	NM_022319	Clstn2	0.49	1.3	0.16	1.3	0.02	1.5
A_55_P2032750	NM_153679	Cpt1c	0.93	1	0.01	1.3	0	1.5
A_51_P489522	NM_007797	Ctla2b	0.7	1.2	0.02	1.5	0.02	1.5
A_52_P70796	NM_007551	Cxcr5	0.73	1.2	0.03	1.5	0.02	1.5
A_51_P170641	NM_030246	Dcaf4	0.59	1.2	0	1.4	0	1.5
A_55_P2110497	NM_016672	Ddc	0.05	1.4	0.01	1.4	0.01	1.5
A_55_P1971709	NM_172400	Dnajc8	0.45	1.3	0.01	1.5	0.02	1.5
A_51_P419925	NM_001145922	E130112L23Rik	0.73	1.2	0.1	1.3	0.01	1.5
A_55_P2045258	NM_010119	Ehd1	0.2	1.3	0	1.5	0	1.5
A_55_P2134877	NM_001043335	Eml1	0.04	1.3	0	1.4	0	1.5
A_51_P435339	NM_010149	Epor	0.06	1.4	0	1.4	0	1.5
A_51_P405985	NM_133999	Fig4	0.36	1.2	0	1.4	0	1.5
A_55_P2110245	NM_001163359	Fignl1	0.79	1.2	0.06	1.4	0.04	1.5
A_52_P108447	NM_031184	Glis2	0.61	1.2	0.31	1.2	0.02	1.5
A_55_P2035326	XM_001473557	Gm2433	0.43	1.2	0.08	1.2	0	1.5
A_55_P1976744	XM_001478262	Gm4324	0.35	1.3	0	1.6	0.01	1.5
A_51_P401907	NM_001082547	Gm5483	0.89	1.1	0.61	1.2	0.03	1.5
A_55_P1965298	NM_001099302	Gm5640	0.82	1.2	0.33	1.3	0.04	1.5
A_55_P2068366	XM_895036	Gm9735	0.48	1.2	0.01	1.4	0.01	1.5

A_55_P2128646	NM_020567	Gmnn	0.26	1.3	0.07	1.3	0	1.5
A_51_P355753	NM_010430	Hic1	0.97	-1	0.96	-1	0.04	1.5
A_55_P2177154	NM_178200	Hist1h2bm	0.89	1.1	0.03	1.4	0.01	1.5
A_52_P515036	NM_016865	Htatip2	0.62	1.2	0.05	1.4	0.01	1.5
A_55_P2021109	NM_010500	Ier5	0.03	1.4	0	1.4	0	1.5
A_51_P433615	NM_183390	Klhl6	0.2	1.3	0	1.7	0	1.5
A_66_P118600	NM_008480	Lama1	0.85	1.1	0.26	1.2	0.02	1.5
A_55_P2042016	XM_001479435	LOC100048058	0.61	1.2	0.06	1.4	0.02	1.5
A_55_P2083481	NM_001130412	Lpin1	0.42	1.3	0	1.6	0.01	1.5
A_55_P2039250	NM_026037	Mboat2	0.71	1.1	0.01	1.4	0	1.5
A_55_P1986551	NM_023061	Mcam	0.27	1.3	0.01	1.5	0.01	1.5
A_55_P2005470	NM_029568	Mfap4	0	1.5	0	1.6	0.01	1.5
A_55_P2135986	NM_029499	Ms4a4c	0.59	1.3	0	1.8	0.02	1.5
A_52_P607128	NM_031195	Msr1	0.8	1.1	0.03	1.4	0.02	1.5
A_51_P386344	NM_172742	Mtmr10	0.22	1.3	0	1.4	0	1.5
A_55_P2157770	NM_001160165	Neu2	0.55	1.2	0.09	1.3	0	1.5
A_51_P515965	NM_008685	Nfe2	0.54	1.3	0	1.8	0.08	1.5
A_55_P2103452	NM_010934	Npy1r	0.59	1.2	0.3	1.2	0.01	1.5
A_51_P506733	NM_001038845	P2rx7	0.44	1.3	0.24	1.2	0.01	1.5
A_55_P2029191	NM_183408	Pde4a	0.62	1.2	0.06	1.4	0.03	1.5
A_51_P204442	NM_028716	Phf19	0.1	1.3	0	1.4	0	1.5
A_51_P156158	NM_133821	Phlpp1	0.71	1.2	0.06	1.4	0.02	1.5
A_55_P2129685	NM_001164053	Pkig	0.02	1.3	0	1.5	0	1.5
A_55_P2090359	NM_148937	Plcd4	0.94	-1.1	0.36	1.2	0.01	1.5
A_55_P1972297	NM_183152	Plk5	0.41	1.3	0.16	1.3	0.04	1.5
A_55_P2160094	NM_008880	Plscr2	0.13	1.3	0	1.4	0	1.5
A_55_P2236291	NM_008903	Ppap2a	0.45	1.3	0.09	1.4	0.03	1.5
A_52_P293120	NM_001081135	Prrg3	0.72	1.2	0.31	1.2	0.04	1.5
A_51_P148105	NM_011234	Rad51	0.91	1.1	0.07	1.4	0.02	1.5
A_55_P2036007	NM_198409	Rai2	0.31	1.3	0.02	1.4	0	1.5
A_52_P125467	NM_144865	Reep2	0.72	1.1	0.07	1.3	0.01	1.5
A_51_P249594	NM_019570	Rev1	0.33	1.2	0	1.5	0	1.5
A_55_P2078365	NM_173402	Rgs12	0.02	1.4	0	1.5	0	1.5
A_52_P89567	NM_007483	Rhob	0.99	1	0.08	1.3	0.01	1.5
A_51_P474454	NM_026001	Rnaseh2b	0.03	1.3	0	1.5	0	1.5
A_52_P577729	NM_175388	Rnf169	0.11	1.3	0.01	1.3	0	1.5
A_51_P502082	NM_009103	Rrm1	0.8	1.1	0.25	1.2	0.01	1.5
A_55_P2055232	NM_011341	Sdf4	0.28	1.3	0.01	1.4	0.01	1.5
A_52_P51786	NM_019934	Sec1	0.87	1.1	0.38	1.2	0.02	1.5
A_55_P2010152	NM_001164059	Sell	0.87	1.1	0.03	1.5	0.08	1.5
A_52_P73620	NM_198028	Serpinb10	0	1.6	0	1.7	0	1.5
A_55_P2029151	NM_144521	Snap47	0.28	1.3	0	1.5	0	1.5
A_55_P2131899	NM_009238	Sox4	0.7	1.1	0.04	1.3	0	1.5
A_51_P375862	NM_019684	Srpk3	0.47	1.3	0.01	1.6	0.04	1.5
A_55_P2031979	NM_010656	Sspn	0.62	1.2	0.2	1.2	0	1.5
A_55_P2152035	NM_001163763	Tcf19	0.43	1.2	0	1.4	0	1.5
A_55_P2047753	NM_001113460	Tec	0.02	1.3	0	1.5	0	1.5
A_55_P2018017	NM_009425	Tnfsf10	0.49	1.3	0.02	1.4	0.01	1.5
A_55_P2160686	NM_009366	Tsc22d1	0.17	1.3	0.02	1.4	0	1.5
A_55_P2069597	NM_028841	Tspan17	0.13	1.2	0	1.3	0	1.5
A_66_P114449	NM_147153	Vps39	0.14	1.3	0	1.5	0	1.5
A_55_P1976152	NM_027260	Vrk2	0.77	1.2	0.11	1.3	0.03	1.5
A_51_P311694	NM_027166	Ypel5	0.76	1.1	0.07	1.3	0.01	1.5
A_51_P312997	NM_012017	Zfp346	0.31	1.3	0.03	1.4	0.01	1.5
A_51_P431996	NM_175494	Zfp367	0.31	1.2	0.01	1.4	0	1.5

A_51_P485421	AK088666		0.53	1.3	0.02	1.6	0.22	1.4
A_55_P1983773	NM_001012273	Birc5	0.46	1.3	0.01	1.6	0.06	1.4
A_55_P1984825	NM_028607	Bloc1s2	0.41	1.2	0	1.5	0.01	1.4
A_51_P196526	NM_008144	Bscl2	0.57	1.2	0	1.5	0.01	1.4
A_55_P1960216	NM_009842	Cd151	0.92	1.1	0.04	1.5	0.16	1.4
A_55_P2132549	NM_007649	Cd48	0.98	1	0.01	1.5	0.01	1.4
A_55_P2124712	NM_145603	Ces2	0.72	1.1	0	1.5	0.01	1.4
A_55_P2150697	NM_028719	Cpne4	0.1	1.5	0.02	1.5	0.06	1.4
A_55_P2117345	NM_013517	Fcer2a	0.42	1.3	0	1.7	0.06	1.4
A_55_P1996973	NM_029000	Gvin1	0.96	1.1	0	2.1	0.16	1.4
A_66_P130916	NM_010389	H2-Ob	0.5	1.3	0.02	1.5	0.07	1.4
A_55_P1967539	NM_015755	Hunk	0.61	1.2	0.01	1.5	0.04	1.4
A_55_P2064862	NM_010492	Ica1	0.7	1.1	0	1.5	0	1.4
A_51_P336599	NM_020574	Kcne3	0.84	1.1	0.03	1.5	0.1	1.4
A_51_P130079	NM_008511	Lrmp	0.64	1.2	0.01	1.6	0.06	1.4
A_51_P265495	NM_010738	Ly6a	0.52	1.2	0	1.5	0.02	1.4
A_55_P2095311	XM_001475753	Ly6g	0.65	1.2	0	1.6	0.12	1.4
A_51_P253803	NM_001081117	Mki67	0.58	1.3	0.01	1.6	0.05	1.4
A_66_P106388	NM_029499	Ms4a4c	0.57	1.3	0.03	1.5	0.11	1.4
A_51_P296796	NM_027289	Nt5dc2	0.37	1.3	0.01	1.6	0.12	1.4
A_55_P1970033	NM_011065	Per1	0.23	1.4	0	1.8	0.07	1.4
A_55_P2116165	NM_133232	Pfkfb3	0.14	1.3	0	1.6	0	1.4
A_55_P2173982	NM_009104	Rrm2	0.52	1.2	0.02	1.5	0.03	1.4
A_51_P326191	NM_009251	Serpina3g	0.94	-1.1	0.01	1.9	0.26	1.4
A_66_P137654	NM_009213	Smpd2	0.07	1.3	0	1.5	0.01	1.4
A_55_P2089020	NM_001101483	Tmem22	0.45	1.5	0.03	1.7	0.22	1.4
A_52_P366105	NM_001033271	Tmem55b	0.15	1.3	0	1.5	0	1.4
A_51_P243808	NM_153137	Traf3ip3	0.24	1.4	0	1.6	0.06	1.4
A_51_P269203	NM_022017	Trpv4	0.89	1.2	0.04	1.7	0.25	1.4
A_66_P119968	NM_199311	Clec4a1	0.22	1.2	0	1.5	0.02	1.3
A_51_P264527	NM_019833	Fam69b	0.66	1.2	0.01	1.5	0.06	1.3
A_55_P2091193	XM_001480396	LOC100048513	0.35	1.3	0.01	1.5	0.11	1.3
A_51_P464182	NM_133653	Mat1a	0.41	1.4	0.04	1.5	0.39	1.3
A_51_P311958	NM_013623	Orm3	0.06	1.7	0.02	1.5	0.31	1.3
A_51_P230098	NM_023209	Pbk	0.74	1.2	0.02	1.5	0.17	1.3
A_51_P477121	NM_021451	Pmaip1	0.34	1.3	0.01	1.5	0.1	1.3
A_55_P2077681	NM_153744	Prkag3	0.42	1.2	0	1.5	0.02	1.3
A_55_P2002129	NM_173402	Rgs12	0.02	1.4	0	1.5	0.01	1.3
A_55_P2184905	NM_144911	Rpap2	0.31	1.3	0.01	1.5	0.1	1.3
A_55_P2104975	NM_001168294	Serpina3f	0.99	-1	0.02	1.8	0.45	1.3
A_51_P100327	NM_013683	Tap1	0.76	1.1	0	1.5	0.03	1.3
A_55_P2137421	NM_022017	Trpv4	0.92	1.1	0.02	1.6	0.39	1.3
A_55_P2057936	NM_001135115	Gm12250	0.85	-1.2	0.03	1.6	0.52	1.2
A_55_P1955015	NM_178440	Myo1g	0.65	1.1	0	1.5	0.11	1.2
A_55_P1962305	NM_139198	Plac8	0.88	1.1	0	1.7	0.39	1.2
A_55_P1962304	NM_139198	Plac8	0.91	1.1	0.03	1.5	0.52	1.2
A_51_P455897	NM_144526	Fam64a	0.6	1.3	0.03	1.5	0.66	1.1
A_55_P2169356	XM_001000891	Gm1966	1	-1	0.04	1.6	0.74	1.1
A_52_P128691	NM_175668	Gpr4	0.85	1.1	0.01	1.5	0.45	1.1
A_55_P2148046	XM_001478042	LOC100047388	0.81	1.1	0	1.5	0.44	1.1
A_55_P2052834	NM_010734	Lst1	0.91	1.1	0.03	1.5	0.64	1.1
A_30_P01026540			0.04	-1.6	1	-1	0.96	-1
A_30_P01028574			0.04	-1.9	0.97	-1	0.84	-1.1
A_30_P01019464			0.04	-2.4	1	1	0.93	-1.1
A_30_P01031515			0.89	1.1	0.04	1.5	0.83	-1.1

A_55_P1975832	NM_026939	1810009N02Rik	0.84	-1.4	0.01	-2.8	0.92	-1.1
A_55_P2136732	NM_025584	Cd99	0.59	-1.7	0.01	-2.7	0.9	-1.1
A_52_P650473	XM_913285	Gm7209	0.56	1.3	0.04	1.5	0.88	-1.1
A_55_P2157023	NM_011441	Sox17	0.87	-1.1	0	-1.6	0.56	-1.1
A_30_P01017834			0.74	1.2	0.02	-1.5	0.62	-1.2
A_30_P01031842			0.58	1.3	0.01	-1.5	0.39	-1.2
A_66_P116657	NM_178755	Agbl2	0.87	-1.1	0.02	-1.6	0.66	-1.2
A_55_P2080074	XM_001475946	Ahnak2	0.02	-1.5	0	-1.5	0.07	-1.2
A_51_P380750	NM_009824	Cbfa2t3	0.63	-1.2	0.01	-1.5	0.43	-1.2
A_51_P153423	NM_001081416	Fndc1	0.88	-1.1	0.02	-1.6	0.39	-1.2
A_55_P1995745	NM_001099349	Gm14308	0.04	-1.3	0	-1.5	0.03	-1.2
A_55_P2063594	NR_002889	Gm5801	0.47	1.7	0.01	2.5	0.73	-1.2
A_51_P393518	NM_001024720	Hmcn1	0.94	-1.1	0	-1.6	0.2	-1.2
A_55_P2032955	NM_001007462	Pabpn1l	0.72	-1.2	0.01	-1.6	0.47	-1.2
A_52_P682382	NM_009127	Scd1	0.48	-1.3	0.03	-1.5	0.38	-1.2
A_51_P224164	NM_011867	Slc26a4	0.02	-2.2	0.02	-1.8	0.58	-1.2
A_30_P01031630			0	-2	0.29	-1.3	0.28	-1.3
A_30_P01018533			0.86	1.1	0.03	-1.5	0.19	-1.3
A_30_P01022497			0.77	-1.2	0.01	-1.5	0.08	-1.3
A_30_P01018186			0.07	-1.3	0	-1.5	0.02	-1.3
A_30_P01019643			0.51	-1.3	0.02	-1.6	0.29	-1.3
A_30_P01026062			0.53	-1.3	0.02	-1.6	0.16	-1.3
A_30_P01029468			0.65	-1.2	0.01	-1.6	0.11	-1.3
A_30_P01019556			0.6	-1.3	0.01	-1.6	0.22	-1.3
A_55_P2145376	AK164317		0.55	-1.2	0	-1.6	0.03	-1.3
A_30_P01029645			0.39	-1.4	0.01	-1.7	0.25	-1.3
A_55_P2209053	AK013461	2900001G08Rik	0.67	-1.2	0.01	-1.5	0.11	-1.3
A_55_P2085905	NM_008486	Anpep	0.5	-1.2	0.01	-1.5	0.06	-1.3
A_55_P2086075	NM_001170345	Gm2058	0.05	-1.4	0	-1.5	0.02	-1.3
A_55_P2054708	XM_001473132	LOC100044824	0.14	-1.5	0.02	-1.6	0.28	-1.3
A_51_P167527	NM_008524	Lum	0.29	-1.5	0.02	-1.6	0.36	-1.3
A_55_P2313033	NM_029928	Ptprb	0.61	-1.2	0.01	-1.5	0.12	-1.3
A_55_P2052535	NM_001085546	RP23-330D3.5	0.17	-1.3	0	-1.5	0.02	-1.3
A_52_P670812	NR_028575	Snord123	0.6	-1.3	0.02	-1.7	0.32	-1.3
A_51_P208697	NM_027192	Ttl	0.2	-1.2	0	-1.5	0.01	-1.3
A_30_P01020128			0.37	-1.3	0.02	-1.5	0.02	-1.4
A_30_P01026949			0.37	-1.3	0.01	-1.5	0.05	-1.4
A_30_P01032544			0.46	-1.3	0.01	-1.5	0.03	-1.4
A_52_P16356	AK013505		0.39	-1.3	0	-1.5	0.06	-1.4
A_30_P01031627			0.39	-1.3	0	-1.5	0.03	-1.4
A_55_P1995774			0.02	-1.3	0	-1.5	0	-1.4
A_30_P01031578			0.14	-1.3	0	-1.5	0.01	-1.4
A_52_P262676			0.42	-1.2	0	-1.6	0.03	-1.4
A_30_P01032901			0.13	-1.4	0	-1.6	0.01	-1.4
A_30_P01018650			0.5	-1.5	0.02	-1.8	0.38	-1.4
A_55_P2089304	NR_024329	2900097C17Rik	0.05	-1.3	0	-1.5	0	-1.4
A_55_P1976694	NM_001009818	41893.0	0	-1.3	0	-1.5	0	-1.4
A_55_P2316612	AK020106	6720407P12Rik	0.81	-1.2	0.03	-1.5	0.13	-1.4
A_55_P2167803	NM_001014423	Abi3bp	0.55	-1.3	0.01	-1.6	0.1	-1.4
A_52_P482897	NM_009704	Areg	0.04	-1.8	0	-1.8	0.21	-1.4
A_55_P2066116	NM_033601	Bcl3	0	-1.5	0.01	-1.5	0.01	-1.4
A_51_P182303	NM_007743	Col1a2	0.34	-1.3	0.01	-1.5	0.04	-1.4
A_51_P378789	NM_018866	Cxcl13	0.23	-1.4	0.01	-1.5	0.06	-1.4
A_55_P2112967	NM_007858	Diap1	0	-1.4	0	-1.6	0	-1.4
A_55_P1995768	NM_001033123	Gm14288	0.45	-1.3	0.02	-1.5	0.12	-1.4

A_55_P1996086	NM_001099308	Gm14391	0.03	-1.3	0	-1.5	0	-1.4
A_52_P900300	XM_001472981	Gm6213	0	-2.4	0.19	-1.4	0.27	-1.4
A_52_P22590	NM_001024720	Hmcn1	0.34	-1.3	0	-1.6	0.02	-1.4
A_52_P176737	NM_178142	Lcorl	0.33	-1.3	0.01	-1.5	0.04	-1.4
A_52_P243054	NM_001024205	Nufip2	0.1	-1.3	0	-1.5	0	-1.4
A_51_P340699	NM_026864	Rasl11a	0.31	-1.3	0.01	-1.5	0.04	-1.4
A_51_P281089	NM_011313	S100a6	0.31	-1.4	0	-1.7	0.13	-1.4
A_55_P2005873	NM_146224	Suhw4	0.25	-1.3	0	-1.5	0.01	-1.4
A_51_P501364	NM_019507	Tbx21	0.28	-1.4	0.04	-1.5	0.13	-1.4
A_55_P1994032	NM_013842	Xbp1	0.48	-1.2	0	-1.5	0	-1.4
A_55_P2107192			0.23	-1.4	0.02	-1.5	0.03	-1.5
A_30_P01022683			0.73	-1.1	0.01	-1.5	0	-1.5
A_30_P01018802			0.85	-1.1	0.01	-1.5	0.03	-1.5
A_30_P01026526			0.72	-1.2	0.01	-1.5	0.01	-1.5
A_30_P01028032			0.48	-1.3	0.01	-1.5	0.02	-1.5
A_30_P01026605			0.04	-1.4	0	-1.5	0	-1.5
A_30_P01020373			0.3	-1.3	0	-1.5	0	-1.5
A_30_P01031683			0.11	-1.3	0	-1.5	0	-1.5
A_30_P01019947			0.14	-1.3	0	-1.5	0	-1.5
A_30_P01030384			0.11	-1.3	0	-1.5	0	-1.5
A_30_P01020487			0.18	-1.3	0	-1.5	0	-1.5
A_55_P2112302			0.12	-1.4	0	-1.5	0.01	-1.5
A_30_P01029348			0.29	-1.3	0	-1.5	0	-1.5
A_30_P01027949			0.07	-1.3	0	-1.5	0	-1.5
A_30_P01026040			0.37	-1.2	0	-1.5	0	-1.5
A_30_P01026332			0.11	-1.3	0	-1.5	0	-1.5
A_55_P2184551	AK050822		0	-1.4	0	-1.5	0	-1.5
A_30_P01028808			0.1	-1.3	0	-1.6	0	-1.5
A_30_P01026623			0.2	-1.4	0.06	-1.4	0.04	-1.5
A_30_P01022638			0.76	-1.2	0.13	-1.4	0.04	-1.5
A_52_P313285	AF108396		0.58	-1.2	0.33	-1.2	0.03	-1.5
A_55_P2150224			0.78	-1.2	0.25	-1.3	0.03	-1.5
A_30_P01033617			0.63	-1.3	0.06	-1.4	0.03	-1.5
A_30_P01028945			0.8	-1.1	0.44	-1.2	0.02	-1.5
A_55_P2157788			0.64	-1.2	0.13	-1.3	0.02	-1.5
A_30_P01031278			0.14	-1.5	0.1	-1.3	0.02	-1.5
A_30_P01023029			0.81	-1.1	0.08	-1.3	0.02	-1.5
A_30_P01019544			0.28	-1.3	0.09	-1.3	0.01	-1.5
A_55_P2030183			0.56	-1.2	0.05	-1.3	0.01	-1.5
A_55_P2013996	XM_001004714		0.94	1.1	0.22	-1.2	0.01	-1.5
A_30_P01029702			0.95	-1.1	0.04	-1.4	0.01	-1.5
A_55_P1959624	AK157913		0.44	-1.2	0.02	-1.4	0.01	-1.5
A_30_P01031680			0.33	-1.2	0.01	-1.4	0.01	-1.5
A_55_P1982744			0.51	-1.2	0.05	-1.3	0.01	-1.5
A_30_P01019455			0.58	-1.2	0.05	-1.3	0.01	-1.5
A_30_P01028215			0.41	-1.3	0.01	-1.4	0.01	-1.5
A_30_P01028210			0.59	-1.2	0.03	-1.4	0.01	-1.5
A_30_P01022115			0.48	-1.2	0.01	-1.4	0.01	-1.5
A_30_P01023888			0.64	-1.2	0.03	-1.4	0.01	-1.5
A_55_P2109564	AK039146		0.25	-1.2	0	-1.4	0	-1.5
A_30_P01031878			0.24	-1.3	0.01	-1.4	0	-1.5
A_30_P01029711			0.51	-1.2	0	-1.4	0	-1.5
A_30_P01030728			0.15	-1.3	0	-1.4	0	-1.5
A_30_P01024724			0.35	-1.3	0.02	-1.4	0	-1.5
A_30_P01023623			0.07	-1.5	0.08	-1.3	0	-1.5

A_55_P2104387			0.03	-1.3	0	-1.4	0	-1.5
A_55_P2182939			0.55	-1.2	0.03	-1.3	0	-1.5
A_30_P01027850			0.16	-1.2	0	-1.3	0	-1.5
A_55_P2140390			0.3	-1.2	0.02	-1.3	0	-1.5
A_55_P2065113	AK017437		0.91	1.1	0.48	-1.1	0	-1.5
A_30_P01018597			0.6	-1.2	0.01	-1.3	0	-1.5
A_30_P01030896			0.29	-1.2	0	-1.4	0	-1.5
A_55_P2013780			0.58	-1.2	0.08	-1.3	0	-1.5
A_66_P140613			0.04	-1.3	0	-1.4	0	-1.5
A_30_P01027329			0.17	-1.3	0	-1.4	0	-1.5
A_30_P01023773			0.49	-1.2	0.03	-1.3	0	-1.5
A_30_P01029815			0.2	-1.3	0.01	-1.4	0	-1.5
A_30_P01021211			0.29	-1.3	0.01	-1.4	0	-1.5
A_30_P01027180			0.79	-1.1	0.08	-1.3	0	-1.5
A_30_P01020467			0.24	-1.2	0	-1.4	0	-1.5
A_55_P2180056	AK158492		0.17	-1.3	0	-1.4	0	-1.5
A_30_P01031405			0	-1.3	0	-1.4	0	-1.5
A_30_P01033110			0.41	-1.2	0.01	-1.4	0	-1.5
A_30_P01027463			0.34	-1.2	0.01	-1.4	0	-1.5
A_55_P2003199	AK141316		0.72	-1.2	0.07	-1.3	0	-1.5
A_30_P01030970			0.31	-1.3	0.08	-1.3	0	-1.5
A_55_P2049158			0.65	-1.2	0.08	-1.3	0	-1.5
A_30_P01026329			0.04	-1.4	0.01	-1.4	0	-1.5
A_55_P2003494			0.51	-1.2	0.02	-1.4	0	-1.5
A_30_P01028021			0.48	-1.2	0	-1.4	0	-1.5
A_30_P01019565			0.19	-1.2	0	-1.4	0	-1.5
A_55_P2318934	AK004627	1200007C13Rik	0.19	-1.4	0.04	-1.4	0	-1.5
A_55_P2425661	NM_025818	1200014J11Rik	0.25	-1.2	0	-1.5	0	-1.5
A_51_P199352	XM_483917	2310015B20Rik	0.19	-1.3	0.01	-1.4	0	-1.5
A_51_P439085	NM_023516	2310016C08Rik	0.14	-1.3	0.01	-1.4	0	-1.5
A_55_P2058783	NM_023516	2310016C08Rik	0.27	-1.2	0	-1.4	0	-1.5
A_51_P192089	NM_028228	2610028A01Rik	0.58	-1.2	0.03	-1.4	0.01	-1.5
A_55_P2281624	AK142273	2610034E01Rik	0.57	-1.2	0.01	-1.4	0	-1.5
A_55_P2273929	AK076353	4732457N14	0.45	-1.2	0.01	-1.4	0	-1.5
A_55_P2133646	NM_199316	4922501C03Rik	0.67	-1.2	0.22	-1.3	0.03	-1.5
A_55_P2230506	AK016424	4931402H11Rik	0.1	-1.3	0	-1.4	0	-1.5
A_55_P1976682	AK016539	4932431P20Rik	0.53	-1.2	0.11	-1.3	0.01	-1.5
A_55_P1996927	NR_033123	4933409K07Rik	0.82	-1.1	0.01	-1.4	0	-1.5
A_52_P40954	NR_028300	5330426P16Rik	0.17	-1.3	0	-1.5	0	-1.5
A_51_P474169	NM_144883	5430407P10Rik	0.1	-1.3	0	-1.4	0	-1.5
A_55_P2278965	AK017326	5430420F09Rik	0.45	-1.2	0.01	-1.3	0	-1.5
A_55_P2098275	XM_001474884	5730416O20Rik	0.9	-1.1	0.43	-1.2	0.02	-1.5
A_55_P2328787	AK080477	6030451C04Rik	0.99	-1	0.06	-1.3	0.01	-1.5
A_55_P2103536	NM_145836	6430527G18Rik	0.04	-1.4	0	-1.5	0	-1.5
A_55_P2097962	XM_001481064	9030622O22Rik	0.62	-1.2	0.15	-1.3	0.02	-1.5
A_52_P146403	NM_029953	9130221D24Rik	0.91	-1.1	0.25	-1.3	0.03	-1.5
A_55_P2162782	NM_009643	Ahnak	0.3	-1.3	0.01	-1.5	0.02	-1.5
A_51_P400543	NM_019467	Aif1	0.44	-1.3	0.53	-1.2	0.04	-1.5
A_55_P2012999	NM_170728	Ank3	0.58	-1.2	0.01	-1.4	0	-1.5
A_52_P79187	NM_001025572	Ankrd12	0.74	-1.1	0.01	-1.2	0	-1.5
A_55_P2043367	NM_173786	Apol9a	0.34	-1.4	0.21	-1.3	0.03	-1.5
A_66_P110633	NM_001168660	Apol9b	0.31	-1.4	0.15	-1.3	0.03	-1.5
A_51_P475228	NM_133972	Armc6	0.4	-1.4	0.67	-1.1	0.04	-1.5
A_51_P111612	NM_001042592	Arrdc4	0.23	-1.3	0	-1.6	0	-1.5
A_52_P194316	NM_001128094	Atp13a3	0.34	-1.2	0	-1.5	0	-1.5

A_52_P168097	NM_009722	Atp2a2	0.8	-1.1	0.02	-1.4	0.02	-1.5
A_55_P2172022	NM_022305	B4galt1	0.11	-1.4	0.02	-1.3	0	-1.5
A_55_P2360427	NM_001166581	BC005561	0.22	-1.2	0	-1.4	0	-1.5
A_55_P2258366	AK081218	C030009H01Rik	0	-1.4	0	-1.4	0	-1.5
A_55_P2037618	NM_001163502	C130039O16Rik	0.2	-1.3	0	-1.4	0	-1.5
A_55_P2164714	XM_356827	C7	0.48	-1.3	0.05	-1.4	0.03	-1.5
A_52_P590474	NM_019937	Ccnl1	0.07	-1.3	0.01	-1.3	0	-1.5
A_51_P287100	NM_007663	Cdh16	0.57	-1.3	0.02	-1.5	0.01	-1.5
A_52_P120122	NM_177090	Cdh29	0.45	-1.3	0.05	-1.4	0.02	-1.5
A_55_P2120577	NM_010828	Cited2	0.6	-1.2	0.04	-1.4	0.02	-1.5
A_51_P302167	NM_172496	Cobl	0.72	-1.2	0.03	-1.4	0.02	-1.5
A_55_P2118520	NM_007742	Col1a1	0.3	-1.4	0	-1.8	0.04	-1.5
A_55_P2148402	NM_009946	Cplx2	0.88	-1.1	0.11	-1.4	0.02	-1.5
A_51_P180452	NM_133656	Crk	0.49	-1.2	0.01	-1.4	0	-1.5
A_52_P218976	NM_173369	Cyld	0.09	-1.3	0.01	-1.4	0	-1.5
A_52_P52263	NM_001044719	D17Wsu92e	0.45	-1.2	0.02	-1.4	0	-1.5
A_55_P2446231	AK042002	D730040F13Rik	0.51	-1.3	0.06	-1.4	0.01	-1.5
A_52_P355398	NM_177445	Dars	0.37	-1.3	0.04	-1.4	0.01	-1.5
A_52_P621617	NM_027287	Dnajb4	0.39	-1.3	0.01	-1.4	0.01	-1.5
A_55_P1994173	XM_001474573	EG622110	0.31	-1.4	0.01	-1.6	0.02	-1.5
A_55_P2002133	NM_153178	Eif2c2	0.31	-1.3	0.09	-1.3	0	-1.5
A_55_P2240823	NM_001128606	Epb4.1	0.72	-1.2	0	-1.8	0.02	-1.5
A_52_P5549	NM_001042501	Fam133b	0.1	-1.4	0.01	-1.3	0	-1.5
A_52_P437850	NM_024244	Fam13c	0.16	-1.4	0.01	-1.4	0	-1.5
A_51_P371485	NM_198017	Fam175b	0.29	-1.2	0	-1.4	0	-1.5
A_55_P2178247	NM_145413	Fam20b	0.48	-1.2	0.01	-1.4	0	-1.5
A_55_P2076984	NM_134082	Farp1	0.11	-1.3	0.01	-1.3	0	-1.5
A_55_P2019068	NM_022009	Flii	0.45	-1.2	0	-1.4	0	-1.5
A_55_P2153783	NM_010231	Fmo1	0.65	-1.2	0.05	-1.5	0.03	-1.5
A_55_P2183110	NM_008031	Fmr1	0.18	-1.3	0.01	-1.5	0	-1.5
A_52_P68702	NM_145148	Frmd4b	0.23	-1.3	0	-1.5	0	-1.5
A_51_P476209	NM_019569	Fscn3	0.43	-1.2	0.21	-1.2	0	-1.5
A_55_P2130905	XM_001471976	Gm1983	0.2	-1.2	0	-1.4	0	-1.5
A_55_P2124461	XM_001472850	Gm2251	0.33	-1.4	0.02	-1.5	0.05	-1.5
A_55_P1966383	XM_001475163	Gm2936	0.82	-1.1	0.07	-1.4	0.01	-1.5
A_55_P1998811	XM_001476688	Gm3430	0.24	-1.6	0.03	-1.7	0.16	-1.5
A_55_P1993049	XM_001478943	Gm4076	0.71	-1.2	0.07	-1.4	0.04	-1.5
A_55_P2159850	XM_001479402	Gm4191	0.86	-1.1	0.1	-1.4	0.02	-1.5
A_52_P30877	BC054110	Gm5176	0.64	-1.2	0.05	-1.3	0	-1.5
A_52_P658974	XM_358238	Gm5396	0.46	-1.2	0.04	-1.3	0	-1.5
A_55_P2134979	XM_884320	Gm6115	0.64	-1.2	0.09	-1.3	0.01	-1.5
A_55_P2106106	NM_176912	Gpr77	0.27	-1.3	0.14	-1.3	0.01	-1.5
A_55_P1955457	NM_010345	Grb10	0.2	-1.4	0	-1.5	0	-1.5
A_51_P407984	NM_030022	Grifin	0.32	-1.4	0.57	-1.2	0.04	-1.5
A_52_P655663	NM_010376	H13	0.11	-1.4	0.01	-1.4	0	-1.5
A_66_P108019	NM_144835	Heatr1	0.31	-1.2	0	-1.3	0	-1.5
A_55_P1972018	NM_178208	Hist1h4c	0.58	-1.2	0.34	-1.2	0.02	-1.5
A_55_P2057913	NM_001111282	Hmg1l1	0.67	-1.2	0.11	-1.3	0.01	-1.5
A_51_P347452	NM_028242	Htatsf1	0.55	-1.2	0.03	-1.3	0	-1.5
A_55_P1997951	NM_028242	Htatsf1	0.45	-1.2	0.01	-1.4	0	-1.5
A_51_P327632	NM_207205	Igsf3	0.42	-1.3	0	-1.6	0	-1.5
A_55_P2430122	NM_026374	Ilf2	0.13	-1.2	0	-1.4	0	-1.5
A_51_P419656	NM_020268	Klk1b27	0.22	-1.4	0.02	-1.4	0.01	-1.5
A_55_P2170350	NM_053152	Klra22	0.42	-1.3	0.23	-1.2	0.01	-1.5
A_55_P2118630	NM_010688	Lasp1	0.35	-1.3	0.01	-1.5	0.01	-1.5

A_51_P241210	NM_001081231	Lhfpl3	0.04	-1.6	0.14	-1.3	0.02	-1.5
A_66_P118165	XM_001471574	LOC100038935	0.5	-1.2	0	-1.5	0	-1.5
A_55_P2294037	XM_001475407	LOC100045958	0.35	-1.3	0	-1.6	0.01	-1.5
A_55_P2076524	XM_001476211	LOC100046441	0.57	-1.2	0.01	-1.3	0	-1.5
A_55_P2083149	XM_001476298	LOC100046485	0.26	-1.2	0.01	-1.3	0	-1.5
A_66_P112862	NR_033146	LOC100316870	0.82	-1.1	0.02	-1.4	0	-1.5
A_55_P2108486	XM_903408	LOC630284	0.25	-1.2	0	-1.4	0	-1.5
A_51_P397296	NM_028584	Marveld3	0.6	-1.2	0	-1.4	0	-1.5
A_55_P2448776	NM_001081392	Mdn1	0.44	-1.2	0.05	-1.3	0	-1.5
A_55_P2114863	NM_001166251	Mgll	0.71	-1.2	0.02	-1.5	0.02	-1.5
A_55_P2136526	NM_001033276	Mll2	0.74	-1.2	0.08	-1.4	0.02	-1.5
A_52_P180373	NM_010821	Mpeg1	0.12	-1.4	0	-1.5	0.01	-1.5
A_55_P2061645	AK140300	ND6	0.16	-1.3	0	-1.5	0	-1.5
A_51_P517870	NM_198429	Nfatc1	0.59	-1.2	0	-1.5	0.01	-1.5
A_55_P2077866	NM_010918	Nktr	0.3	-1.2	0	-1.4	0	-1.5
A_51_P147123	NM_008744	Ntn1	0	-1.3	0	-1.5	0	-1.5
A_51_P254262	NM_130858	Nxph3	0.35	-1.3	0	-1.6	0	-1.5
A_55_P2100799	NM_146275	Olfr1402	0.74	-1.1	0.01	-1.4	0.01	-1.5
A_55_P2134387	NM_020289	Olfr544	0.12	-1.3	0	-1.4	0	-1.5
A_55_P2117146	NM_011119	Pa2g4	0.2	-1.3	0.12	-1.3	0.01	-1.5
A_55_P2134581	NM_011112	Papola	0.33	-1.2	0.01	-1.4	0	-1.5
A_55_P1963533	NM_001003672	Pcdhac2	0.54	-1.3	0.37	-1.2	0.04	-1.5
A_55_P2128270	NM_138755	Phf21a	0.44	-1.3	0.02	-1.4	0	-1.5
A_52_P530291	NM_008842	Pim1	0.11	-1.3	0.01	-1.4	0	-1.5
A_55_P2212908	AK037585	Pon2	0.47	-1.1	0	-1.4	0	-1.5
A_51_P265008	NM_001081086	Ppig	0.55	-1.1	0	-1.4	0	-1.5
A_55_P2007944	NM_011104	Prkce	0.24	-1.3	0.01	-1.5	0	-1.5
A_51_P504314	NM_025845	Prpf38b	0.85	-1.1	0.09	-1.3	0.02	-1.5
A_55_P2132472	NM_013830	Prpf4b	0.11	-1.3	0	-1.4	0	-1.5
A_52_P28651	NM_021424	Pvrl1	0.79	-1.1	0.01	-1.4	0	-1.5
A_51_P247637	NM_080563	Rnf144a	0.48	-1.3	0.01	-1.6	0.03	-1.5
A_55_P2020361	NM_197945	RP23-100C5.8	0.87	-1.1	0.05	-1.4	0.01	-1.5
A_55_P2146185	NM_011302	Rs1	0.73	-1.1	0.01	-1.5	0	-1.5
A_55_P2020472	NM_027425	Rufy2	0.6	-1.1	0.06	-1.2	0	-1.5
A_66_P112065	AK142268	Sertad2	0.62	-1.1	0	-1.4	0	-1.5
A_55_P2021011	NM_030207	Sfi1	0.9	-1.1	0.08	-1.3	0.01	-1.5
A_55_P2448065	NM_053196	Sfxn2	0.56	-1.3	0.25	-1.3	0.04	-1.5
A_55_P2129867	NM_008018	Sh3pxd2a	0.11	-1.3	0	-1.4	0	-1.5
A_55_P2086885	NM_001033219	Slc45a4	0.08	-1.4	0	-1.5	0	-1.5
A_51_P112762	NM_017391	Slc5a3	0.35	-1.3	0.05	-1.3	0.01	-1.5
A_55_P2026125	NM_175229	Srrm2	0.14	-1.3	0	-1.6	0	-1.5
A_55_P2139913	NM_009278	Ssb	0	-1.3	0	-1.4	0	-1.5
A_52_P312398	NM_026155	Ssr3	0.31	-1.3	0	-1.5	0	-1.5
A_55_P2118609	NM_011371	St6galnac1	0.68	-1.2	0.02	-1.5	0.01	-1.5
A_55_P2144456	NM_001167884	Suv420h1	0.8	-1.1	0.03	-1.3	0.01	-1.5
A_55_P2462940	NM_001081008	Taf1	0.14	-1.3	0.01	-1.4	0	-1.5
A_52_P161297	NM_011542	Tcea3	0.3	-1.2	0	-1.4	0	-1.5
A_55_P1958246	NM_011552	Tcof1	0.25	-1.3	0.01	-1.4	0	-1.5
A_51_P449233	NM_172444	Thsd4	0.99	-1	0.37	-1.2	0.02	-1.5
A_55_P2161219	NM_001164805	Thsd7a	0.68	-1.2	0.03	-1.4	0.03	-1.5
A_55_P2138960	NM_013861	Tpk1	0.11	-1.3	0	-1.5	0	-1.5
A_55_P2138961	NM_013861	Tpk1	0.03	-1.4	0	-1.4	0	-1.5
A_55_P1968068	NM_011640	Trp53	0.22	-1.3	0.01	-1.4	0	-1.5
A_52_P274496	NM_183180	Tspan18	0.49	-1.3	0	-1.7	0.02	-1.5
A_55_P2156425	NM_026815	Upk1a	0.92	-1.1	0.3	-1.3	0.04	-1.5

A_52_P461343	NM_023478	Upk3a	0.52	-1.4	0	-2	0.15	-1.5
A_52_P543040	NM_028276	Utp14a	0.77	-1.2	0.01	-1.6	0.02	-1.5
A_55_P2212027	NM_011682	Utrn	0.49	-1.2	0.03	-1.4	0.01	-1.5
A_55_P2109057	NM_177354	Vash1	0.79	-1.1	0.16	-1.2	0	-1.5
A_52_P96782	NM_028459	Wasl	0.04	-1.3	0	-1.5	0	-1.5
A_55_P1987245	NM_011740	Ywhaz	0.29	-1.3	0	-1.6	0.01	-1.5
A_55_P2173373	NM_001024846	Zfp62	0.41	-1.2	0	-1.4	0	-1.5
A_30_P01021078			0	-1.5	0	-1.7	0	-1.6
A_30_P01028666			0.89	-1.1	0.02	-1.5	0.01	-1.6
A_30_P01021970			0.28	-1.3	0.01	-1.5	0.01	-1.6
A_30_P01029062			0.13	-1.4	0.01	-1.5	0	-1.6
A_30_P01030969			0.18	-1.4	0.01	-1.5	0	-1.6
A_66_P131433			0.45	-1.3	0.01	-1.5	0	-1.6
A_30_P01032612			0.11	-1.3	0	-1.5	0	-1.6
A_30_P01019049			0.12	-1.3	0	-1.5	0	-1.6
A_30_P01028970			0.06	-1.3	0	-1.5	0	-1.6
A_55_P1955733	XM_001477303		0.13	-1.3	0	-1.5	0	-1.6
A_30_P01032760			0.07	-1.3	0	-1.5	0	-1.6
A_55_P2170834	CA461216		0.26	-1.3	0	-1.5	0	-1.6
A_55_P2021923	AB241120		0.12	-1.4	0	-1.5	0	-1.6
A_30_P01023400			0.13	-1.4	0	-1.5	0	-1.6
A_30_P01025909			0.12	-1.3	0	-1.5	0	-1.6
A_30_P01022987			0.07	-1.3	0	-1.5	0	-1.6
A_30_P01020298			0.13	-1.3	0	-1.5	0	-1.6
A_30_P01029602			0.35	-1.2	0	-1.5	0	-1.6
A_30_P01030106			0.03	-1.4	0	-1.5	0	-1.6
A_30_P01025590			0.2	-1.4	0.01	-1.6	0	-1.6
A_55_P1960148	XM_001472091		0.3	-1.5	0.01	-1.7	0.04	-1.6
A_30_P01017919			0.15	-1.7	0.01	-1.7	0.07	-1.6
A_30_P01019829			0	-1.4	0	-1.7	0	-1.6
A_30_P01033043			0.48	-1.6	0.01	-2	0.21	-1.6
A_55_P2021476	XM_001474821		0.82	-1.2	0.06	-1.5	0.04	-1.6
A_30_P01024129			0.84	-1.2	0.31	-1.3	0.04	-1.6
A_30_P01022168			0.32	-1.4	0.14	-1.4	0.03	-1.6
A_55_P1956762	AK082974		0.82	-1.2	0.16	-1.4	0.03	-1.6
A_30_P01031754			0.42	-1.3	0.03	-1.4	0.01	-1.6
A_55_P2003492			0.74	-1.2	0.12	-1.3	0.01	-1.6
A_55_P1952312			0.51	-1.3	0.05	-1.4	0	-1.6
A_30_P01031360			0.41	-1.3	0.02	-1.4	0	-1.6
A_30_P01030704			0.98	1	0.02	-1.4	0	-1.6
A_55_P2112549			0.58	-1.2	0.04	-1.4	0	-1.6
A_55_P2064025	XM_001474990		0.66	-1.2	0.01	-1.4	0	-1.6
A_52_P501049	BC002059		0.2	-1.2	0	-1.4	0	-1.6
A_30_P01024550			0.18	-1.3	0.05	-1.3	0	-1.6
A_55_P1991239	AK051413		0.42	-1.2	0	-1.4	0	-1.6
A_30_P01028770			0.24	-1.3	0.05	-1.3	0	-1.6
A_55_P2074081			0.57	-1.2	0.02	-1.4	0	-1.6
A_55_P2002220	AK129256		0.58	-1.2	0.11	-1.3	0	-1.6
A_30_P01031736			0.53	-1.2	0.01	-1.4	0	-1.6
A_52_P447424			0.6	-1.2	0	-1.4	0	-1.6
A_55_P2043531			0.58	-1.2	0.02	-1.4	0	-1.6
A_55_P2178137	NM_011889	41885.0	0.22	-1.3	0	-1.7	0	-1.6
A_55_P2002449	XM_001473799	4932422M17Rik	0.03	-1.5	0	-1.7	0	-1.6
A_55_P1977741	XM_001473799	4932422M17Rik	0.02	-1.5	0	-1.6	0	-1.6
A_55_P1973347	NR_002860	A130040M12Rik	0.33	-1.3	0	-1.5	0	-1.6

A_55_P1978316	NM_001081127	Adamts14	0.3	-1.3	0.03	-1.4	0	-1.6
A_55_P2266977	NM_021414	Ahcyl2	0.05	-1.3	0	-1.6	0	-1.6
A_52_P351925	NM_146005	Ank3	0.35	-1.3	0.01	-1.5	0	-1.6
A_55_P1977875	NM_176849	Arglu1	0.31	-1.3	0	-1.5	0	-1.6
A_51_P484842	NM_007493	Asgr2	0.3	-1.5	0.11	-1.4	0.03	-1.6
A_51_P114616	NM_016767	Batf	0.24	-1.3	0	-1.4	0	-1.6
A_55_P1970537	NM_009745	Bcl7b	0.33	-1.3	0.01	-1.4	0	-1.6
A_55_P1985259	NM_001017985	C2cd3	0.04	-1.3	0	-1.6	0	-1.6
A_55_P2033215	NM_013877	Cabp5	0.84	-1.1	0.02	-1.4	0.01	-1.6
A_55_P2126662	NM_007616	Cav1	0.03	-1.4	0	-1.6	0	-1.6
A_51_P145662	NM_029465	Clec4g	0.95	1	0.7	-1.1	0	-1.6
A_55_P1962603	NM_009946	Cplx2	0.9	-1.1	0.12	-1.4	0.02	-1.6
A_51_P124345	NM_153166	Cpne5	0.78	-1.2	0.12	-1.4	0.02	-1.6
A_55_P1962209	NM_030712	Cxcr6	0.53	-1.3	0.1	-1.4	0.01	-1.6
A_51_P144349	NM_172442	Dtx4	0.28	-1.3	0.08	-1.3	0	-1.6
A_52_P493620	NM_026218	Fgfr1op2	0.65	-1.3	0.03	-1.6	0.03	-1.6
A_51_P283473	NM_026271	Fibin	1	-1	0.03	-1.6	0.04	-1.6
A_52_P493091	NM_008031	Fmr1	0.13	-1.3	0	-1.5	0	-1.6
A_52_P233441	NM_008090	Gata2	0.02	-1.4	0	-1.7	0	-1.6
A_55_P2096967	NM_008124	Gjb1	0.19	-1.4	0.01	-1.4	0	-1.6
A_55_P2152188	XM_001473965	Gm2556	0.08	-1.3	0	-1.6	0	-1.6
A_55_P2048867	XM_001475122	Gm2922	0.3	-1.4	0.01	-1.5	0	-1.6
A_55_P1992571	XM_001475200	Gm2952	0.93	-1.1	0.05	-1.4	0.02	-1.6
A_55_P2047620	XM_001475740	Gm3124	0.67	-1.2	0.01	-1.6	0.01	-1.6
A_55_P1961760	XM_001476058	Gm3237	0.73	-1.3	0.04	-1.7	0.06	-1.6
A_55_P2058467	XM_001479915	Gm4415	0.31	-1.2	0	-1.5	0	-1.6
A_55_P2009042	XM_001481023	Gm4635	0.38	-1.2	0.01	-1.4	0	-1.6
A_55_P2077497	XM_001476406	Gm6940	0.64	-1.3	0.01	-1.7	0.02	-1.6
A_55_P1991783	XM_001477578	Gm7149	0.58	-1.3	0	-1.7	0.01	-1.6
A_55_P2046728	XM_981891	Gm7792	0.65	-1.2	0.02	-1.4	0	-1.6
A_66_P127262	XM_985872	Gm8799	0.27	-1.3	0	-1.4	0	-1.6
A_55_P2101944	XM_001479912	Gm9444	0.5	-1.3	0.01	-1.6	0.01	-1.6
A_51_P128463	NM_001099296	Grrp1	0.73	-1.2	0.01	-1.6	0.03	-1.6
A_55_P2119917	NM_011772	Ikzf4	0.21	-1.4	0.11	-1.3	0.01	-1.6
A_55_P2092492	NM_001161842	Il18r1	0.85	-1.2	0.03	-1.6	0.02	-1.6
A_55_P1957593	NM_023547	Ino80b	0.58	-1.1	0	-1.4	0	-1.6
A_55_P2040011	NM_008398	Itga7	0.39	-1.4	0.02	-1.5	0.02	-1.6
A_55_P2004526	NM_010650	Klra8	0.61	-1.3	0.36	-1.3	0.03	-1.6
A_55_P1981455	XM_001472240	LOC100044430	0.3	-1.4	0.18	-1.3	0.02	-1.6
A_55_P2009058	XM_001480449	LOC100048584	0	-1.4	0	-1.6	0	-1.6
A_55_P2048660	XM_908118	LOC633654	0.25	-1.3	0	-1.5	0	-1.6
A_66_P106133	NM_133853	Magi3	0.31	-1.3	0	-1.6	0	-1.6
A_52_P79385	NM_025952	Magt1	0.02	-1.3	0	-1.4	0	-1.6
A_55_P2062642	NM_025952	Magt1	0.1	-1.4	0	-1.4	0	-1.6
A_52_P547187	NM_138667	Map3k7ip2	0.33	-1.2	0.01	-1.4	0	-1.6
A_55_P2081388	NM_008575	Mdm4	0.15	-1.4	0.01	-1.5	0	-1.6
A_55_P2181597	NM_008546	Mfap2	0.25	-1.2	0.02	-1.3	0	-1.6
A_52_P304128	NM_008608	Mmp14	0.31	-1.4	0.02	-1.5	0.02	-1.6
A_51_P108978	NM_016969	Myadm	0.11	-1.4	0	-1.6	0	-1.6
A_51_P151433	NM_026554	Ncbp2	0.3	-1.2	0	-1.5	0	-1.6
A_55_P2091350	AK140187	ND4L	0.3	-1.4	0.01	-1.6	0.01	-1.6
A_55_P2171897	NM_008714	Notch1	0.57	-1.3	0.01	-1.5	0.01	-1.6
A_51_P114693	NM_145562	Parm1	0.93	-1.1	0.17	-1.3	0.03	-1.6
A_55_P2158522	NM_183355	Pbx1	0.14	-1.3	0	-1.5	0	-1.6
A_55_P2035717	NM_145583	Pgap2	0.63	-1.3	0.49	-1.2	0.03	-1.6

A_55_P2052623	NM_145583	Pgap2	0.64	-1.3	0.38	-1.3	0.01	-1.6
A_55_P2413598	NM_001080813	Rab11fip1	0.5	-1.3	0	-1.6	0	-1.6
A_55_P2172934	NM_029879	Rgs7bp	0.91	-1.1	0.04	-1.5	0.01	-1.6
A_55_P1993858	NR_001460	Rmrp	0.76	-1.2	0.03	-1.5	0.01	-1.6
A_55_P2041121	NR_002841	Rn4.5s	0.34	-1.4	0.01	-1.6	0	-1.6
A_51_P320444	NM_009072	Rock2	0.27	-1.3	0	-1.5	0	-1.6
A_52_P532227	NM_007901	S1pr1	0.14	-1.3	0.01	-1.4	0	-1.6
A_55_P1968763	NM_199022	Shc4	0.87	-1.1	0.04	-1.4	0.01	-1.6
A_52_P393306	NM_009222	Snap23	0.39	-1.2	0	-1.5	0	-1.6
A_51_P144500	NM_016667	Sntb1	0.48	-1.3	0.01	-1.6	0.01	-1.6
A_55_P2183498	NM_025668	Spcs2	0.15	-1.3	0	-1.6	0	-1.6
A_66_P134690	NM_146043	Spin1	0.57	-1.2	0	-1.6	0	-1.6
A_52_P291971	NM_025303	Stau2	0.47	-1.2	0.02	-1.3	0	-1.6
A_55_P1958245	NM_011552	Tcof1	0.59	-1.2	0.01	-1.5	0	-1.6
A_51_P240693	NM_027410	Tecpr1	0	-1.4	0	-1.6	0	-1.6
A_55_P2017636	NM_011580	Thbs1	0.97	-1	0.21	-1.3	0.04	-1.6
A_55_P1956978	NM_011602	Tln1	0.03	-1.4	0	-1.6	0	-1.6
A_51_P143805	NM_025339	Tmem42	0.03	-1.3	0	-1.5	0	-1.6
A_55_P2121618	NM_023434	Tox4	0.16	-1.4	0	-1.6	0	-1.6
A_55_P2001583	NM_183180	Tspan18	0.02	-1.4	0	-1.5	0	-1.6
A_55_P1985950	NM_133213	Xpnpep2	0.95	-1.1	0.93	-1	0.01	-1.6
A_55_P2127174	NM_170759	Zfp628	0.09	-1.4	0	-1.6	0	-1.6
A_51_P108020	NM_001029929	Zmynd15	0.09	-1.5	0.03	-1.4	0	-1.6
A_55_P2146483	NM_027230	Zmynd8	0.2	-1.3	0	-1.4	0	-1.6
A_30_P01030421			0.03	-1.8	0	-1.9	0.01	-1.7
A_55_P2074080			0.48	-1.2	0.01	-1.5	0	-1.7
A_30_P01020465			0.5	-1.2	0.01	-1.5	0	-1.7
A_55_P2028511			0.03	-1.4	0	-1.5	0	-1.7
A_30_P01022868			0.28	-1.3	0	-1.5	0	-1.7
A_66_P123735	AK143547		0.24	-1.3	0	-1.5	0	-1.7
A_30_P01031694			0.57	-1.4	0.03	-1.6	0.04	-1.7
A_30_P01031525			0.08	-1.7	0.02	-1.6	0.01	-1.7
A_55_P1968085			0.51	-1.3	0.02	-1.6	0.01	-1.7
A_30_P01028277			0.16	-1.3	0	-1.6	0	-1.7
A_55_P2072315	AK133410		0.04	-1.4	0	-1.6	0	-1.7
A_55_P2183668			0.05	-1.4	0	-1.6	0	-1.7
A_30_P01022751			0.02	-1.4	0	-1.6	0	-1.7
A_30_P01020570			0	-1.4	0	-1.6	0	-1.7
A_55_P2116149			0.57	-1.3	0.01	-1.7	0.01	-1.7
A_30_P01032670			0.04	-1.4	0	-1.7	0	-1.7
A_30_P01025522			0.24	-1.6	0.06	-1.6	0.04	-1.7
A_55_P2077671			0.77	-1.2	0.1	-1.4	0.01	-1.7
A_30_P01022715			0.17	-1.3	0.03	-1.3	0	-1.7
A_30_P01020852			0.2	-1.3	0.04	-1.3	0	-1.7
A_55_P2004119			0.67	-1.2	0.02	-1.4	0	-1.7
A_55_P2069787			0.5	-1.2	0.01	-1.4	0	-1.7
A_30_P01024606			0.89	-1.1	0.05	-1.4	0	-1.7
A_30_P01031585			0.11	-1.4	0.02	-1.4	0	-1.7
A_55_P1954724	NR_002860	A130040M12Rik	0.11	-1.5	0	-1.6	0	-1.7
A_55_P1974522	NM_175688	A530099J19Rik	0	-1.6	0	-1.7	0	-1.7
A_55_P2038358	NM_012006	Acot1	0.85	-1.1	0.01	-1.5	0	-1.7
A_55_P2018176	AK039146	AI504432	0.11	-1.3	0	-1.6	0	-1.7
A_55_P2174743	NM_018747	Akap7	0.61	-1.3	0.03	-1.5	0.01	-1.7
A_51_P419389	NM_007561	Bmpr2	0.64	-1.2	0	-1.5	0	-1.7
A_51_P509573	NM_013652	Ccl4	0.37	-1.4	0.5	-1.2	0.01	-1.7

A_55_P2009918	NM_007735	Col4a4	0.56	-1.3	0.02	-1.6	0.02	-1.7
A_51_P241995	NM_016919	Col5a3	0	-1.8	0	-1.8	0.01	-1.7
A_55_P1959973	NM_175539	Dcaf12l2	0.83	-1.1	0.04	-1.5	0.01	-1.7
A_55_P1961761	NM_007831	Dcc	0.63	-1.3	0.01	-1.7	0.02	-1.7
A_51_P326229	NM_013932	Ddx25	0.8	-1.2	0.07	-1.5	0.02	-1.7
A_55_P2430472	NM_011805	Dido1	0.28	-1.3	0	-1.6	0	-1.7
A_55_P1991911	XM_909743	EG626095	0.57	-1.2	0.01	-1.5	0	-1.7
A_55_P2017845	NM_153078	Ehbp1	0.49	-1.2	0.01	-1.4	0	-1.7
A_55_P2399718	AK086046	ENSMUSG00000065996	0.24	-1.3	0	-1.5	0	-1.7
A_55_P2029902	NM_181584	Gab3	0.36	-1.3	0.01	-1.7	0	-1.7
A_55_P2114697	NM_008103	Gcm1	0.54	-1.3	0.01	-1.7	0.01	-1.7
A_55_P1999818	XM_001472026	Gm2006	0.14	-1.4	0	-1.6	0	-1.7
A_55_P2092831	XM_001472097	Gm2008	0.58	-1.3	0.01	-1.7	0.01	-1.7
A_55_P2148400	XM_001472970	Gm2264	0.86	-1.1	0.07	-1.5	0.01	-1.7
A_55_P2021398	XM_001473399	Gm2393	0.56	-1.3	0.01	-1.7	0.02	-1.7
A_55_P2035038	XM_001473590	Gm2437	0.64	-1.3	0	-1.7	0.01	-1.7
A_55_P1980621	XM_001473665	Gm2459	0.57	-1.2	0.02	-1.5	0	-1.7
A_55_P2068247	XM_001473755	Gm2488	0.65	-1.3	0.03	-1.6	0.02	-1.7
A_55_P2096127	XM_001473875	Gm2527	0.45	-1.3	0.01	-1.6	0	-1.7
A_55_P2171788	XM_001474084	Gm2598	0.45	-1.3	0.01	-1.7	0	-1.7
A_55_P2082841	XM_001474255	Gm2658	0.42	-1.3	0	-1.6	0	-1.7
A_55_P1962602	XM_001474429	Gm2690	0.84	-1.2	0.21	-1.4	0.02	-1.7
A_55_P2133624	XM_001475193	Gm2891	0.1	-1.4	0	-1.5	0	-1.7
A_55_P2109922	XM_001474867	Gm2921	0.37	-1.3	0.08	-1.3	0	-1.7
A_55_P2075731	XM_001475177	Gm2943	0.43	-1.3	0	-1.7	0	-1.7
A_66_P113505	XM_001475977	Gm3006	0.21	-1.3	0	-1.5	0	-1.7
A_55_P1960479	XM_001475709	Gm3114	0.51	-1.3	0.01	-1.7	0.01	-1.7
A_55_P2070766	XM_001476070	Gm3241	0.58	-1.3	0.01	-1.6	0.01	-1.7
A_55_P2019833	XM_001477941	Gm3790	0.64	-1.3	0.02	-1.7	0.02	-1.7
A_66_P140533	XM_001478088	Gm3813	0.16	-1.3	0	-1.6	0	-1.7
A_55_P1989765	XM_001478600	Gm3951	0.49	-1.3	0.02	-1.5	0.01	-1.7
A_55_P1957213	XM_001479389	Gm4148	0.13	-1.5	0.01	-1.5	0	-1.7
A_55_P2086954	XM_001479991	Gm4372	0.53	-1.3	0.01	-1.7	0	-1.7
A_55_P2108275	XM_001480646	Gm4578	0.63	-1.3	0.02	-1.7	0.02	-1.7
A_55_P1979067	XM_001481217	Gm4720	0.7	-1.2	0.01	-1.5	0	-1.7
A_65_P15809	NR_003967	Gm4759	0.84	-1.1	0.01	-1.4	0	-1.7
A_55_P2010586	AK132285	Gm6252	0.47	-1.2	0	-1.5	0	-1.7
A_55_P2028847	XM_001476302	Gm9468	0.66	-1.3	0.02	-1.7	0.02	-1.7
A_52_P554703	NM_183183	Gprin3	0.62	-1.2	0.01	-1.5	0	-1.7
A_51_P155323	NM_010406	Hc	0.76	-1.2	0.04	-1.4	0	-1.7
A_55_P2025153	NM_010439	Hmgb1	0.7	-1.2	0.02	-1.5	0	-1.7
A_52_P141161	NM_133834	Hnrnpf	0.25	-1.3	0	-1.5	0	-1.7
A_55_P2151638	NM_013793	Klra15	0.5	-1.3	0.42	-1.2	0	-1.7
A_55_P2138739	XM_001473119	LOC100039570	0.44	-1.4	0.01	-1.8	0.01	-1.7
A_55_P2179246	XM_001472564	LOC100044553	0.48	-1.4	0.02	-1.6	0.02	-1.7
A_55_P2183518	XM_001473350	LOC100044930	0.28	-1.4	0	-1.7	0.01	-1.7
A_55_P2020035	XM_001473697	LOC100045113	0.52	-1.3	0.01	-1.7	0.01	-1.7
A_55_P2027337	XM_001473753	LOC100045145	0	-1.4	0	-1.6	0	-1.7
A_55_P1962699	XM_001477233	LOC100047021	0.5	-1.3	0	-1.6	0	-1.7
A_55_P2014326	XM_001478106	LOC100047416	0.8	-1.2	0.03	-1.5	0	-1.7
A_55_P1982227	XM_001480822	LOC100048656	0.61	-1.2	0.01	-1.6	0.01	-1.7
A_55_P1988260	XM_001479382	LOC100048847	0.05	-1.3	0	-1.5	0	-1.7
A_55_P2129407	XM_908705	LOC634091	0.42	-1.4	0.03	-1.6	0.01	-1.7
A_55_P2018307	XM_001005025	LOC677576	0.04	-1.4	0	-1.6	0	-1.7
A_55_P1977776	NR_003280	LOC790956	0.99	1	0.49	-1.3	0.04	-1.7

A_52_P212686	NM_146191	Lrrk1	0.16	-1.3	0	-1.6	0	-1.7
A_51_P150521	NM_146035	Mgat2	0.39	-1.3	0.02	-1.4	0	-1.7
A_55_P2003541	NM_176930	Nrcam	0.24	-1.4	0.05	-1.4	0	-1.7
A_52_P565940	NM_008739	Nsd1	0.08	-1.4	0	-1.5	0	-1.7
A_52_P667913	NM_033592	Pcdhga9	0.38	-1.4	0.02	-1.5	0.01	-1.7
A_55_P2183672	NM_018858	Pebp1	0.09	-1.9	0.04	-1.7	0.05	-1.7
A_55_P1960999	NM_011082	Pigr	0.57	-1.3	0.01	-1.6	0	-1.7
A_55_P2040168	NM_008885	Pmp22	0.62	-1.2	0.02	-1.4	0	-1.7
A_55_P2009449	NM_175498	Pnma2	0.53	-1.3	0.02	-1.6	0.01	-1.7
A_55_P1963491	NM_008972	Ptma	0.07	-1.3	0	-1.5	0	-1.7
A_55_P1981829	NM_001004193	Rhox8	0.61	-1.3	0.02	-1.7	0.02	-1.7
A_55_P2144436	NM_010925	Rrp1	0.34	-1.2	0	-1.5	0	-1.7
A_52_P134023	NM_153100	Rtp3	0.25	-1.4	0	-1.8	0.01	-1.7
A_51_P419226	NM_025393	S100a14	0.2	-1.5	0.02	-1.6	0	-1.7
A_52_P273169	NM_001082414	Sh3d19	0.02	-1.4	0	-1.5	0	-1.7
A_51_P318830	NM_018803	Syt10	0.19	-1.4	0	-1.7	0	-1.7
A_55_P2030160	NM_001142920	Tcf7l2	0.02	-1.4	0	-1.5	0	-1.7
A_55_P2413458	NM_023755	Tcfcp2l1	0.57	-1.2	0	-1.5	0	-1.7
A_55_P1989865	NM_146153	Thrap3	0.16	-1.3	0	-1.6	0	-1.7
A_55_P2123716	NR_002321	Tug1	0.56	-1.2	0	-1.5	0	-1.7
A_55_P1964559	NR_002888	Vmn2r-ps14	0.06	-1.3	0	-1.5	0	-1.7
A_30_P01019885			0.04	-1.5	0	-1.8	0	-1.8
A_52_P311031	AK016943		0.04	-1.5	0	-1.8	0	-1.8
A_30_P01020935			0	-1.6	0	-2	0	-1.8
A_55_P2083426			0.6	-1.3	0.02	-1.5	0	-1.8
A_55_P2131481			0.59	-1.2	0.01	-1.5	0	-1.8
A_52_P163515			0.1	-1.3	0	-1.5	0	-1.8
A_55_P2124110	AK004107		0.22	-1.3	0	-1.5	0	-1.8
A_30_P01022343			0.1	-1.4	0	-1.6	0	-1.8
A_30_P01025536			0.05	-1.4	0	-1.7	0	-1.8
A_30_P01025354			0.04	-1.4	0	-1.7	0	-1.8
A_30_P01020476			0.07	-1.4	0	-1.7	0	-1.8
A_55_P2076805	AK129022		0.08	-1.5	0	-1.7	0	-1.8
A_30_P01024297			0.03	-1.4	0	-1.8	0	-1.8
A_30_P01026230			0	-1.4	0	-1.8	0	-1.8
A_55_P2064171			0.41	-1.4	0	-1.8	0	-1.8
A_30_P01020677			0.73	-1.3	0.01	-1.9	0.03	-1.8
A_55_P1953377			0.46	-1.4	0.11	-1.5	0.01	-1.8
A_55_P2029746	XM_001472371	1200016E24Rik	0.07	-1.5	0	-1.8	0	-1.8
A_55_P2105321	NM_023190	Acin1	0.34	-1.3	0	-1.7	0	-1.8
A_52_P162509	NM_021414	Ahcyl2	0.1	-1.5	0	-1.9	0	-1.8
A_52_P625171	NM_133723	Asph	0.16	-1.3	0	-1.6	0	-1.8
A_55_P2017769	NM_001033769	B020031M17Rik	0.68	-1.3	0.07	-1.6	0.03	-1.8
A_51_P413866	NM_008198	Cfb	0.21	-1.7	0.56	-1.2	0.03	-1.8
A_51_P515605	NM_009930	Col3a1	0.07	-1.7	0	-1.9	0	-1.8
A_51_P116813	NM_007809	Cyp17a1	0.11	-1.4	0	-1.6	0	-1.8
A_51_P334104	NM_007833	Dcn	0.5	-1.4	0	-1.9	0.01	-1.8
A_55_P2087984	NM_001164671	Dnaja1	0.8	-1.2	0.06	-1.7	0.03	-1.8
A_55_P2014978	NM_007889	Dvl3	0.04	-1.5	0	-1.8	0	-1.8
A_55_P2089710	NM_007904	Ednrb	0.97	1	0.18	-1.3	0	-1.8
A_51_P388478	NM_010110	Efnb1	0	-1.5	0	-1.8	0	-1.8
A_55_P2136752	NM_029972	Ermn	0.48	-1.3	0	-1.7	0	-1.8
A_51_P267544	NM_013522	Frg1	0.33	-1.3	0	-1.5	0	-1.8
A_55_P2154107	NM_008103	Gcm1	0.55	-1.3	0.01	-1.7	0.01	-1.8
A_55_P2039196	NM_020014	Gfra4	0.06	-1.4	0	-1.6	0	-1.8

A_55_P2143516	XM_001473058	Gm2291	0.2	-1.4	0	-1.8	0	-1.8
A_55_P2068248	XM_001473755	Gm2488	0.41	-1.3	0	-1.7	0	-1.8
A_55_P2140212	XM_001474216	Gm2627	0.24	-1.5	0	-1.8	0	-1.8
A_66_P125110	XM_001475977	Gm3006	0.31	-1.3	0	-1.6	0	-1.8
A_55_P2042184	XM_001476516	Gm3181	0.78	-1.2	0.02	-1.4	0	-1.8
A_55_P2041457	XM_001475948	Gm3195	0.39	-1.3	0.01	-1.7	0	-1.8
A_55_P1974780	XM_001476301	Gm3306	0.47	-1.4	0.01	-1.7	0.01	-1.8
A_55_P2063505	XM_001479508	Gm4235	0.64	-1.3	0.02	-1.7	0	-1.8
A_55_P2144364	XM_001479624	Gm4320	0.56	-1.3	0.04	-1.4	0	-1.8
A_55_P2120141	XM_001480410	Gm4522	0.54	-1.3	0	-1.7	0	-1.8
A_55_P2077501	XM_901095	Gm6940	0.39	-1.3	0	-1.7	0	-1.8
A_55_P2071581	XM_978127	Gm7710	0.19	-1.6	0.05	-1.6	0.01	-1.8
A_51_P356055	NM_175012	Grp	0.05	-1.6	0	-1.6	0	-1.8
A_55_P2135064	NM_008204	H2-M2	0.42	-1.3	0	-1.7	0	-1.8
A_55_P1982451	NM_016710	Hmgn5	0.74	-1.2	0.07	-1.3	0	-1.8
A_52_P518922	NM_001033228	Itga1	0.31	-1.3	0	-1.7	0	-1.8
A_51_P179258	NM_001161665	Kif26b	0.92	-1.1	0.01	-1.5	0	-1.8
A_55_P2148935	XM_001474432	LOC100040286	0.36	-1.4	0	-1.7	0	-1.8
A_55_P1981830	XM_001475952	LOC100041203	0.99	-1	0.02	-1.8	0.01	-1.8
A_55_P2139430	XM_001479756	LOC100048207	0.49	-1.3	0	-1.8	0	-1.8
A_55_P2151082	XM_919699	LOC632154	0.06	-1.4	0	-1.6	0	-1.8
A_55_P2129469	XM_918544	LOC641199	0.32	-1.4	0.23	-1.3	0	-1.8
A_55_P2175752	NM_001081975	Mfap1b	0.8	-1.2	0.02	-1.5	0	-1.8
A_55_P2454099	NM_023799	Mgea5	0.21	-1.3	0	-1.6	0	-1.8
A_55_P2035320	NM_017373	Nfil3	0	-1.7	0	-1.8	0	-1.8
A_55_P2104917	NM_016902	Nphp1	0.37	-1.3	0	-1.5	0	-1.8
A_55_P2171623	NM_001077363	Ptbp1	0.06	-1.3	0	-1.7	0	-1.8
A_55_P2044627	NM_029145	Rnase10	0.24	-1.3	0.01	-1.5	0	-1.8
A_52_P140881	NM_177615	Slc26a10	0.07	-1.5	0	-1.9	0	-1.8
A_55_P1965030	NM_001003915	Slc5a12	0.65	-1.2	0.01	-1.4	0	-1.8
A_52_P467690	NM_175836	Spnb2	0.45	-1.3	0	-1.7	0	-1.8
A_55_P2000973	NM_181529	Syt15	0.45	-1.3	0.04	-1.4	0	-1.8
A_66_P100937	XM_001487796	Zfp33b	0	-1.4	0	-1.5	0	-1.8
A_30_P01032068			0.02	-1.5	0	-1.8	0	-1.9
A_30_P01032942			0	-1.5	0	-1.8	0	-1.9
A_55_P1982075			0.56	-1.3	0	-1.7	0	-1.9
A_55_P2093862	AK156257		0.55	-1.3	0.05	-1.4	0	-1.9
A_55_P2073935	NM_013790	Abcc5	0.06	-1.5	0	-1.8	0	-1.9
A_52_P650387	NM_001045530	Ccnjl	0.02	-1.6	0	-2	0	-1.9
A_55_P2158404	NM_020557	Cmpk2	0.46	-1.5	0.31	-1.4	0.03	-1.9
A_55_P2143837	NM_007735	Col4a4	0.33	-1.4	0	-1.8	0	-1.9
A_52_P577662	NM_007904	Ednrb	1	-1	0.03	-1.6	0	-1.9
A_51_P296249	NM_010226	Foxs1	0.11	-1.5	0.01	-1.7	0	-1.9
A_55_P2088720	XM_001477698	Gm3651	0.1	-1.5	0	-1.7	0	-1.9
A_55_P2126557	XM_619973	Gm5858	0.06	-1.5	0	-2	0	-1.9
A_51_P464822	NM_015787	Hist1h1e	0.42	-1.3	0.03	-1.4	0	-1.9
A_55_P2001494	NM_013598	Kitl	0.63	-1.2	0.02	-1.4	0	-1.9
A_52_P514407	NM_013793	Klra15	0.42	-1.4	0.24	-1.3	0	-1.9
A_55_P2151209	NM_130873	Krtap16-4	0	-1.5	0	-1.7	0	-1.9
A_51_P210143	NM_001005510	Syne2	0.12	-1.4	0	-1.8	0	-1.9
A_52_P355169	NM_011607	Tnc	0.07	-1.7	0.03	-1.6	0	-1.9
A_55_P2127243	NM_010781	Tpsb2	0.13	-1.5	0.03	-1.5	0	-1.9
A_51_P485458	NM_001005506	Txlna	0.02	-1.4	0	-1.7	0	-1.9
A_30_P01020696			0.96	-1.1	0.89	-1.1	0.03	-2
A_55_P2138100			0.49	-1.3	0.06	-1.4	0	-2

A_66_P124164	AK089567		0.51	-1.3	0.05	-1.4	0	-2
A_55_P1978636			0.67	-1.2	0.11	-1.4	0	-2
A_52_P1020860	XM_888885	AW112010	0.48	-1.5	0.42	-1.3	0.01	-2
A_55_P2061620	NM_023850	Chst1	0.92	-1.1	0.07	-1.5	0	-2
A_51_P146560	NM_018857	Msln	0.3	-2	0.01	-2.5	0.1	-2
A_55_P2165790	NM_178706	Siglech	0.12	-1.5	0	-1.8	0	-2
A_52_P799815	NM_001025606	Tmem171	0	-1.7	0	-2	0	-2
A_52_P45738	NM_029979	Trim35	0.45	-1.3	0.01	-1.8	0	-2
A_55_P2165554			0.68	-1.3	0.11	-1.4	0	-2.1
A_51_P265571	NM_009627	Adm	0.17	-1.6	0.03	-1.6	0	-2.1
A_55_P2403769	BB498095	AI481121	0.77	-1.2	0.01	-1.6	0	-2.1
A_66_P105032	NM_001145034	Gm13889	0.02	-1.6	0.01	-1.5	0	-2.1
A_52_P409833	NM_008872	Plat	0.05	-1.6	0	-1.8	0	-2.1
A_55_P2138104	AK089567		0.41	-1.4	0.03	-1.5	0	-2.2
A_55_P2153620	NM_001039959	Ahnak	0.11	-1.5	0	-2	0	-2.2
A_51_P338443	NM_020581	Angptl4	0.57	-1.3	0.05	-1.5	0	-2.2
A_55_P2175469	NM_001081345	Chd2	0.23	-1.4	0	-1.8	0	-2.2
A_66_P106774	NM_029990	Lhfpl3	0.02	-1.7	0	-1.8	0	-2.2
A_55_P2116650	NR_002860	A130040M12Rik	0.04	-1.6	0	-2.1	0	-2.3
A_55_P1960738	XM_001472780	Gm4470	0.09	-1.5	0	-1.6	0	-2.3
A_51_P331328	NM_026730	Gpihbp1	0.67	-1.2	0.01	-1.5	0	-2.3
A_55_P2028734	NM_013794	Klra16	0.14	-1.7	0.07	-1.5	0	-2.3
A_55_P2062793	NM_008546	Mfap2	0	-1.5	0	-1.8	0	-2.3
A_55_P2141479	NR_004414	Rnu2	0.09	-1.6	0	-2.2	0	-2.3
A_52_P229052	NM_019790	Tmeff2	0.8	-1.3	0.08	-1.6	0	-2.3
A_55_P2465382	AK172117		0.19	-1.5	0.01	-1.7	0	-2.5
A_55_P2160416	NM_028765	Acoxl	0.92	1.2	0.92	1.1	0	-2.5
A_55_P2170349	NM_053152	Klra22	0.17	-1.7	0.16	-1.5	0	-2.5
A_55_P2004536	NM_010649	Klra4	0.16	-1.6	0.02	-1.6	0	-2.5
A_52_P638459	NM_013653	Ccl5	0	-1.9	0	-1.8	0	-2.8
A_55_P2408588	NM_007489	Arntl	0	-2.1	0	-2.6	0	-3.1
A_55_P2085288	NM_028765	Acoxl	0.98	1.1	0.81	1.2	0	-3.4
A_51_P175424	NM_011797	Car14	0	-2.3	0	-3.1	0	-3.4
A_52_P381484	NM_133903	Spon2	0	-2.3	0	-2.8	0	-3.5
A_55_P2068459	NM_010479	Hspa1a	0.34	-2	0	-3.3	0	-4
A_55_P1990032	NM_009141	Cxcl5	0	-3.7	0.01	-3.2	0	-4.4
A_55_P1963483	NM_030559	Vps16	0.89	1.3	0.75	1.3	0	-4.6
A_55_P2094060	NM_010370	Gzma	0.11	-2.3	0.07	-2	0	-4.8
A_55_P2011341	XM_001474162	LOC100045268	0.13	-2.2	0.06	-1.9	0	-5.1

2.2 8PAH-Mix. Significant probe list. List of all significantly differentially expressed probes in at least one treatment group (FDR P ≤ 0.05, fold change ± 1.5) in response to sub-chronic oral exposure to 15, 30, and 60 mg BaP equivalents /kg-bw/day of a mixture of 8 PAHs in the lungs. The list is sorted from highest to lowest fold change in the 60 mg BaP equivalents/kg-bw/day treatment group.

			Low dose 15 mg BaP equivalents/ kg-day		Medium dose 30 mg BaP equivalents/ kg-day		High dose 60 mg BaP equivalents/ kg-day	
Agilent Probe	Accession Number	Gene Symbol	*FDR P value*	*Fold change*	*FDR P value*	*Fold change*	*FDR P value*	*Fold change*

A_55_P1960735	NM_011819	Gdf15	0	6.2	0	6.9	0	13.4
A_51_P279693	NM_009992	Cyp1a1	0.06	2.5	0	8	0	9.3
A_51_P363947	NM_007669	Cdkn1a	0	3.6	0	4	0	8.7
A_55_P1986282	NM_001111099	Cdkn1a	0	4.2	0	4.3	0	8.2
A_55_P1959500	NM_172759	Ces5	0	4	0	5.6	0	7.4
A_51_P255456	NM_009994	Cyp1b1	0	3.2	0	5.7	0	6.1
A_51_P329928	NM_013750	Phlda3	0	2.5	0	3	0	5.1
A_51_P414396	NM_153127	Mmrn2	0	2	0	2.6	0	4.8
A_55_P1972948	NM_176954	Brunol5	0	1.6	0	2.5	0	4.7
A_52_P612803	NM_009831	Ccng1	0	2.2	0	2.4	0	4.7
A_55_P2101340	NM_019511	Ramp3	0	2.1	0	2.8	0	4
A_55_P2031999	NM_145448	9030617O03Rik	0	1.9	0	2.7	0	3.9
A_55_P1954835	XM_001475752	LOC100046186	0	1.9	0	2.7	0	3.6
A_55_P2032081	NM_016974	Dbp	0	2.8	0	4	0	3.5
A_55_P2032079	NM_016974	Dbp	0	2.8	0	4.3	0	3.5
A_55_P1953169	NM_011315	Saa3	0.38	1.5	0.43	1.6	0	3.5
A_55_P1963017	NM_001082543	Stfa1	0.19	1.4	0.06	1.6	0	3.3
A_51_P254425	NM_009644	Ahrr	0	2.6	0	3.4	0	3.2
A_55_P2145804	NM_026531	Aen	0	1.7	0	2.2	0	3.1
A_52_P675395	NM_007722	Cxcr7	0	1.7	0.08	1.5	0	3.1
A_30_P01026536			0	1.8	0	1.7	0	3
A_55_P2143572	AK020725		0	1.7	0	2.2	0	3
A_30_P01025511			0.01	1.5	0.01	1.6	0	3
A_51_P256827	NM_013650	S100a8	0.04	1.9	0.02	2.2	0	3
A_51_P323620	NM_144543	Thyn1	0	1.7	0	2	0	3
A_30_P01020960			0	1.7	0	2.1	0	2.9
A_55_P2005213	NM_145603	Ces2	0	2.1	0	2.1	0	2.9
A_55_P2002578	NM_010145	Ephx1	0	1.8	0	2.2	0	2.9
A_52_P539310	NM_001160326	Serp2	0	2	0	2.2	0	2.9
A_55_P2119257	NM_008871	Serpine1	0	1.7	0	2.1	0	2.9
A_51_P269203	NM_022017	Trpv4	0	2.3	0.02	1.8	0	2.9
A_55_P2141860	NM_026531	Aen	0	1.6	0	2.1	0	2.8
A_52_P700056	NM_001081957	Gm11428	0.45	1.3	0.93	1.1	0	2.8
A_51_P329332	NM_054087	Slc19a2	0.01	1.6	0.01	1.8	0	2.8
A_55_P2060922	NM_153131	Unc5a	0.39	1.8	0.93	-1.2	0.03	2.8
A_51_P487073	NM_138684	Wfdc12	0.03	1.7	0.02	1.8	0	2.8
A_51_P415220	NM_009517	Zmat3	0.06	1.4	0	1.8	0	2.8
A_30_P01027010			0	1.7	0	2.1	0	2.7
A_30_P01024344			0	1.4	0	1.6	0	2.7
A_55_P2137049	NM_001004174	AA467197	0.87	1.1	0.74	1.3	0	2.7
A_51_P110471	NM_026993	Ddah1	0	2	0	1.9	0	2.7
A_55_P2143025	NM_013657	Sema3c	0.1	1.4	0	2	0	2.7
A_51_P175580	NM_021897	Trp53inp1	0	1.8	0	2.3	0	2.7
A_30_P01032002			0.01	1.4	0	1.5	0	2.6
A_52_P487686	NM_001082546	BC100530	0.19	1.3	0.26	1.4	0	2.6
A_51_P185660	NM_011338	Ccl9	0.16	1.4	0.07	1.6	0	2.6
A_51_P307168	NM_026993	Ddah1	0	1.9	0	2.1	0	2.6
A_52_P627068	NM_170593	Disp2	0.64	1.2	0.14	1.7	0	2.6
A_51_P405397	NM_007899	Ecm1	0.02	1.5	0	1.8	0	2.6
A_55_P2002577	NM_010145	Ephx1	0	1.9	0	1.8	0	2.6
A_66_P118600	NM_008480	Lama1	0	2	0	2.3	0	2.6
A_51_P231320	NM_008611	Mmp8	0.01	1.9	0.02	2	0	2.6
A_66_P115580	AK076360		0	2.1	0	2.5	0	2.5
A_52_P311853	NM_030143	Ddit4l	0	1.5	0	1.6	0	2.5
A_51_P449824	XM_001471750	Exoc3l2	0	1.6	0	1.7	0	2.5

A_55_P2039320	NM_178679	Zfp365	0.15	1.3	0.01	1.6	0	2.5
A_52_P40504	AK162948		0	1.7	0	1.6	0	2.4
A_51_P383032	NM_010819	Clec4d	0.06	1.5	0.1	1.6	0	2.4
A_52_P686785	NM_053247	Lyve1	0.01	2.1	0.08	1.9	0	2.4
A_55_P2090359	NM_148937	Plcd4	0.53	1.2	0.26	1.5	0	2.4
A_55_P2011146	NM_178608	Reep1	0.03	1.5	0	1.8	0	2.4
A_55_P1965154	NM_025565	Spc25	0	3.4	0	2.7	0	2.4
A_55_P2094925	NM_011157	Srgn	0	1.7	0	1.8	0	2.4
A_55_P2007713	NM_053082	Tspan4	0	1.8	0	2.1	0	2.4
A_30_P01025790			0	1.6	0	1.8	0	2.3
A_52_P151393	NM_198860	AI646023	0	1.5	0	1.7	0	2.3
A_55_P2110758	NM_177083	B430306N03Rik	0.09	1.5	0.04	1.6	0	2.3
A_51_P204740	NM_133654	Cd34	0.42	1.2	0.17	1.4	0	2.3
A_51_P350403	NM_009148	Exoc4	0.01	1.5	0.03	1.5	0	2.3
A_52_P398925	NM_173869	Stfa2l1	0.44	1.3	0.33	1.5	0	2.3
A_52_P534583	NM_133245	Ahsp	0.22	1.4	0.02	1.9	0	2.2
A_51_P372550	NM_026770	Cgref1	0.22	1.2	0.11	1.3	0	2.2
A_66_P104815	NM_007899	Ecm1	0.01	1.6	0.08	1.5	0	2.2
A_51_P516133	NM_015786	Hist1h1c	0.19	1.3	0.06	1.5	0	2.2
A_52_P327588	NM_148937	Plcd4	0.25	1.3	0.5	1.3	0	2.2
A_51_P290576	NM_152804	Plk2	0	1.6	0.01	1.5	0	2.2
A_55_P2429225	NM_019976	Psrc1	0.15	1.3	0.28	1.3	0	2.2
A_52_P425839	NM_181596	Retnlg	0.38	1.4	0.49	1.4	0	2.2
A_51_P246903	NM_026467	Rps27l	0	1.4	0	1.5	0	2.2
A_55_P1960238	NM_172659	Slc2a6	0.29	1.3	0.03	1.8	0	2.2
A_55_P2097518	NM_001080943	Zdhhc22	1	-1	0.94	1.1	0.02	2.2
A_55_P2088145			0.01	1.6	0.17	1.4	0	2.1
A_30_P01026167			0.05	1.4	0	1.6	0	2.1
A_30_P01029956			0	1.4	0	1.5	0	2.1
A_30_P01026923			0.19	1.5	0.39	1.4	0	2.1
A_52_P29953	NM_175398	6530418L21Rik	0	1.6	0	1.6	0	2.1
A_55_P2136121	NM_145448	9030617O03Rik	0	1.5	0	1.8	0	2.1
A_55_P2137406	NM_007527	Bax	0.16	1.4	0	2.4	0	2.1
A_55_P2405784	AK141429	BC023202	0.17	1.3	0.03	1.4	0	2.1
A_51_P401907	NM_001082547	Gm5483	0.43	1.3	0.33	1.4	0	2.1
A_55_P2056729	NM_008342	Igfbp2	0.77	1.2	0.3	1.5	0	2.1
A_66_P135391	NM_008342	Igfbp2	0.71	1.2	0.15	1.5	0	2.1
A_55_P2039250	NM_026037	Mboat2	0.1	1.4	0.14	1.5	0	2.1
A_51_P270184	NM_025811	Nhlrc2	0.01	1.5	0	2	0	2.1
A_55_P2095271	NM_153805	Pkn3	0.01	1.6	0.05	1.5	0	2.1
A_55_P2137421	NM_022017	Trpv4	0	1.8	0.15	1.4	0	2.1
A_66_P136186	NM_009516	Wee1	0.02	1.5	0	2	0	2.1
A_30_P01019901			0.01	1.7	0.07	1.5	0	2
A_51_P367780	NM_029981	Adamtsl2	0.13	1.5	0.43	1.4	0.01	2
A_55_P2082688	NM_021515	Ak1	0.42	1.2	0.55	1.2	0	2
A_66_P111562	NM_007631	Ccnd1	0.03	1.3	0	1.5	0	2
A_51_P498631	NM_018769	Dfna5	0.04	1.3	0.02	1.5	0	2
A_51_P480328	NM_133222	Eltd1	0.01	1.6	0.4	1.3	0	2
A_51_P507801	NM_028784	F13a1	0.24	1.3	0	1.8	0	2
A_55_P2042016	XM_001479435	LOC100048058	0	1.4	0	1.6	0	2
A_52_P111031	NM_001013753	Pcdh17	0.09	1.4	0.01	1.9	0	2
A_55_P2027022	NM_007548	Prdm1	0.02	1.4	0	1.7	0	2
A_55_P2168628	NM_133678	Sac3d1	0	1.5	0.01	1.4	0	2
A_55_P2048119	NM_146257	Slc29a4	0.05	1.4	0.04	1.6	0	2
A_55_P2008936	NM_001102414	Slc2a9	0.09	1.3	0.01	1.4	0	2

A_55_P1973906	NM_021897	Trp53inp1	0.02	1.4	0	1.6	0	2
A_30_P01025143			0.01	1.4	0	1.6	0	1.9
A_66_P138053	AK044848		0.1	1.3	0.09	1.4	0	1.9
A_55_P1976204	U09507		0.02	1.4	0.04	1.4	0	1.9
A_51_P108226	NM_183249	1100001G20Rik	0.37	1.2	0.65	1.2	0	1.9
A_51_P113178	NM_175398	6530418L21Rik	0.11	1.3	0.11	1.4	0	1.9
A_55_P2169227	NM_177716	AI836003	0.04	1.6	0.19	1.5	0	1.9
A_51_P499698	NM_026414	Asprv1	0.55	1.2	0.34	1.4	0	1.9
A_51_P413785	NM_147778	Commd3	0	1.4	0.01	1.4	0	1.9
A_51_P300506	NM_183405	Cox6b2	0.42	1.3	0.74	1.2	0	1.9
A_52_P269942	NM_153679	Cpt1c	0.46	1.2	0.34	1.2	0	1.9
A_55_P2110245	NM_001163359	Fignl1	0	1.8	0.01	2	0	1.9
A_51_P212782	NM_008361	Il1b	0.37	1.3	0.48	1.3	0	1.9
A_55_P2070869	NM_008491	Lcn2	0.63	1.2	0.74	1.2	0	1.9
A_51_P371750	NM_010766	Marco	0.74	1.2	0.27	1.7	0.03	1.9
A_51_P111962	NM_001141922	mCG_21548	0.2	1.4	0.53	1.3	0	1.9
A_55_P2071858	NM_008598	Mgmt	0.45	1.2	0.07	1.5	0	1.9
A_51_P341736	NM_008610	Mmp2	0.1	1.3	0.42	1.2	0	1.9
A_52_P536494	NM_008709	Mycn	0	1.6	0.01	1.5	0	1.9
A_55_P2078365	NM_173402	Rgs12	0	1.5	0	1.6	0	1.9
A_52_P236705	NM_133229	Ripply3	0.12	1.3	0	1.6	0	1.9
A_55_P2051094	NM_011281	Rorc	0.12	1.3	0.18	1.3	0	1.9
A_55_P2003813	NM_153522	Scn3b	0.07	1.3	0.02	1.5	0	1.9
A_52_P87839	NM_013657	Sema3c	0.94	1	0.14	1.6	0	1.9
A_51_P161354	NM_144907	Sesn2	0	1.4	0	1.6	0	1.9
A_52_P220810	NM_144551	Trib2	0.01	1.5	0.01	1.8	0	1.9
A_55_P1986833	NM_054040	Tulp4	0.06	1.4	0.03	1.5	0	1.9
A_55_P2035286	NM_010931	Uhrf1	0	1.9	0	2.1	0	1.9
A_30_P01032234			0.02	1.5	0.01	1.7	0	1.8
A_30_P01018981			0.03	1.4	0.02	1.5	0	1.8
A_30_P01033517			0.58	1.3	0.51	1.4	0.02	1.8
A_30_P01026752			0.11	1.2	0.06	1.3	0	1.8
A_52_P301374	NM_153416	Aaas	0	1.5	0	1.4	0	1.8
A_51_P185906	NM_025659	Abi3	0.21	1.4	0.92	1.1	0	1.8
A_51_P382789	NM_027025	Adora3	0.38	1.3	0.28	1.4	0	1.8
A_55_P2052062	NM_010818	Cd200	0.06	1.4	0.01	1.6	0	1.8
A_52_P559975	NM_009909	Cxcr2	0.83	1.1	0.21	1.4	0	1.8
A_55_P1998001	NM_001025384	DXBay18	0.12	1.3	0.23	1.3	0	1.8
A_55_P1958887	NM_001025384	DXBay18	0.16	1.3	0.5	1.2	0	1.8
A_52_P325527	NM_010103	Edil3	0.05	1.3	0	1.6	0	1.8
A_51_P214269	NM_023580	Epha1	0.09	1.4	0.25	1.4	0	1.8
A_51_P351896	NM_133187	Fam198b	0.13	1.3	0.67	1.2	0	1.8
A_55_P2040245	NM_001039485	Fam38b	0.78	1.1	0.1	1.4	0	1.8
A_51_P179697	NM_026884	Fam57b	0	1.5	0.04	1.4	0	1.8
A_52_P108447	NM_031184	Glis2	0.04	1.3	0	1.5	0	1.8
A_51_P355753	NM_010430	Hic1	0.25	1.3	0.53	1.2	0	1.8
A_51_P180140	NM_175663	Hist1h2ba	0.06	1.5	0.25	1.4	0	1.8
A_55_P2109122	NM_023422	Hist1h2bc	0.12	1.4	0.18	1.4	0	1.8
A_55_P2021114	NM_010500	Ier5	0.01	1.4	0	1.6	0	1.8
A_51_P294555	NM_001033632	Ifitm6	0.53	1.3	0.45	1.5	0.04	1.8
A_55_P2085295	NM_198411	Inf2	0.08	1.2	0	1.5	0	1.8
A_52_P281145	NM_172872	Kank4	0.02	1.5	0.05	1.5	0	1.8
A_55_P2006261	NM_008469	Krt15	0.23	1.4	0.58	1.3	0	1.8
A_52_P130727	NM_177630	Ldoc1l	0.04	1.3	0.02	1.4	0	1.8
A_51_P354706	NM_010094	Lefty1	0	1.6	0.01	1.6	0	1.8

A_55_P2015292	NM_008521	Ltc4s	0.98	-1	0.98	1	0.01	1.8
A_55_P1975475	NM_010786	Mdm2	0.12	1.2	0.01	1.4	0	1.8
A_55_P1967291	NM_144818	Ncaph	0	1.9	0	1.9	0	1.8
A_55_P2180869	NM_029865	Ocel1	0.97	-1	0.87	1.2	0.02	1.8
A_55_P2037812	NM_023245	Palmd	0.18	1.3	0.05	1.5	0	1.8
A_51_P282760	NM_011066	Per2	0.03	1.5	0	1.8	0	1.8
A_52_P380263	NM_013723	Podxl	0.13	1.3	0.03	1.6	0	1.8
A_55_P1999561	NM_001002842	Pram1	0.06	1.4	0.1	1.4	0	1.8
A_52_P679105	NM_029614	Prss23	0.14	1.5	0.18	1.5	0.01	1.8
A_51_P148105	NM_011234	Rad51	0	1.8	0.01	1.6	0	1.8
A_55_P2173982	NM_009104	Rrm2	0	2.3	0.01	2.2	0.01	1.8
A_55_P1998471	NM_009114	S100a9	0.5	1.2	0.12	1.5	0	1.8
A_51_P299805	NM_027872	Slc46a3	0.01	1.4	0	1.8	0	1.8
A_52_P472324	NM_011414	Slpi	0.98	1	0.94	-1.1	0.03	1.8
A_55_P2121608	AK153771	Sox4	0.91	1	0.47	1.2	0	1.8
A_51_P236267	NM_009183	St8sia4	0.19	1.2	0	1.8	0	1.8
A_52_P55772	NM_009325	Tbxa2r	0.84	1.1	0.68	1.2	0	1.8
A_55_P2027836	NM_020275	Tnfrsf10b	0.87	1.1	0.54	1.2	0	1.8
A_55_P1990785	NM_027678	Zranb3	0.32	1.2	0.13	1.4	0	1.8
A_55_P1953533	AK134774		0.28	1.3	0.04	1.6	0	1.7
A_30_P01024850			0	1.3	0	1.4	0	1.7
A_30_P01022991			0.16	1.3	0.06	1.4	0	1.7
A_30_P01022528			0.05	1.3	0.02	1.4	0	1.7
A_30_P01021359			0.01	1.3	0.09	1.2	0	1.7
A_30_P01033274			0.38	1.2	0.02	1.4	0	1.7
A_30_P01018535			0.11	1.4	0.14	1.4	0	1.7
A_55_P2129316			0	1.4	0.08	1.2	0	1.7
A_51_P369252	NM_001080995	4632434I11Rik	0.33	1.2	0.5	1.2	0	1.7
A_51_P245368	NM_011075	Abcb1b	0.66	1.1	0.32	1.2	0	1.7
A_55_P2013158	NM_178688	Ablim1	0.51	1.2	0.73	1.2	0.01	1.7
A_52_P49321	NM_175314	Adamts9	0.28	1.2	0.18	1.3	0	1.7
A_55_P2090060	NM_172295	BC037703	0.8	1.1	0.98	-1	0	1.7
A_55_P2039699	NM_009921	Camp	0	2.6	0.05	2.2	0.07	1.7
A_55_P2010066	NM_007601	Capn3	0.41	1.2	0.22	1.3	0	1.7
A_51_P460954	NM_009139	Ccl6	0.62	-1.3	1	-1	0.04	1.7
A_55_P2009225	NM_133654	Cd34	0.33	1.2	0.47	1.2	0	1.7
A_55_P2021285	NM_146067	Cpped1	0	1.2	0	1.4	0	1.7
A_55_P1975415	NM_146067	Cpped1	0.15	1.2	0	1.4	0	1.7
A_55_P2032750	NM_153679	Cpt1c	0.83	1.1	0.79	1.1	0	1.7
A_55_P2018847	NM_001164735	Crlf2	0.01	1.3	0.06	1.3	0	1.7
A_51_P384629	NM_009983	Ctsd	0.89	1.1	1	1	0	1.7
A_51_P363187	NM_008176	Cxcl1	0.71	-1.1	0.72	-1.2	0	1.7
A_55_P2103249	NM_178241	Cxcr1	0.98	-1	0.87	1.2	0.04	1.7
A_51_P109258	NM_138686	Cys1	0.02	1.4	0.03	1.5	0	1.7
A_51_P170641	NM_030246	Dcaf4	0.07	1.2	0.01	1.3	0	1.7
A_52_P232637	NM_007857	Dhh	0.01	1.5	0.34	1.3	0	1.7
A_55_P2164534	NM_029766	Dtl	0	1.6	0	1.7	0	1.7
A_51_P115005	NM_010104	Edn1	0.04	1.5	0.04	1.6	0	1.7
A_55_P1988048	NM_001163522	Emcn	0.1	1.4	0.54	1.2	0	1.7
A_55_P2043108	NM_009148	Exoc4	0.23	1.2	0.1	1.4	0	1.7
A_51_P140641	NM_145570	Fam176a	0.44	1.2	0.2	1.4	0	1.7
A_55_P2130178	NM_010233	Fn1	0.8	1.1	0.5	1.3	0.01	1.7
A_51_P172054	NM_019521	Gas6	0.85	-1.1	0.72	1.2	0	1.7
A_55_P1965298	NM_001099302	Gm5640	0.16	1.3	0.65	1.2	0	1.7
A_55_P2037343	NM_010391	H2-Q10	0.17	1.4	0.29	1.4	0	1.7

A_55_P2177154	NM_178200	Hist1h2bm	0.06	1.5	0.24	1.4	0	1.7
A_55_P2009752	NM_172563	Hlf	0.19	1.4	0.01	1.9	0.01	1.7
A_55_P2031631	NM_010512	Igf1	0.68	1.2	0.65	1.2	0.01	1.7
A_55_P1981949	NM_175174	Klhl5	0.02	1.4	0	1.6	0	1.7
A_55_P1954231	NM_172492	Lrtm2	0.97	1	0.26	1.5	0.01	1.7
A_51_P487813	NM_016753	Lxn	0.49	1.2	0.17	1.3	0	1.7
A_51_P265495	NM_010738	Ly6a	0.02	1.4	0.12	1.3	0	1.7
A_55_P2064771	NM_010741	Ly6c1	0.06	1.3	0.02	1.4	0	1.7
A_55_P1987914	NM_008696	Map4k4	0.02	1.3	0	1.5	0	1.7
A_51_P426270	NM_008597	Mgp	0.21	1.3	0.5	1.3	0	1.7
A_51_P253803	NM_001081117	Mki67	0	2.8	0	2.2	0	1.7
A_52_P167278	NM_172308	Mthfd1l	0	1.4	0	1.9	0	1.7
A_55_P1953728	NM_016701	Nes	0.29	1.2	0.08	1.3	0	1.7
A_51_P515965	NM_008685	Nfe2	0.04	1.4	0.01	1.5	0	1.7
A_51_P247184	NM_008728	Npr3	0	2.5	0	2.2	0.02	1.7
A_55_P2103452	NM_010934	Npy1r	0.97	1	0.24	1.4	0	1.7
A_52_P303891	NM_011584	Nr1d2	0.03	1.4	0	1.9	0	1.7
A_52_P393314	NM_011027	P2rx7	0.01	1.4	0	1.6	0	1.7
A_55_P1980321	NM_021568	Pcbp3	0.07	1.3	0	1.5	0	1.7
A_51_P383194	NM_008804	Pde9a	0	1.7	0.06	1.4	0	1.7
A_55_P2000533	NM_012048	Polk	0.59	1.2	0.08	1.6	0	1.7
A_52_P198435	NM_207246	Rasgrp3	0.62	1.1	0.13	1.4	0	1.7
A_52_P125467	NM_144865	Reep2	0.27	1.2	0.12	1.3	0	1.7
A_55_P2083023	NM_133678	Sac3d1	0.03	1.4	0.1	1.3	0	1.7
A_51_P159453	NM_009252	Serpina3n	0.18	1.4	0.99	-1	0	1.7
A_51_P408649	NM_145463	Shisa2	0.13	1.3	0.23	1.3	0	1.7
A_51_P156438	NM_027460	Slc25a33	0.01	1.4	0	1.7	0	1.7
A_55_P1979893	NM_017376	Tef	0.04	1.4	0	2.3	0	1.7
A_51_P463765	NM_011595	Timp3	0.22	1.3	0.69	1.2	0	1.7
A_52_P8324	NM_026516	Tmem178	0.12	1.3	0.01	1.6	0	1.7
A_55_P2018017	NM_009425	Tnfsf10	0.3	1.2	0.08	1.5	0	1.7
A_51_P341918	NM_009366	Tsc22d1	0	1.3	0	1.5	0	1.7
A_55_P1957245	XM_884146	Vmn2r-ps134	0.86	1.1	0.72	1.2	0.01	1.7
A_30_P01026558			0.69	1.2	0.04	2.2	0.15	1.6
A_30_P01019396			0.37	1.2	0.05	1.5	0	1.6
A_30_P01026613			0.06	1.4	0.04	1.5	0	1.6
A_30_P01032267			0.59	1.2	0.38	1.4	0.01	1.6
A_30_P01032951			0.02	1.3	0.02	1.3	0	1.6
A_30_P01019169			0.18	1.2	0.06	1.3	0	1.6
A_30_P01021048			0.35	1.1	0.11	1.3	0	1.6
A_55_P2115136	NM_028732	4632428N05Rik	0.45	1.2	0.87	-1.1	0.02	1.6
A_52_P322181	NM_007419	Adrb1	0.05	1.4	0	1.6	0	1.6
A_55_P2002849	NM_175178	Aifm3	0.52	1.2	0.19	1.3	0	1.6
A_51_P453736	NM_001143686	Apol11b	0.08	1.6	0	2.3	0.03	1.6
A_55_P2108248	NM_026639	Art4	0.9	1.1	0.68	1.2	0.02	1.6
A_51_P330213	NM_024184	Asf1b	0	1.7	0.04	1.4	0	1.6
A_55_P2170732	NM_178699	B930041F14Rik	0.53	1.1	0.82	1.1	0	1.6
A_55_P2012734	NM_178699	B930041F14Rik	0.38	1.1	0.26	1.2	0	1.6
A_55_P1983773	NM_001012273	Birc5	0	2.5	0	1.9	0.01	1.6
A_51_P125607	NM_176954	Brunol5	0.54	1.1	0.13	1.2	0	1.6
A_52_P488612	NM_027060	Btbd9	0.17	1.4	0.99	1	0.03	1.6
A_55_P2041075	NM_001081636	Ccnd3	0.36	1.1	0.17	1.2	0	1.6
A_51_P343252	NM_009842	Cd151	0.13	1.2	0.16	1.2	0	1.6
A_51_P181286	NM_001033122	Cd69	0.04	1.4	0	1.7	0	1.6
A_55_P2106150	NM_021790	Cenpk	0	1.6	0	1.6	0	1.6

A_55_P2077263	NM_021790	Cenpk	0	1.6	0.02	1.6	0	1.6
A_51_P367310	NM_028083	Chaf1b	0.17	1.4	0.07	1.6	0.01	1.6
A_55_P2124791	NM_001109991	Col18a1	0.2	1.2	0.28	1.2	0	1.6
A_55_P2059352	NM_001109991	Col18a1	0.21	1.2	0.14	1.3	0	1.6
A_51_P139108	NM_019696	Cpxm1	0.63	1.2	0.94	1.1	0.02	1.6
A_55_P2038106	NM_009978	Cst8	0.37	1.2	0.01	1.6	0	1.6
A_51_P251357	NM_016748	Ctps	0.28	1.2	0.39	1.2	0	1.6
A_55_P1980262	NM_001018063	Cxx1b	0.36	1.2	0.4	1.3	0	1.6
A_51_P252859	NM_010516	Cyr61	0.36	1.3	0.55	1.3	0.01	1.6
A_52_P592909	NM_026384	Dgat2	0.03	1.7	0.21	1.5	0.05	1.6
A_55_P2045258	NM_010119	Ehd1	0.01	1.5	0.06	1.4	0	1.6
A_51_P455807	NM_133838	Ehd4	0.07	1.3	0	1.5	0	1.6
A_51_P126437	NM_007930	Enc1	0.25	1.2	0.01	1.5	0	1.6
A_55_P2150343	NM_001037298	Fam38a	0.25	1.2	0.18	1.2	0	1.6
A_55_P2107502	NM_001081286	Fat1	0.95	-1	0.24	1.2	0	1.6
A_55_P2088615	NM_011812	Fbln5	0.36	1.2	0.11	1.3	0	1.6
A_55_P2011937	NM_001163359	Fignl1	0	1.7	0	1.8	0	1.6
A_51_P337089	NM_027014	Gins1	0.03	1.4	0	1.5	0	1.6
A_55_P2167123	XM_904714	Gm12839	0.49	1.3	0.3	1.4	0.02	1.6
A_55_P2012960	NM_024198	Gpx7	0.14	1.2	0.62	1.1	0	1.6
A_55_P2114776	NM_015764	Greb1	0.27	1.3	0.38	1.3	0.01	1.6
A_55_P2142251	NM_054045	Hist2h3c2	0.29	1.3	0.89	1.1	0.02	1.6
A_55_P2035424	NM_008278	Hpgd	0.27	1.3	0.05	1.5	0	1.6
A_55_P2085984	NM_010512	Igf1	0.73	1.1	0.9	1.1	0.01	1.6
A_51_P470079	NM_010555	Il1r2	0.01	1.5	0.07	1.4	0	1.6
A_52_P350554	NM_008420	Kcnb1	0.05	1.4	0.67	1.2	0	1.6
A_51_P218975	NM_173417	Kcns3	0.05	1.3	0.07	1.3	0	1.6
A_55_P2092286	XM_001481225	LOC100048780	0.02	1.5	0.01	1.7	0	1.6
A_51_P158210	NM_008564	Mcm2	0.01	1.5	0.01	1.7	0	1.6
A_51_P124535	NM_008590	Mest	0.55	1.2	0.55	1.2	0	1.6
A_55_P2005470	NM_029568	Mfap4	0.56	1.2	0.29	1.4	0.01	1.6
A_51_P386344	NM_172742	Mtmr10	0	1.4	0.01	1.3	0	1.6
A_55_P1967736	NM_027280	Nkd1	0.14	1.3	0.02	1.4	0	1.6
A_51_P195875	NM_010929	Notch4	0.01	1.4	0.01	1.4	0	1.6
A_55_P2148534	BC096461	Nr1d2	0.26	1.3	0	2	0	1.6
A_51_P506733	NM_001038845	P2rx7	0	1.4	0.11	1.2	0	1.6
A_55_P2035159	NM_029357	Pcdh1	0.02	1.3	0	1.5	0	1.6
A_55_P2008634	NM_053135	Pcdhb10	0.32	1.2	0.03	1.4	0	1.6
A_51_P173961	NM_178939	Pdrg1	0.47	1.2	0.67	1.2	0	1.6
A_55_P2087087	NM_001032378	Pecam1	0.02	1.5	0.6	1.2	0	1.6
A_51_P441426	NM_019932	Pf4	0.68	1.2	0.2	1.6	0.03	1.6
A_51_P204442	NM_028716	Phf19	0	1.7	0.01	1.7	0	1.6
A_55_P2083963	NM_001145644	Phgr1	0.97	-1	0.95	1.1	0.01	1.6
A_66_P119034	NM_013737	Pla2g7	0.8	1.1	0.04	1.4	0	1.6
A_55_P2159585	NM_178079	Pm20d1	0.48	1.2	0.46	1.3	0	1.6
A_55_P1954086	NM_015784	Postn	0.04	1.3	0.08	1.3	0	1.6
A_51_P433026	NM_028922	Ppapdc2	0	1.4	0	1.3	0	1.6
A_51_P224564	NM_176833	Ppm1f	0.02	1.3	0.03	1.3	0	1.6
A_51_P332652	NM_172574	Pqlc3	0.6	1.1	0.44	1.2	0	1.6
A_55_P1994309	NM_008967	Ptgir	0.45	1.2	0.31	1.3	0	1.6
A_55_P2006327	NM_008981	Ptprg	0.15	1.2	0.02	1.5	0	1.6
A_55_P1992084	NM_008981	Ptprg	0.76	1.1	0.01	1.4	0	1.6
A_55_P2036007	NM_198409	Rai2	0.07	1.4	0.69	1.2	0	1.6
A_52_P89567	NM_007483	Rhob	0.14	1.4	0.48	1.3	0	1.6
A_52_P577729	NM_175388	Rnf169	0.15	1.2	0.01	1.4	0	1.6

A_55_P1985070	NM_019413	Robo1	0.04	1.7	0.63	1.3	0.04	1.6
A_55_P2083919	NM_175549	Robo2	0.78	1.1	0.03	1.5	0	1.6
A_52_P357133	NM_053267	Selm	0.09	1.3	0.36	1.2	0	1.6
A_52_P73620	NM_198028	Serpinb10	0.13	1.4	0	1.7	0	1.6
A_51_P268094	NM_009255	Serpine2	0	1.4	0.33	1.2	0	1.6
A_55_P2043554	NM_009195	Slc12a4	0.39	1.2	0.1	1.3	0	1.6
A_51_P514319	NM_172892	Slc13a4	0.55	1.2	0.6	1.3	0.02	1.6
A_55_P2187076	NM_011430	Sncg	0.02	1.3	0.03	1.3	0	1.6
A_55_P2131899	NM_009238	Sox4	0.24	1.2	0.86	1.1	0	1.6
A_55_P2026223	NM_011373	St6galnac4	0.08	1.3	0.72	1.1	0	1.6
A_55_P2068673	NM_025285	Stmn2	0.07	1.2	0	1.5	0	1.6
A_52_P193925	NM_028072	Sulf2	0.62	1.1	0.98	1	0	1.6
A_52_P62444	NM_013681	Syn2	0.51	1.3	0.67	1.3	0.03	1.6
A_55_P2048279	NM_205820	Tlr13	0.23	1.2	0.06	1.4	0	1.6
A_52_P368306	NM_026433	Tmem100	0.14	1.5	0.31	1.4	0.04	1.6
A_51_P418116	NM_146162	Tmem119	0.4	1.1	0	1.5	0	1.6
A_52_P112110	NM_145987	Tmem82	0.51	1.2	0.22	1.4	0.01	1.6
A_55_P1962937	NM_031254	Trem2	0.95	-1	0.98	1	0.03	1.6
A_51_P220343	NM_018865	Wisp1	0.92	1.1	0.15	1.5	0.01	1.6
A_52_P148678	AK169695		0.22	1.2	0.01	1.6	0.01	1.5
A_52_P425092			0.72	1.1	0.92	1.1	0.04	1.5
A_55_P1973858			0.48	1.2	0.96	-1	0.03	1.5
A_55_P2114342	AK155479		0.77	1.1	0.87	1.1	0.02	1.5
A_51_P191893	AK163489		0.23	1.3	0.17	1.4	0.02	1.5
A_30_P01021251			0.02	1.3	0.03	1.3	0	1.5
A_30_P01032753			0.09	1.2	0.04	1.3	0	1.5
A_55_P2108693			0.16	1.2	0.74	1.1	0	1.5
A_30_P01023701			0.32	1.2	0.17	1.3	0	1.5
A_30_P01031939			0.25	1.2	0.53	1.2	0	1.5
A_55_P2077628	AK180254		0.69	1.1	0.74	1.1	0	1.5
A_30_P01024470			0.1	1.3	0.97	-1	0	1.5
A_30_P01020003			0.75	1.1	0.75	1.1	0	1.5
A_51_P378381	AK017076		0.17	1.2	0.06	1.4	0	1.5
A_30_P01019010			0.64	1.1	0.47	1.2	0	1.5
A_30_P01028302			0	1.2	0.01	1.3	0	1.5
A_30_P01028961			0.12	1.2	0.11	1.3	0	1.5
A_52_P431859	AK167772		0.01	1.3	0.1	1.2	0	1.5
A_30_P01027827			0.53	1.1	0.13	1.3	0	1.5
A_51_P214470	NM_173744	2610019F03Rik	0	1.4	0.25	1.2	0	1.5
A_55_P2026982	NM_173744	2610019F03Rik	0.11	1.2	0.02	1.3	0	1.5
A_55_P1960049	NM_027419	2810408A11Rik	0.68	1.1	0.7	1.1	0	1.5
A_51_P488554	NM_026543	3010026O09Rik	0	1.6	0.04	1.3	0	1.5
A_51_P301636	NM_001109685	9030409G11Rik	0.11	1.3	0.11	1.4	0	1.5
A_66_P122086	NM_001039720	9030619P08Rik	0.12	1.4	0.51	1.2	0.01	1.5
A_55_P2470474	AK087205	9530082P21Rik	0.02	1.3	0.01	1.5	0	1.5
A_51_P249118	NM_008830	Abcb4	0.77	1.1	0.87	1.1	0.01	1.5
A_52_P489778	NM_178688	Ablim1	0.04	1.3	0	1.5	0	1.5
A_55_P2055819	NM_001037722	Adam15	0.48	1.2	0.31	1.3	0	1.5
A_55_P1988623	NM_001164099	Add3	0	1.4	0	1.6	0	1.5
A_55_P2320313	BC058714	AI852064	0.17	1.3	0.28	1.3	0.01	1.5
A_55_P2007155	NM_134072	Akr1c14	0.21	1.2	0.34	1.2	0	1.5
A_65_P18433	NM_001024604	Ankrd28	0.72	1.1	0.02	1.3	0	1.5
A_55_P2143070	NM_007494	Ass1	0.2	1.2	0.06	1.4	0	1.5
A_55_P2119985	NM_130862	Baiap2	0.22	1.3	0.21	1.3	0.01	1.5
A_55_P2060991	NM_181681	BC005764	0.83	-1.1	0.91	1.1	0.01	1.5

A_52_P627269	NM_198171	BC015286	0	1.9	0.04	1.5	0.01	1.5
A_55_P2029846	NM_001113283	BC031353	0.08	1.3	0.02	1.6	0	1.5
A_51_P272553	NM_011498	Bhlhe40	0.13	1.3	0.04	1.5	0.01	1.5
A_55_P2355330	NM_145613	C1qtnf5	0.73	1.1	0.85	1.1	0.02	1.5
A_55_P2158102	NM_009921	Camp	0.02	2.1	0.04	2	0.13	1.5
A_55_P2080163	NM_025821	Carhsp1	0	1.3	0.01	1.2	0	1.5
A_51_P414548	NM_007611	Casp7	0.33	1.2	0.42	1.2	0	1.5
A_52_P39083	NM_178793	Ccbe1	0.72	1.1	0.02	1.4	0	1.5
A_51_P436652	NM_013654	Ccl7	0.98	1	0.95	-1.1	0.02	1.5
A_51_P481920	NM_009828	Ccna2	0	2.4	0.02	2	0.06	1.5
A_51_P238448	NM_007632	Ccnd3	0.2	1.2	0.25	1.3	0	1.5
A_55_P1960216	NM_009842	Cd151	0.2	1.3	0.94	1.1	0	1.5
A_52_P422494	NM_145634	Cd300lf	0.77	1.1	0.19	1.2	0	1.5
A_51_P125135	NM_026410	Cdca5	0	1.9	0.03	1.6	0.01	1.5
A_55_P1988183	NM_007670	Cdkn2b	0.12	1.2	0.75	1.1	0	1.5
A_55_P2085181	NM_028083	Chaf1b	0.1	1.3	0.04	1.5	0.01	1.5
A_52_P162099	NM_001004140	Ckap2	0	2.4	0.05	1.7	0.02	1.5
A_52_P624434	NM_175526	Clec1a	0.12	1.4	0.16	1.5	0.04	1.5
A_55_P2148906	NM_028870	Cltb	0.01	1.2	0.21	1.2	0	1.5
A_55_P2001250	NM_153107	Cpz	0.61	1.2	0.53	1.3	0.01	1.5
A_55_P2052240	NM_018827	Crlf1	0.58	1.1	0.42	1.2	0	1.5
A_51_P489522	NM_007797	Ctla2b	0	1.4	0.01	1.4	0	1.5
A_55_P1985591	NM_024170	Cxx1a	0.21	1.3	0.92	1.1	0.02	1.5
A_55_P2133266	NM_028375	Cxx1c	0.49	1.2	0.45	1.2	0	1.5
A_55_P2344608	AK081893	D7Wsu130e	0.72	1.1	0.08	1.5	0.01	1.5
A_51_P493037	NM_018831	Dclre1a	0.02	1.5	0.38	1.3	0.02	1.5
A_52_P104824	NM_019670	Diap3	0.51	1.2	0.46	1.2	0	1.5
A_55_P2280821	NM_015802	Dlc1	0.03	1.4	0.06	1.4	0	1.5
A_52_P583458	NM_010093	E2f3	0.06	1.2	0	1.4	0	1.5
A_51_P161323	NM_175540	Eda2r	0.01	1.3	0.01	1.4	0	1.5
A_55_P2097022	NM_025613	Eid1	0.12	1.2	0.18	1.2	0	1.5
A_51_P260639	NM_028811	Elp3	0.26	1.2	0.92	1.1	0.01	1.5
A_55_P2023762	NM_007936	Epha4	0.61	1.1	0.42	1.2	0	1.5
A_51_P382925	NM_010175	Fadd	0.28	1.2	0.54	1.2	0	1.5
A_55_P2007389	NM_001014995	Fam189b	0.13	1.2	0.03	1.3	0	1.5
A_51_P264527	NM_019833	Fam69b	0.01	1.6	0.26	1.3	0.02	1.5
A_55_P2025790	NM_145946	Fanci	0.16	1.3	0.25	1.3	0.01	1.5
A_55_P2038757	NM_133754	Fblim1	0.27	1.2	0.23	1.3	0.01	1.5
A_55_P2100884	NM_010218	Fjx1	0.79	1.1	0.6	1.2	0	1.5
A_52_P222350	NM_134080	Flnb	0.01	1.4	0.13	1.3	0	1.5
A_55_P2079089	NM_024243	Fuca1	0.4	1.2	0.93	1.1	0	1.5
A_51_P273684	NM_028803	Gbe1	0.12	1.3	0.03	1.5	0	1.5
A_55_P1980119	NM_024472	Gltpd1	0.42	1.2	0.24	1.3	0	1.5
A_55_P2175451	XM_001474886	Gm9791	0	1.3	0.02	1.2	0	1.5
A_55_P2016049	NM_001163014	Gp6	0.22	1.4	0.11	1.6	0.03	1.5
A_52_P68221	NM_016886	Gria3	0.17	1.3	0.1	1.4	0	1.5
A_51_P260169	NM_010360	Gstm5	0.05	1.3	0.63	1.2	0	1.5
A_55_P1996973	NM_029000	Gvin1	0.37	1.3	0.24	1.4	0.04	1.5
A_55_P2084652	NM_178183	Hist1h2ak	0.01	1.3	0.09	1.3	0	1.5
A_55_P2109128	NM_178194	Hist1h2be	0.1	1.3	0.04	1.5	0.01	1.5
A_55_P2131438	NM_178212	Hist2h2aa2	0.16	1.3	0.31	1.2	0	1.5
A_55_P2036813	NM_030082	Hist3h2ba	0.41	1.2	0.69	1.2	0.01	1.5
A_52_P586004	NM_001033245	Hk3	0.33	1.2	0.72	1.2	0.01	1.5
A_51_P388819	NM_174998	Hpcal4	0.02	1.6	0.11	1.5	0.01	1.5
A_55_P2085974	NM_010512	Igf1	0.66	1.2	0.61	1.2	0.02	1.5

A_51_P155843	NM_001162884	Igsf10	0.59	1.1	0.31	1.3	0.01	1.5
A_66_P113749	NM_013563	Il2rg	0.17	1.3	0.49	1.2	0.01	1.5
A_52_P588881	NM_001033484	Iqgap3	0	2.2	0.02	1.6	0.01	1.5
A_55_P2123502	NM_023844	Jam2	0.13	1.2	0.03	1.3	0	1.5
A_55_P2064328	NM_008481	Lama2	0.16	1.2	0.34	1.2	0	1.5
A_55_P2064333	NM_010680	Lama3	0.74	1.1	0.98	-1	0.03	1.5
A_55_P2017939	NM_011836	Lamc3	1	-1	0.84	1.1	0	1.5
A_51_P465281	NM_008495	Lgals1	0.13	1.3	0.57	1.2	0	1.5
A_55_P1957459	NM_013532	Lilrb4	0.97	1	0.99	-1	0	1.5
A_52_P137765	NM_019390	Lmna	0.25	1.3	0.97	-1	0.01	1.5
A_55_P2094641	XM_001474594	LOC100044122	0.2	1.2	0.18	1.3	0	1.5
A_55_P2085485	NM_022654	Lrdd	0.05	1.2	0.15	1.2	0	1.5
A_51_P513941	NM_013587	Lrpap1	0.34	1.2	0.34	1.2	0	1.5
A_55_P2180086	NM_175124	Lrrc28	0.55	1.1	0.09	1.3	0	1.5
A_51_P233160	NM_027309	Lysmd2	0.02	1.4	0	1.5	0	1.5
A_51_P451458	NM_174857	Mamdc2	0.31	1.2	0.39	1.3	0.01	1.5
A_55_P2055257	NM_173740	Maoa	0.72	1.1	0.09	1.3	0	1.5
A_55_P1971897	NM_010784	Mdk	0.91	1.1	0.89	1.1	0.03	1.5
A_52_P674309	NM_013826	Mocs2	0.27	1.2	0.46	1.2	0.01	1.5
A_51_P193146	NM_028595	Ms4a6c	0.07	1.3	0.09	1.4	0.01	1.5
A_52_P607128	NM_031195	Msr1	0.04	1.3	0.02	1.3	0	1.5
A_55_P1965333	NM_029090	Nat15	0	1.3	0	1.4	0	1.5
A_55_P2157770	NM_001160165	Neu2	0.39	1.2	0.21	1.3	0	1.5
A_55_P2111907	NM_027988	Noxo1	0.75	1.1	1	-1	0	1.5
A_51_P424338	NM_008706	Nqo1	0.7	1.1	0.45	1.3	0.03	1.5
A_55_P1985433	NM_178591	Nrg1	0.73	1.1	0.36	1.2	0	1.5
A_51_P123077	NM_011955	Nubp1	0.15	1.2	0.69	1.1	0	1.5
A_51_P240453	NM_133851	Nusap1	0	1.9	0.06	1.5	0.02	1.5
A_51_P189361	NM_027950	Osgin1	0.2	1.2	0	1.4	0	1.5
A_51_P240421	NM_053139	Pcdhb14	0.94	1	0.23	1.3	0	1.5
A_55_P2020577	NM_008788	Pcolce	0.68	1.1	0.82	1.1	0	1.5
A_51_P258493	NM_011067	Per3	0.35	1.3	0	1.7	0.01	1.5
A_51_P363801	NM_023217	Pgpep1	0.16	1.3	0.65	1.1	0	1.5
A_55_P2054409	NM_011089	Pira2	0.98	-1	0.65	1.2	0	1.5
A_55_P2433218	NM_019788	Pldn	0.55	1.1	0.89	1.1	0	1.5
A_55_P1983588	NM_022995	Pmepa1	0.53	1.2	0.24	1.3	0.01	1.5
A_55_P1965150	NM_021549	Pnkp	0.02	1.3	0.04	1.2	0	1.5
A_55_P2007273	NM_011132	Pole	0	1.5	0	1.5	0	1.5
A_55_P1995055	NM_001030296	Prr7	0.38	1.2	0.15	1.3	0	1.5
A_55_P2149951	NM_019412	Prx	0.1	1.3	0.16	1.3	0	1.5
A_55_P2007871	NM_207232	Ptpdc1	0.64	1.1	0	1.4	0	1.5
A_55_P2116465	NM_008981	Ptprg	0.68	1.1	0.04	1.5	0	1.5
A_55_P1956567	NM_023852	Rab3c	0.45	1.2	0.32	1.4	0.04	1.5
A_55_P2107155	NM_029182	Rasd2	0.16	1.4	0.02	1.8	0.08	1.5
A_51_P136888	NM_009029	Rb1	0.01	1.3	0	1.4	0	1.5
A_52_P117325	NM_011250	Rbl2	0.12	1.3	0.51	1.2	0	1.5
A_55_P2002122	NM_173402	Rgs12	0	1.4	0	1.5	0	1.5
A_55_P2083929	NM_175549	Robo2	0.4	1.2	0.72	1.1	0	1.5
A_55_P2304864	NM_011282	Ros1	0.11	1.4	0.69	1.2	0.01	1.5
A_51_P482633	NM_019924	Rps6ka4	0.02	1.3	0.14	1.2	0	1.5
A_51_P326191	NM_009251	Serpina3g	0	2.7	0.27	1.7	0.22	1.5
A_55_P2006008	NM_025429	Serpinb1a	0.27	1.3	0.4	1.3	0.02	1.5
A_51_P204402	NM_011369	Shcbp1	0	2	0.08	1.5	0.01	1.5
A_51_P156434	NM_027460	Slc25a33	0.09	1.3	0.05	1.4	0	1.5
A_51_P300572	NM_009579	Slc30a1	0.05	1.4	0.02	1.5	0	1.5

A_51_P366672	NM_153170	Slc36a2	0.52	1.2	0.2	1.4	0.02	1.5
A_55_P1989673	NM_033314	Slco2a1	0.73	1.1	0.46	1.3	0.04	1.5
A_51_P183812	NM_011410	Slfn4	0.16	1.2	0.01	1.4	0	1.5
A_51_P301804	NM_009177	St3gal1	0.16	1.2	0.49	1.2	0	1.5
A_51_P425737	NM_025935	Tbc1d7	0.29	1.3	0.6	1.2	0.01	1.5
A_55_P2152035	NM_001163763	Tcf19	0	1.6	0.06	1.5	0.01	1.5
A_51_P336721	NM_025372	Tipin	0	1.5	0.02	1.5	0	1.5
A_52_P213889	NM_172476	Tmc7	0.37	1.2	0.58	1.2	0.01	1.5
A_66_P112482	NM_145599	Tmem184c	0.18	1.2	0.14	1.3	0	1.5
A_52_P325477	NM_053169	Trim16	0.07	1.3	0.8	1.1	0	1.5
A_55_P2067453	NM_028841	Tspan17	0.35	1.1	0.31	1.2	0	1.5
A_55_P2069597	NM_028841	Tspan17	0.19	1.2	0.63	1.1	0	1.5
A_51_P433870	NM_009506	Vegfc	0.35	1.2	0.31	1.3	0.01	1.5
A_51_P316951	NM_001167860	Wipf3	0.26	1.3	0.26	1.3	0	1.5
A_51_P390804	NM_016873	Wisp2	0.84	1.1	0.95	1.1	0.01	1.5
A_51_P312997	NM_012017	Zfp346	0.12	1.2	0.01	1.3	0	1.5
A_51_P126626	NM_145459	Zfp503	0.04	1.4	0.02	1.5	0	1.5
A_30_P01019123			0	1.8	0.01	1.7	0.04	1.4
A_55_P2063146			0	1.6	0.17	1.3	0.03	1.4
A_55_P2056493			0	1.6	0.41	1.3	0.07	1.4
A_51_P241213	XM_896385	A030005L19Rik	0.23	1.2	0	1.5	0	1.4
A_55_P2030721	NM_172756	Ankle1	0.01	1.6	0.56	1.2	0.04	1.4
A_52_P628067	NM_013538	Cdca3	0	1.7	0.25	1.3	0.03	1.4
A_51_P133612	NM_026014	Cdt1	0	1.4	0	1.7	0	1.4
A_51_P227004	NM_016904	Cks1b	0.02	1.5	0.16	1.4	0.04	1.4
A_55_P2062598	NM_178609	E2f7	0	1.7	0.02	1.7	0.02	1.4
A_55_P2269819	NM_183187	Fam107a	0	2.1	0.26	1.4	0.08	1.4
A_55_P2114779	NM_015764	Greb1	0.25	1.3	0.01	1.6	0.03	1.4
A_52_P330984	NM_001103182	Lin9	0	1.3	0.01	1.6	0	1.4
A_55_P2076866	NM_001162977	Megf6	0.03	1.4	0.01	1.5	0	1.4
A_55_P2013336	NM_010790	Melk	0	1.6	0.12	1.4	0.04	1.4
A_55_P2073377	NM_001081117	Mki67	0	2.4	0	1.9	0.06	1.4
A_51_P405606	NM_008681	Ndrg1	0.02	1.4	0.02	1.5	0.01	1.4
A_55_P1972040	NM_015760	Nox4	0.42	1.2	0	1.6	0.01	1.4
A_55_P2116165	NM_133232	Pfkfb3	0	1.3	0	1.5	0	1.4
A_51_P477121	NM_021451	Pmaip1	0.11	1.3	0.01	1.5	0	1.4
A_55_P2089233	NM_011136	Pou2af1	0.3	1.4	0.01	2.2	0.24	1.4
A_51_P168632	NM_008921	Prim1	0	1.4	0	1.5	0	1.4
A_51_P170959	AK005011	Proz	0.1	1.5	0.04	1.7	0.13	1.4
A_51_P477682	NM_008939	Prss12	0.28	1.2	0.02	1.5	0.02	1.4
A_52_P283524	NM_022980	Rcan3	0.14	1.3	0.02	1.5	0.01	1.4
A_55_P2002129	NM_173402	Rgs12	0.03	1.5	0.37	1.3	0.03	1.4
A_51_P474454	NM_026001	Rnaseh2b	0.01	1.4	0	1.5	0	1.4
A_55_P2043322	NM_153762	Rnf26	0	1.5	0.63	1.1	0	1.4
A_55_P2104975	NM_001168294	Serpina3f	0	2.6	0.03	1.9	0.22	1.4
A_55_P1953301	NM_146126	Sord	0.95	1	0.03	1.5	0.02	1.4
A_51_P513530	NM_017407	Spag5	0	1.6	0.1	1.4	0.02	1.4
A_51_P369200	NM_028109	Tpx2	0.01	1.7	0.37	1.3	0.08	1.4
A_51_P451151	NM_026785	Ube2c	0	1.8	0.13	1.5	0.04	1.4
A_55_P1985788	NM_198092	Usp2	0.6	1.2	0.02	1.7	0.06	1.4
A_30_P01023554			0	1.6	0.02	1.5	0.1	1.3
A_30_P01017632			0	1.5	0	1.5	0.04	1.3
A_66_P120380	NM_177820	Apol10b	0.22	1.3	0.04	1.5	0.07	1.3
A_55_P2186648	NM_007525	Bard1	0.01	1.3	0	1.5	0.01	1.3
A_55_P1996946	NM_023223	Cdc20	0	2	0.02	1.6	0.07	1.3

A_55_P2158011	NM_026412	D2Ertd750e	0	1.7	0.22	1.3	0.13	1.3
A_55_P1976127	NM_007900	Ect2	0.02	1.6	0.2	1.4	0.17	1.3
A_52_P370935	NM_133236	Glcci1	0.04	1.2	0	1.5	0	1.3
A_51_P109144	NM_025768	Grtp1	0	1.5	0.2	1.2	0	1.3
A_52_P498208	NM_178183	Hist1h2ak	0	1.5	0.3	1.2	0.01	1.3
A_55_P2084631	NM_178184	Hist1h2an	0	1.5	0.14	1.3	0.01	1.3
A_55_P2087622	NM_010215	Il4i1	0.07	1.3	0	1.6	0.05	1.3
A_55_P2079272	NM_001130868	Kars	0.01	1.5	0.95	1	0.04	1.3
A_51_P481398	NM_010615	Kif11	0.03	1.5	0.25	1.4	0.21	1.3
A_55_P2056654	NM_145588	Kif22	0	1.7	0.03	1.4	0.07	1.3
A_55_P2062543	NM_053173	Kifc1	0	1.6	0.09	1.4	0.11	1.3
A_51_P202074	NM_146171	Ncapd2	0	1.5	0.11	1.3	0.05	1.3
A_52_P580582	NM_008725	Nppa	0	3.9	0.98	1.1	0.75	1.3
A_51_P230098	NM_023209	Pbk	0.03	1.8	0.4	1.4	0.42	1.3
A_55_P1988083	NM_145150	Prc1	0.01	1.8	0.28	1.4	0.32	1.3
A_51_P487999	NM_028232	Sgol1	0	2.1	0.04	1.5	0.1	1.3
A_55_P2172274	NM_008017	Smc2	0.02	1.4	0.01	1.5	0.02	1.3
A_55_P2004801	NM_001040435	Tacc3	0.02	1.6	0.13	1.5	0.21	1.3
A_52_P151320	NM_025566	Tnfaip8l1	0	1.5	0.57	1.2	0.06	1.3
A_30_P01030240			0.04	1.5	0.23	1.4	0.43	1.2
A_30_P01020075			0.01	1.5	0.3	1.2	0.11	1.2
A_30_P01028287			0	1.5	0.29	1.2	0.14	1.2
A_30_P01018888			0.39	1.2	0.04	1.5	0.18	1.2
A_30_P01021644			0.46	1.2	0	1.5	0.3	1.2
A_52_P354373	XM_001481164	1190002F15Rik	0	1.7	0.05	1.5	0.19	1.2
A_51_P472217	NM_001081085	2010317E24Rik	0.03	1.6	0.29	1.4	0.31	1.2
A_55_P1988228	NM_009791	Aspm	0	1.9	0.06	1.6	0.4	1.2
A_55_P1980636	NM_011497	Aurka	0	1.6	0.05	1.4	0.23	1.2
A_55_P2026139	NM_001013773	BC055004	0.01	1.5	0.55	1.2	0.14	1.2
A_55_P1964648	NM_001037719	Btla	0.61	1.3	0.04	2.1	0.6	1.2
A_55_P2065671	NM_172301	Ccnb1	0.01	1.5	0.07	1.5	0.49	1.2
A_51_P457528	NM_007630	Ccnb2	0	1.7	0.24	1.4	0.27	1.2
A_51_P155142	NM_026560	Cdca8	0	1.6	0.16	1.3	0.22	1.2
A_52_P30989	NM_028222	Cdkn3	0.01	1.6	0.27	1.3	0.25	1.2
A_51_P164014	NM_173762	Cenpe	0	1.8	0.01	1.6	0.32	1.2
A_51_P133137	NM_009004	Kif20a	0	1.9	0.11	1.4	0.16	1.2
A_55_P2109717	NM_183046	Kif20b	0.01	1.6	0.06	1.5	0.25	1.2
A_51_P130079	NM_008511	Lrmp	0.21	1.3	0.02	1.5	0.49	1.2
A_52_P190647	NM_016662	Mxd3	0	1.5	0.83	1.1	0.47	1.2
A_55_P2127702	NM_012025	Racgap1	0	1.6	0.13	1.3	0.18	1.2
A_52_P550173	NM_013730	Slamf1	0.07	1.3	0.04	1.5	0.22	1.2
A_55_P2091191	NM_172980	Slc28a2	0.62	1.1	0.03	1.5	0.44	1.2
A_55_P1995205	NM_011623	Top2a	0.02	1.6	0.38	1.3	0.35	1.2
A_55_P1983768	NM_009689	Birc5	0	1.5	0.58	1.2	0.45	1.1
A_52_P70796	NM_007551	Cxcr5	0.48	1.2	0.01	1.9	0.7	1.1
A_51_P455897	NM_144526	Fam64a	0	2.3	0.03	1.5	0.8	1.1
A_55_P2103706	XM_485921	Gm5593	0.04	1.5	0.34	1.3	0.68	1.1
A_55_P2192662	NM_001122899	Lepr	0.01	1.5	0.09	1.3	0.47	1.1
A_52_P429450	NM_008694	Ngp	0.01	1.7	0.13	1.5	0.61	1.1
A_66_P134542	NM_028390	Anln	0	1.6	0.26	1.3	0.93	1
A_66_P121459	NM_007681	Cenpa	0	1.6	0.35	1.2	0.9	1
A_55_P1995537	NM_010824	Mpo	0.04	1.5	0.4	1.3	0.98	1
A_55_P2033600	NM_019992	Stap1	0.88	1.1	0.04	1.6	0.98	1
A_51_P335969	NM_010043	Des	0.03	1.5	0.87	-1.1	0.98	-1
A_66_P130916	NM_010389	H2-Ob	0.41	1.2	0.02	1.7	0.98	-1

A_55_P2030938	NM_025863	Trim59	0.1	1.4	0.02	1.6	0.98	-1
A_30_P01018833			0	-1.5	0.72	-1.1	0.74	-1.1
A_30_P01033210			0	-1.5	0.56	-1.2	0.09	-1.2
A_55_P2147220	NM_016895	Ak2	0.03	-1.5	0.92	-1.1	0.22	-1.2
A_55_P2099810	NM_031185	Akap12	0.01	-1.5	0.92	-1.1	0.13	-1.2
A_51_P393518	NM_001024720	Hmcn1	0.07	-1.4	0.01	-1.7	0.32	-1.2
A_55_P2151822	NM_026376	Plxnd1	0	-1.5	0.01	-1.4	0.06	-1.2
A_55_P2040168	NM_008885	Pmp22	0.03	-1.5	0.57	-1.2	0.27	-1.2
A_51_P452629	NM_011905	Tlr2	0.65	-1.2	0.04	-1.6	0.56	-1.2
A_30_P01020112			0.02	-1.5	0.6	-1.2	0.12	-1.3
A_51_P114616	NM_016767	Batf	0.24	-1.2	0.02	-1.5	0.04	-1.3
A_66_P103027	NM_172732	Clec9a	0.03	-1.6	0.3	-1.4	0.18	-1.3
A_51_P501844	NM_175475	Cyp26b1	0	-3.6	0.02	-3.8	0.73	-1.3
A_55_P2175752	NM_001081975	Mfap1b	0.04	-1.5	0.89	-1.1	0.19	-1.3
A_55_P2119011	NM_133885	Osbpl9	0.01	-1.5	0.72	-1.1	0.08	-1.3
A_55_P1973886	NM_080843	Socs4	0	-1.5	0.35	-1.2	0	-1.3
A_52_P461343	NM_023478	Upk3a	0.19	-1.5	0.04	-2	0.32	-1.3
A_30_P01026086			0.04	-1.5	0.6	-1.2	0.03	-1.4
A_30_P01028248			0.01	-1.5	0.3	-1.3	0.02	-1.4
A_30_P01018146			0	-1.5	0.29	-1.2	0	-1.4
A_30_P01032901			0	-1.5	0.42	-1.2	0.01	-1.4
A_55_P2104387			0	-1.5	0.1	-1.2	0	-1.4
A_55_P2201454	AK142062	2310079F09Rik	0	-1.5	0.8	-1.1	0	-1.4
A_55_P2437763	NR_002849	8430431K14Rik	0	-1.6	0.09	-1.3	0	-1.4
A_55_P1971574	NM_030721	Acox3	0	-1.5	0.24	-1.3	0	-1.4
A_55_P2162782	NM_009643	Ahnak	0	-1.5	0.78	-1.1	0.01	-1.4
A_51_P241995	NM_016919	Col5a3	0.16	-1.3	0.05	-1.5	0.02	-1.4
A_55_P2259500	AK083809	D130012P04Rik	0	-1.5	0.17	-1.3	0	-1.4
A_55_P1979242	NM_134110	Kcne2	0.46	-1.2	0.02	-1.6	0.05	-1.4
A_51_P413147	NM_008693	Klk1b3	0.04	-1.5	0.09	-1.5	0.03	-1.4
A_66_P118165	XM_001471574	LOC100038935	0	-1.5	0.15	-1.3	0	-1.4
A_55_P2183518	XM_001473350	LOC100044930	0	-1.5	0.46	-1.2	0.03	-1.4
A_55_P2114863	NM_001166251	Mgll	0	-1.6	0.08	-1.3	0	-1.4
A_55_P2002712	NM_010804	Mllt10	0.01	-1.5	0.93	-1.1	0.01	-1.4
A_52_P409833	NM_008872	Plat	0.15	-1.3	0.01	-1.7	0.02	-1.4
A_55_P1956978	NM_011602	Tln1	0.02	-1.5	0.42	-1.3	0.04	-1.4
A_52_P799815	NM_001025606	Tmem171	0.18	-1.3	0.01	-1.7	0.08	-1.4
A_55_P2127174	NM_170759	Zfp628	0	-1.5	0.34	-1.2	0	-1.4
A_55_P1955733	XM_001477303		0	-1.5	0.02	-1.3	0	-1.5
A_30_P01031683			0	-1.5	0.01	-1.3	0	-1.5
A_55_P2021923	AB241120		0	-1.5	0.15	-1.2	0	-1.5
A_30_P01027463			0	-1.5	0.03	-1.3	0	-1.5
A_30_P01025590			0	-1.5	0.13	-1.3	0	-1.5
A_55_P2050988			0	-1.5	0.48	-1.2	0	-1.5
A_30_P01026526			0	-1.5	0.86	-1.1	0	-1.5
A_30_P01028970			0	-1.5	0.07	-1.3	0	-1.5
A_30_P01027120			0.92	1.1	0.95	-1.1	0.04	-1.5
A_30_P01022168			0.32	-1.3	0.74	-1.2	0.04	-1.5
A_30_P01020907			0.46	-1.2	1	1	0.03	-1.5
A_30_P01033617			0.19	-1.4	0.68	-1.2	0.03	-1.5
A_30_P01019565			0.13	-1.4	0.64	-1.2	0.02	-1.5
A_55_P2004119			0.27	-1.3	0.8	-1.1	0.02	-1.5
A_30_P01022987			0.05	-1.4	0.21	-1.3	0.01	-1.5
A_55_P2074080			0.17	-1.3	0.72	-1.2	0.01	-1.5
A_55_P2069787			0.19	-1.3	0.63	-1.2	0.01	-1.5

A_30_P01019537			0.14	-1.3	0.32	-1.3	0.01	-1.5
A_55_P2009857			0.08	-1.4	0.78	-1.1	0.01	-1.5
A_55_P1962707			0.09	-1.4	0.96	1	0.01	-1.5
A_30_P01031878			0.09	-1.3	0.35	-1.3	0.01	-1.5
A_55_P2118857			0.07	-1.4	0.36	-1.3	0.01	-1.5
A_30_P01022683			0.01	-1.4	0.87	-1.1	0	-1.5
A_55_P2170834	CA461216		0	-1.4	0.1	-1.3	0	-1.5
A_30_P01028236			0.03	-1.3	0.88	-1.1	0	-1.5
A_30_P01028215			0.03	-1.3	0.16	-1.3	0	-1.5
A_55_P2077671			0.08	-1.3	0.92	-1.1	0	-1.5
A_66_P110798	AK145507		0.04	-1.3	0.77	-1.1	0	-1.5
A_55_P2145059	AF487898		0.15	-1.3	0.56	-1.2	0	-1.5
A_30_P01026329			0	-1.4	0.1	-1.3	0	-1.5
A_66_P101764	AK042950		0	-1.4	0.45	-1.2	0	-1.5
A_55_P1973995	AK076985		0.09	-1.2	0.01	-1.3	0	-1.5
A_30_P01030141			0	-1.3	0.02	-1.3	0	-1.5
A_30_P01023672			0	-1.4	0.17	-1.3	0	-1.5
A_55_P1977003	XM_001475321		0.37	-1.2	0.1	-1.4	0	-1.5
A_30_P01032631			0.03	-1.3	0.38	-1.2	0	-1.5
A_30_P01029295			0	-1.4	0.11	-1.2	0	-1.5
A_30_P01031898			0.06	-1.3	0.87	-1.1	0	-1.5
A_30_P01032612			0.01	-1.4	0.28	-1.2	0	-1.5
A_30_P01030784			0.04	-1.4	0.27	-1.3	0	-1.5
A_30_P01022868			0	-1.4	0.05	-1.2	0	-1.5
A_55_P1991239	AK051413		0	-1.3	0.02	-1.3	0	-1.5
A_55_P2184551	AK050822		0.01	-1.4	0.12	-1.3	0	-1.5
A_55_P2131481			0.03	-1.4	0.59	-1.2	0	-1.5
A_30_P01026288			0.03	-1.3	0.5	-1.1	0	-1.5
A_30_P01027369			0.26	-1.2	0.14	-1.3	0	-1.5
A_30_P01018341			0.07	-1.2	0.16	-1.2	0	-1.5
A_30_P01029815			0.02	-1.4	0.4	-1.2	0	-1.5
A_52_P163515			0.31	-1.2	0.04	-1.4	0	-1.5
A_30_P01031102			0.03	-1.4	0.81	-1.1	0	-1.5
A_30_P01020298			0	-1.4	0.07	-1.3	0	-1.5
A_30_P01032882			0.02	-1.4	0.23	-1.2	0	-1.5
A_30_P01023896			0.02	-1.3	0.15	-1.2	0	-1.5
A_55_P2011692	AK164389		0.26	-1.2	0.87	-1.1	0	-1.5
A_30_P01023888			0.15	-1.3	0.61	-1.2	0	-1.5
A_30_P01028077			0	-1.4	0.06	-1.2	0	-1.5
A_30_P01031736			0.09	-1.2	0.56	-1.1	0	-1.5
A_30_P01019049			0	-1.4	0.15	-1.2	0	-1.5
A_30_P01028021			0.05	-1.4	0.84	-1.1	0	-1.5
A_30_P01028353			0.86	-1.1	0.89	-1.1	0	-1.5
A_30_P01025092			0.03	-1.3	0.43	-1.2	0	-1.5
A_30_P01021970			0.12	-1.3	0.31	-1.3	0	-1.5
A_30_P01020514			0.04	-1.3	0.41	-1.2	0	-1.5
A_30_P01029711			0.08	-1.3	0.2	-1.3	0	-1.5
A_30_P01029702			0.23	-1.3	0.19	-1.3	0	-1.5
A_30_P01023171			0.58	-1.1	0.16	-1.3	0	-1.5
A_55_P2083426			0.12	-1.3	0.57	-1.2	0	-1.5
A_30_P01019947			0.04	-1.3	0.56	-1.2	0	-1.5
A_30_P01031360			0.01	-1.3	0.06	-1.3	0	-1.5
A_55_P1979997	NM_027283	1700026L06Rik	0.58	-1.2	0.4	-1.3	0.01	-1.5
A_55_P1989514	NR_033207	2010016I18Rik	0.05	-1.4	0.56	-1.2	0	-1.5
A_51_P439085	NM_023516	2310016C08Rik	0.15	-1.2	0.02	-1.4	0	-1.5

A_55_P2273929	AK076353	4732457N14	0.04	-1.4	0.11	-1.4	0	-1.5
A_55_P2133646	NM_199316	4922501C03Rik	0	-1.5	0.37	-1.2	0	-1.5
A_55_P2002449	XM_001473799	4932422M17Rik	0.08	-1.4	0.69	-1.2	0.01	-1.5
A_55_P1996927	NR_033123	4933409K07Rik	0.02	-1.3	0.42	-1.2	0	-1.5
A_55_P2316612	AK020106	6720407P12Rik	0.26	-1.3	0.99	-1	0.02	-1.5
A_55_P2255628	AK020413	9430013L14Rik	0.03	-1.2	0.63	-1.1	0	-1.5
A_55_P2235387	AK079318	9630020I17Rik	0	-1.4	0.56	-1.1	0	-1.5
A_55_P2116650	NR_002860	A130040M12Rik	0.24	-1.3	0.98	-1	0.01	-1.5
A_66_P108380	NM_172447	A330021E22Rik	0.21	-1.3	0.5	-1.2	0.01	-1.5
A_55_P2381538	AK031601	A430108E01Rik	0.01	-1.3	0.52	-1.1	0	-1.5
A_55_P1985623	NM_029600	Abcc3	0.12	-1.2	0.04	-1.4	0	-1.5
A_55_P2073935	NM_013790	Abcc5	0.11	-1.3	0.09	-1.4	0.01	-1.5
A_55_P2165539	NM_009637	Aebp2	0.03	-1.5	0.77	-1.1	0	-1.5
A_55_P2174743	NM_018747	Akap7	0.07	-1.3	0.39	-1.2	0	-1.5
A_55_P2012999	NM_170728	Ank3	0.3	-1.2	0.2	-1.3	0	-1.5
A_52_P97572	NM_011784	Aplnr	1	1	0.93	-1.1	0.03	-1.5
A_55_P2057528	NM_025404	Arl4d	0.02	-1.5	0.14	-1.4	0.01	-1.5
A_55_P2020612	NM_025404	Arl4d	0.12	-1.3	0.2	-1.3	0.01	-1.5
A_51_P111612	NM_001042592	Arrdc4	0	-1.5	0	-1.6	0	-1.5
A_51_P484842	NM_007493	Asgr2	0.06	-1.2	0	-1.4	0	-1.5
A_52_P97670	NM_001099628	Atad2b	0.03	-1.3	0.4	-1.2	0	-1.5
A_52_P194316	NM_001128094	Atp13a3	0.02	-1.5	0.76	-1.1	0.01	-1.5
A_55_P2274378	AK035112	AW549542	0.14	-1.4	0.49	-1.3	0.04	-1.5
A_55_P2249897	AK081173	B930096F20Rik	0.03	-1.3	0.14	-1.2	0	-1.5
A_51_P371174	NM_013863	Bag3	0.25	-1.3	0.2	-1.4	0.03	-1.5
A_55_P2360427	NM_001166581	BC005561	0.03	-1.3	0.06	-1.3	0	-1.5
A_52_P161495	NM_009744	Bcl6	0.27	-1.3	0.65	-1.2	0.01	-1.5
A_55_P2062437	NM_001163175	Begain	0.89	-1.1	0.93	-1.1	0.03	-1.5
A_55_P1966583	NM_177772	Bpil2	0.11	-1.3	0.58	-1.2	0.01	-1.5
A_55_P2040539	NM_172618	Btbd9	0.02	-1.3	0.06	-1.3	0	-1.5
A_55_P2270412	AK049070	C230096K16Rik	0	-1.4	0.37	-1.1	0	-1.5
A_55_P1985259	NM_001017985	C2cd3	0.05	-1.3	0.42	-1.2	0	-1.5
A_55_P2016739	NM_198957	C430048L16Rik	0	-1.3	0.02	-1.3	0	-1.5
A_55_P2126662	NM_007616	Cav1	0.02	-1.3	0.01	-1.5	0	-1.5
A_55_P2123069	NM_001109873	Cbfa2t3	0.03	-1.5	0.3	-1.3	0.01	-1.5
A_55_P1955821	NM_011335	Ccl21b	0.91	-1.1	0.43	-1.3	0.02	-1.5
A_52_P590474	NM_019937	Ccnl1	0.02	-1.3	0.21	-1.2	0	-1.5
A_55_P2166501	NM_009851	Cd44	0	-1.4	0	-1.3	0	-1.5
A_55_P2180415	NM_001110320	Cd72	0.96	-1	0.58	1.2	0.02	-1.5
A_55_P2009918	NM_007735	Col4a4	0	-1.5	0.11	-1.4	0.01	-1.5
A_55_P1962603	NM_009946	Cplx2	0.05	-1.4	0.58	-1.2	0.01	-1.5
A_55_P2067960	NM_009946	Cplx2	0.07	-1.4	0.68	-1.2	0	-1.5
A_55_P2044413	NM_019494	Cxcl11	0.1	-1.3	0.5	-1.2	0	-1.5
A_55_P2117959	NM_007823	Cyp4b1	0.32	-1.2	0.4	-1.3	0.01	-1.5
A_55_P2230968	AK086771	D930050J11	0.06	-1.3	0.92	-1.1	0.01	-1.5
A_55_P2112967	NM_007858	Diap1	0.01	-1.4	0.2	-1.3	0	-1.5
A_55_P2150389	NM_030046	Dnajc21	0.07	-1.2	0.19	-1.2	0	-1.5
A_55_P2031167	NM_010107	Efna1	0.21	-1.2	0.14	-1.3	0	-1.5
A_55_P2033362	NM_010118	Egr2	0.09	-1.4	0.06	-1.5	0.03	-1.5
A_51_P484054	NM_010172	F7	0.69	-1.1	0.12	-1.4	0.01	-1.5
A_55_P2045040	NM_010172	F7	0.17	-1.2	0.12	-1.3	0	-1.5
A_55_P2054280	NM_001126046	Fam178b	0.02	-1.2	0.03	-1.2	0	-1.5
A_52_P493091	NM_008031	Fmr1	0.08	-1.2	0.8	-1.1	0	-1.5
A_55_P2030771	NM_019406	Fnbp1	0.01	-1.5	0.31	-1.3	0	-1.5
A_51_P153423	NM_001081416	Fndc1	0.21	-1.3	0.03	-1.6	0.03	-1.5

A_55_P2006035	NM_030166	Galntl2	0.55	-1.2	0.36	-1.3	0.02	-1.5
A_55_P2064676	AK171905	Gas2l3	0.5	-1.2	0.58	-1.3	0.04	-1.5
A_55_P2114697	NM_008103	Gcm1	0.02	-1.5	0.68	-1.2	0.01	-1.5
A_55_P2243431	BC052902	Gdap10	0.17	-1.2	0.93	-1	0	-1.5
A_55_P2175486	NM_001098269	Gm10375	0.23	-1.3	0.85	-1.1	0.01	-1.5
A_55_P2049262	XM_001477897	Gm16525	0.55	-1.1	0.86	-1.1	0	-1.5
A_55_P2130905	XM_001471976	Gm1983	0.06	-1.3	0.25	-1.2	0	-1.5
A_55_P2003309	XM_001472364	Gm2073	0.23	-1.4	0.79	-1.2	0.03	-1.5
A_55_P2156556	XM_001472797	Gm2212	0.15	-1.3	0.33	-1.3	0	-1.5
A_55_P2148400	XM_001472970	Gm2264	0.02	-1.4	0.43	-1.2	0.01	-1.5
A_55_P2040341	XM_001473039	Gm2285	0.03	-1.3	0.23	-1.2	0	-1.5
A_55_P1980621	XM_001473665	Gm2459	0.18	-1.3	0.73	-1.2	0.01	-1.5
A_55_P2152188	XM_001473965	Gm2556	0	-1.4	0.43	-1.2	0	-1.5
A_55_P1962602	XM_001474429	Gm2690	0.11	-1.4	0.61	-1.2	0.02	-1.5
A_52_P135873	XM_001475453	Gm2744	0.24	-1.2	0.91	-1.1	0	-1.5
A_51_P186798	XM_001475285	Gm2881	0.22	-1.3	0.88	-1.1	0.04	-1.5
A_55_P2092156	XM_001475084	Gm2911	0.06	-1.4	0.75	-1.1	0	-1.5
A_55_P2048867	XM_001475122	Gm2922	0.07	-1.4	0.9	-1.1	0.01	-1.5
A_55_P2075731	XM_001475177	Gm2943	0.02	-1.5	0.74	-1.1	0	-1.5
A_55_P1961760	XM_001476058	Gm3237	0.1	-1.4	0.83	-1.1	0.01	-1.5
A_55_P2134712	XM_001478003	Gm3781	0.27	-1.2	0.72	-1.1	0	-1.5
A_55_P1989765	XM_001478600	Gm3951	0.04	-1.5	0.69	-1.2	0.01	-1.5
A_55_P1993049	XM_001478943	Gm4076	0.78	-1.1	0.6	-1.2	0.01	-1.5
A_55_P2108275	XM_001480646	Gm4578	0.03	-1.4	0.37	-1.2	0	-1.5
A_55_P2008870	XM_141816	Gm4910	0.09	-1.2	0.28	-1.2	0	-1.5
A_55_P1971159	XM_921759	Gm5469	0.1	-1.2	0.72	-1.1	0	-1.5
A_55_P1991783	XM_001477578	Gm7149	0	-1.6	0.61	-1.2	0	-1.5
A_55_P1993483	XM_974153	Gm7462	0.2	-1.3	0.69	-1.2	0.02	-1.5
A_55_P2046728	XM_981891	Gm7792	0.17	-1.2	0.4	-1.2	0	-1.5
A_66_P127262	XM_985872	Gm8799	0	-1.5	0	-1.3	0	-1.5
A_55_P2101944	XM_001479912	Gm9444	0.01	-1.4	0.69	-1.1	0.01	-1.5
A_55_P2426941	BC040234	Gprin3	0.08	-1.3	0.05	-1.4	0	-1.5
A_55_P1956302	NM_001143689	H2-gs10	0.39	-1.2	0.83	-1.1	0.02	-1.5
A_66_P108019	NM_144835	Heatr1	0.2	-1.2	0.02	-1.4	0	-1.5
A_55_P2025153	NM_010439	Hmgb1	0.1	-1.3	0.69	-1.2	0	-1.5
A_55_P2058628	NM_198090	Hnrnpa3	0.41	-1.3	0.97	-1	0.04	-1.5
A_55_P2020443	NM_198090	Hnrnpa3	0.15	-1.4	0.93	1.1	0.02	-1.5
A_51_P347452	NM_028242	Htatsf1	0.1	-1.2	0.36	-1.2	0	-1.5
A_66_P139546	NM_008344	Igfbp6	0.03	-1.4	0.18	-1.3	0	-1.5
A_55_P2017709	NM_033608	Igsf9	0.48	-1.2	0.77	-1.1	0.01	-1.5
A_55_P2092492	NM_001161842	Il18r1	0.07	-1.3	0.01	-1.5	0	-1.5
A_55_P1957593	NM_023547	Ino80b	0.04	-1.3	0.54	-1.2	0	-1.5
A_66_P103271	NM_001164598	Irf2bp2	0.1	-1.3	0.39	-1.2	0	-1.5
A_55_P2125557	NM_010574	Irx2	0.06	-1.3	0.05	-1.3	0	-1.5
A_52_P518922	NM_001033228	Itga1	0.28	-1.3	0.26	-1.4	0.01	-1.5
A_51_P295034	NM_010915	Klk1b4	0.56	-1.2	0.53	-1.3	0.03	-1.5
A_55_P2170350	NM_053152	Klra22	0.03	-1.4	0.03	-1.4	0	-1.5
A_55_P1979147	NM_001159904	Klrb1c	0.74	-1.1	0.11	-1.3	0	-1.5
A_55_P1988844	NM_016970	Klrg1	0.03	-1.4	0.01	-1.5	0	-1.5
A_55_P2167999	NM_010700	Ldlr	0.03	-1.3	0.21	-1.2	0	-1.5
A_55_P2177910	NM_010704	Lepr	0.57	-1.2	0.84	-1.1	0.03	-1.5
A_55_P2072041	XM_001472087	LOC100038980	0.25	-1.3	0.85	-1.1	0.01	-1.5
A_55_P2138739	XM_001473119	LOC100039570	0.02	-1.5	0.52	-1.2	0	-1.5
A_55_P2062340	XM_001480267	LOC100044039	0.51	-1.2	0.91	-1.1	0.04	-1.5
A_55_P1981455	XM_001472240	LOC100044430	0	-1.5	0.04	-1.4	0	-1.5

A_55_P2020035	XM_001473697	LOC100045113	0.05	-1.4	0.73	-1.2	0.01	-1.5
A_55_P2078831	XM_001473861	LOC100045156	0.08	-1.3	0.88	-1.1	0	-1.5
A_55_P2132812	XM_001474453	LOC100045801	0.4	-1.3	0.79	-1.2	0.03	-1.5
A_55_P2083149	XM_001476298	LOC100046485	0.23	-1.2	1	-1	0	-1.5
A_55_P2054998	XM_001476461	LOC100046838	0.04	-1.3	0.85	-1.1	0	-1.5
A_55_P1988260	XM_001479382	LOC100048847	0	-1.5	0.05	-1.3	0	-1.5
A_55_P2114187	XM_884904	LOC620515	0.94	-1.1	0.04	-2.5	0.25	-1.5
A_55_P2012161	XM_001476592	LOC624231	0.04	-1.3	0.26	-1.2	0	-1.5
A_55_P2108486	XM_903408	LOC630284	0.05	-1.3	0.38	-1.2	0	-1.5
A_55_P2129407	XM_908705	LOC634091	0.02	-1.5	0.55	-1.2	0	-1.5
A_52_P547187	NM_138667	Map3k7ip2	0.33	-1.2	0.24	-1.2	0	-1.5
A_51_P158814	AK081212	Marveld1	0.54	-1.2	0.81	-1.1	0	-1.5
A_55_P2095047	NM_145569	Mat2a	0.19	-1.2	0.31	-1.2	0	-1.5
A_51_P484111	NM_016762	Matn2	0.48	-1.2	0.18	-1.4	0.01	-1.5
A_55_P2454099	NM_023799	Mgea5	0.13	-1.3	0.44	-1.3	0.01	-1.5
A_55_P2347976	NR_029382	Mirhg1	0.01	-1.4	0.05	-1.3	0	-1.5
A_55_P2136526	NM_001033276	Mll2	0	-1.5	0.06	-1.3	0	-1.5
A_55_P1988531	NM_001161775	Myh11	0.48	-1.2	0.4	-1.3	0.01	-1.5
A_55_P2077866	NM_010918	Nktr	0.01	-1.4	0.27	-1.3	0	-1.5
A_55_P2117243	AK088923	Npm1	0.11	-1.3	0.71	-1.2	0.01	-1.5
A_52_P565940	NM_008739	Nsd1	0.03	-1.5	0.57	-1.2	0	-1.5
A_51_P254262	NM_130858	Nxph3	0	-1.6	0	-1.9	0	-1.5
A_55_P2134387	NM_020289	Olfr544	0	-1.4	0.23	-1.2	0	-1.5
A_55_P2183672	NM_018858	Pebp1	0	-1.6	0.07	-1.3	0	-1.5
A_51_P327778	NM_011070	Pfdn2	0.18	-1.3	0.97	-1	0.01	-1.5
A_55_P2126627	NM_001081409	Phf20l1	0.03	-1.4	0.54	-1.2	0.01	-1.5
A_51_P189746	NM_145478	Pim3	0.72	-1.1	0.82	-1.1	0	-1.5
A_55_P2212908	AK037585	Pon2	0.08	-1.3	0.06	-1.4	0	-1.5
A_55_P2132472	NM_013830	Prpf4b	0.02	-1.3	0.03	-1.3	0	-1.5
A_55_P2171623	NM_001077363	Ptbp1	0.27	-1.2	0.14	-1.3	0	-1.5
A_52_P28651	NM_021424	Pvrl1	0.19	-1.3	0.38	-1.2	0	-1.5
A_55_P2113078	NM_009047	Rem1	0	-1.4	0	-1.5	0	-1.5
A_55_P2100241	NR_003278	Rn18s	0	-1.5	0.74	-1.2	0.01	-1.5
A_51_P111164	NM_172612	Rnd1	0.13	-1.4	0.39	-1.3	0.01	-1.5
A_55_P2159934	NM_025786	Rnf186	0.54	-1.2	0.6	-1.2	0	-1.5
A_55_P1987290	NR_004413	Rnu1b6	0.14	-1.3	0.24	-1.3	0.01	-1.5
A_52_P134023	NM_153100	Rtp3	0.2	-1.2	0.06	-1.4	0	-1.5
A_55_P2031496	NM_027530	Rufy3	0.01	-1.4	0.04	-1.3	0	-1.5
A_55_P2075070	NM_053190	S1pr5	0.13	-1.3	0.03	-1.5	0	-1.5
A_55_P2060429	NM_020052	Scube2	0.05	-1.5	0.91	-1.1	0.02	-1.5
A_55_P2021011	NM_030207	Sfi1	0.08	-1.5	0.96	-1.1	0.02	-1.5
A_51_P173678	NM_029415	Slc10a6	0.2	-1.4	0.27	-1.4	0.04	-1.5
A_51_P112762	NM_017391	Slc5a3	0.17	-1.3	0.22	-1.3	0.01	-1.5
A_55_P2127592	NM_001085440	Smcr8	0.02	-1.3	0.12	-1.3	0	-1.5
A_55_P2079388	NR_002905	Snora74a	0.1	-1.3	0.34	-1.3	0.01	-1.5
A_55_P2137121	NM_001013817	Sp140	0.74	-1.1	0.93	-1.1	0	-1.5
A_55_P2183498	NM_025668	Spcs2	0	-1.5	0.12	-1.3	0	-1.5
A_52_P291971	NM_025303	Stau2	0.07	-1.3	0.07	-1.3	0	-1.5
A_55_P2144456	NM_001167884	Suv420h1	0	-1.4	0.17	-1.2	0	-1.5
A_51_P240693	NM_027410	Tecpr1	0.02	-1.4	0.03	-1.4	0	-1.5
A_51_P475995	NM_178060	Thra	0.11	-1.2	0.05	-1.2	0	-1.5
A_55_P2069425	NM_133923	Ttll3	0.66	-1.1	0.44	-1.3	0.02	-1.5
A_55_P1994492	NM_001033173	Usp31	0.05	-1.3	0.4	-1.2	0	-1.5
A_55_P2212027	NM_011682	Utrn	0.07	-1.4	0.57	-1.2	0.01	-1.5
A_55_P2131520	NM_177354	Vash1	0.05	-1.4	0.73	-1.1	0	-1.5

A_55_P2179604	NM_177354	Vash1	0.1	-1.4	0.68	-1.2	0	-1.5
A_66_P100937	XM_001487796	Zfp33b	0.69	-1.1	0.4	-1.2	0	-1.5
A_55_P2173373	NM_001024846	Zfp62	0.11	-1.4	0.38	-1.3	0.02	-1.5
A_51_P108020	NM_001029929	Zmynd15	0.02	-1.4	0	-1.6	0	-1.5
A_55_P1968085			0.05	-1.5	0.8	-1.2	0.01	-1.6
A_30_P01029719			0.02	-1.5	0.67	-1.2	0.01	-1.6
A_55_P2116149			0.01	-1.5	0.55	-1.2	0	-1.6
A_55_P2021476	XM_001474821		0.01	-1.5	0.56	-1.2	0	-1.6
A_30_P01029062			0	-1.5	0.01	-1.4	0	-1.6
A_30_P01025909			0	-1.5	0.02	-1.3	0	-1.6
A_30_P01028277			0	-1.5	0.11	-1.3	0	-1.6
A_30_P01026605			0	-1.5	0.67	-1.2	0	-1.6
A_55_P2105517	XM_001473956		0	-1.5	0	-1.6	0	-1.6
A_55_P2072315	AK133410		0	-1.5	0.12	-1.4	0	-1.6
A_30_P01025354			0	-1.5	0.11	-1.3	0	-1.6
A_30_P01019829			0.01	-1.6	0.49	-1.2	0	-1.6
A_30_P01026040			0	-1.6	0.21	-1.3	0	-1.6
A_30_P01025227			0	-1.6	0.55	-1.2	0	-1.6
A_30_P01026230			0	-1.6	0.08	-1.3	0	-1.6
A_30_P01020570			0	-1.6	0.26	-1.3	0	-1.6
A_30_P01021078			0.01	-1.7	0.5	-1.2	0.01	-1.6
A_30_P01025536			0	-1.7	0.14	-1.3	0	-1.6
A_52_P311031	AK016943		0	-1.7	0.18	-1.3	0	-1.6
A_30_P01031627			0.01	-1.4	0	-1.5	0	-1.6
A_30_P01026069			0.12	-1.5	0.28	-1.4	0.03	-1.6
A_30_P01025522			0.3	-1.4	0.75	-1.2	0.03	-1.6
A_30_P01030969			0.2	-1.4	0.85	-1.2	0.03	-1.6
A_30_P01031525			0.21	-1.4	0.87	-1.1	0.02	-1.6
A_30_P01026028			0.29	-1.3	0.98	-1	0.01	-1.6
A_51_P102257	AK053112		0.37	-1.2	0.21	-1.4	0.01	-1.6
A_30_P01024724			0.14	-1.4	0.51	-1.3	0.01	-1.6
A_55_P1984253	BC145649		0.04	-1.4	0.21	-1.3	0	-1.6
A_55_P1982075			0.07	-1.3	0.22	-1.3	0	-1.6
A_55_P2116235			0.04	-1.2	0.13	-1.2	0	-1.6
A_30_P01027180			0.02	-1.4	0.58	-1.2	0	-1.6
A_55_P2168823			0.04	-1.3	0.01	-1.4	0	-1.6
A_30_P01026332			0	-1.4	0.1	-1.2	0	-1.6
A_55_P2092310	AK195427		0.13	-1.2	0.01	-1.4	0	-1.6
A_30_P01023029			0	-1.4	0.31	-1.2	0	-1.6
A_30_P01027700			0	-1.4	0.19	-1.2	0	-1.6
A_30_P01023337			0.01	-1.4	0.73	-1.1	0	-1.6
A_30_P01032304			0.7	-1.1	0.97	-1	0	-1.6
A_66_P123735	AK143547		0.15	-1.2	0.27	-1.2	0	-1.6
A_55_P1984416			0.41	-1.2	0.21	-1.3	0	-1.6
A_30_P01030704			0.44	-1.2	0.73	-1.2	0	-1.6
A_55_P2003199	AK141316		0.03	-1.3	0.02	-1.4	0	-1.6
A_55_P2023912			0.03	-1.3	0.03	-1.3	0	-1.6
A_55_P2075294			0.1	-1.3	0.54	-1.2	0	-1.6
A_55_P2069530			0.07	-1.4	0.2	-1.3	0	-1.6
A_30_P01029746			0.03	-1.3	0.2	-1.2	0	-1.6
A_55_P2105436	AK144920		0.01	-1.4	0.31	-1.2	0	-1.6
A_55_P2064025	XM_001474990		0.09	-1.3	0.64	-1.1	0	-1.6
A_30_P01028904			0.03	-1.3	0.18	-1.2	0	-1.6
A_30_P01032449			0.01	-1.4	0.75	-1.1	0	-1.6
A_55_P2058783	NM_023516	2310016C08Rik	0.09	-1.2	0	-1.5	0	-1.6

A_55_P2281624	AK142273	2610034E01Rik	0	-1.4	0.26	-1.2	0	-1.6
A_55_P2178137	NM_011889	41885.0	0	-1.9	0	-1.7	0	-1.6
A_55_P2230506	AK016424	4931402H11Rik	0.08	-1.4	0.35	-1.3	0	-1.6
A_51_P474169	NM_144883	5430407P10Rik	0.04	-1.3	0.02	-1.4	0	-1.6
A_55_P2192729	AK018045	5830469G19Rik	0	-1.4	0.47	-1.1	0	-1.6
A_55_P2279035	AK018519	9030419F21Rik	0.07	-1.3	0.26	-1.3	0	-1.6
A_52_P146403	NM_029953	9130221D24Rik	0.41	-1.2	0.88	-1.1	0	-1.6
A_55_P2249556	AK042310	A630081D01Rik	0.12	-1.3	0.49	-1.2	0	-1.6
A_55_P2038358	NM_012006	Acot1	0.45	-1.2	0.28	-1.3	0.01	-1.6
A_52_P162509	NM_021414	Ahcyl2	0.08	-1.2	0	-1.6	0	-1.6
A_55_P2153620	NM_001039959	Ahnak	0	-1.6	0.42	-1.2	0	-1.6
A_55_P2018176	AK039146	AI504432	0.02	-1.4	0.12	-1.3	0	-1.6
A_52_P625171	NM_133723	Asph	0.01	-1.4	0.46	-1.2	0	-1.6
A_55_P1990879	NM_016812	Banp	0	-1.4	0	-1.4	0	-1.6
A_65_P01643	NM_001081345	Chd2	0.1	-1.3	0.76	-1.1	0	-1.6
A_55_P2080956	NM_013490	Chka	0	-1.4	0.02	-1.3	0	-1.6
A_55_P2061620	NM_023850	Chst1	0.15	-1.3	0.13	-1.3	0	-1.6
A_51_P304397	NM_027468	Cpm	0.09	-1.3	0.18	-1.3	0	-1.6
A_51_P196925	NM_009142	Cx3cl1	0.15	-1.2	0.11	-1.3	0	-1.6
A_51_P116813	NM_007809	Cyp17a1	0.03	-1.6	0.34	-1.4	0.01	-1.6
A_55_P2313163	AK156477	D10Bwg1070e	0.07	-1.3	0.84	-1.1	0	-1.6
A_55_P1961761	NM_007831	Dcc	0.02	-1.5	0.66	-1.2	0	-1.6
A_51_P306017	NM_007865	Dll1	0.22	-1.3	0.12	-1.5	0.01	-1.6
A_55_P2156905	XM_991400	EG667601	0.16	-1.4	0.77	-1.2	0.02	-1.6
A_55_P2005956	NM_010115	Egfbp2	0	-1.4	0	-1.7	0	-1.6
A_52_P344376	NM_013506	Eif4a2	0.07	-1.4	0.43	-1.3	0	-1.6
A_55_P1982454	NM_007945	Eps8	0.91	-1	0.23	-1.3	0	-1.6
A_55_P2136752	NM_029972	Ermn	0.04	-1.5	0.64	-1.2	0	-1.6
A_52_P437850	NM_024244	Fam13c	0.04	-1.5	0.44	-1.2	0	-1.6
A_55_P2029902	NM_181584	Gab3	0.02	-1.5	0.52	-1.2	0	-1.6
A_52_P233441	NM_008090	Gata2	0	-1.4	0	-1.4	0	-1.6
A_51_P461319	NM_025961	Gatm	0.24	-1.4	0.59	-1.3	0.01	-1.6
A_55_P1989813	NM_008103	Gcm1	0.22	-1.4	0.8	-1.2	0.01	-1.6
A_55_P2039196	NM_020014	Gfra4	0	-1.5	0.1	-1.3	0	-1.6
A_55_P2092831	XM_001472097	Gm2008	0.01	-1.5	0.48	-1.2	0	-1.6
A_55_P2143516	XM_001473058	Gm2291	0	-1.5	0.54	-1.2	0	-1.6
A_55_P2021398	XM_001473399	Gm2393	0.02	-1.5	0.63	-1.2	0	-1.6
A_55_P2035038	XM_001473590	Gm2437	0.04	-1.4	0.76	-1.1	0	-1.6
A_55_P2068248	XM_001473755	Gm2488	0	-1.5	0.51	-1.2	0	-1.6
A_55_P2096127	XM_001473875	Gm2527	0	-1.5	0.47	-1.2	0	-1.6
A_55_P2096121	XM_001473876	Gm2528	0.03	-1.4	0.6	-1.2	0	-1.6
A_55_P2180445	XM_001474578	Gm2742	0.08	-1.4	0.62	-1.2	0	-1.6
A_55_P2133624	XM_001475193	Gm2891	0	-1.6	0.05	-1.4	0	-1.6
A_55_P1960479	XM_001475709	Gm3114	0.02	-1.5	0.64	-1.2	0.01	-1.6
A_55_P2047620	XM_001475740	Gm3124	0	-1.5	0.48	-1.2	0	-1.6
A_55_P2070766	XM_001476070	Gm3241	0.02	-1.5	0.7	-1.2	0	-1.6
A_55_P1974780	XM_001476301	Gm3306	0.02	-1.5	0.49	-1.2	0	-1.6
A_55_P2019833	XM_001477941	Gm3790	0.02	-1.5	0.58	-1.2	0	-1.6
A_55_P2005720	XM_001479578	Gm4253	0.21	-1.4	0.71	-1.2	0.02	-1.6
A_55_P2086954	XM_001479991	Gm4372	0.02	-1.5	0.56	-1.2	0	-1.6
A_55_P2137286	XM_001479609	Gm4408	0.08	-1.4	0.96	-1	0	-1.6
A_55_P2058467	XM_001479915	Gm4415	0.04	-1.4	0.34	-1.2	0	-1.6
A_55_P2148896	XM_001480348	Gm4492	0.24	-1.3	0.5	-1.3	0.01	-1.6
A_55_P2009042	XM_001481023	Gm4635	0.02	-1.4	0.2	-1.3	0	-1.6
A_55_P2126557	XM_619973	Gm5858	0	-1.5	0.04	-1.4	0	-1.6

A_55_P2040775	XM_900336	Gm6837	0.13	-1.4	0.72	-1.2	0.01	-1.6
A_55_P2040774	XM_900336	Gm6837	0.05	-1.4	0.69	-1.2	0	-1.6
A_55_P2077501	XM_901095	Gm6940	0.02	-1.5	0.64	-1.2	0	-1.6
A_55_P2077497	XM_001476406	Gm6940	0.01	-1.5	0.58	-1.2	0	-1.6
A_55_P2071581	XM_978127	Gm7710	0.32	-1.3	0.78	-1.2	0.02	-1.6
A_55_P1957867	XM_001472293	Gm8090	0.22	-1.4	0.83	-1.2	0.03	-1.6
A_55_P1957871	XM_983688	Gm8090	0.06	-1.5	0.77	-1.2	0.01	-1.6
A_55_P2121760	XM_001476650	Gm9147	0.05	-1.4	0.59	-1.2	0	-1.6
A_55_P2028847	XM_001476302	Gm9468	0.02	-1.5	0.62	-1.2	0	-1.6
A_52_P655663	NM_010376	H13	0.19	-1.2	0.13	-1.3	0	-1.6
A_52_P669922	NM_032541	Hamp	0.25	1.4	0.92	-1.1	0.02	-1.6
A_55_P2096422	NM_008381	Inhbb	0.08	-1.3	0.08	-1.4	0	-1.6
A_55_P2001494	NM_013598	Kitl	0.04	-1.3	0.13	-1.3	0	-1.6
A_55_P2004541	NM_001110323	Klra7	0.05	-1.4	0.04	-1.5	0	-1.6
A_55_P1981830	XM_001475952	LOC100041203	0.01	-1.5	0.51	-1.2	0	-1.6
A_55_P2107357	NR_004442	LOC100042049	0.09	-1.2	0.16	-1.2	0	-1.6
A_55_P1967591	XM_001472709	LOC100044615	0.01	-1.5	0.48	-1.2	0	-1.6
A_55_P1959639	XM_001473594	LOC100045061	0.05	-1.4	0.7	-1.2	0	-1.6
A_55_P2027337	XM_001473753	LOC100045145	0	-1.5	0.16	-1.3	0	-1.6
A_55_P2294037	XM_001475407	LOC100045958	0	-1.6	0.64	-1.2	0	-1.6
A_55_P2004168	XM_001476023	LOC100046325	0.07	-1.4	0.69	-1.2	0.01	-1.6
A_55_P2044380	XM_001476392	LOC100046544	0.08	-1.3	0.25	-1.3	0	-1.6
A_55_P1978726	XM_001477052	LOC100046908	0.15	-1.3	0.68	-1.2	0	-1.6
A_55_P1962699	XM_001477233	LOC100047021	0.01	-1.5	0.3	-1.3	0	-1.6
A_55_P2139430	XM_001479756	LOC100048207	0	-1.6	0.12	-1.3	0	-1.6
A_55_P1964332	XM_001479828	LOC100048239	0.08	-1.4	0.58	-1.2	0	-1.6
A_55_P2048660	XM_908118	LOC633654	0	-1.5	0.02	-1.4	0	-1.6
A_55_P2018307	XM_001005025	LOC677576	0	-1.6	0.2	-1.3	0	-1.6
A_55_P2081388	NM_008575	Mdm4	0.02	-1.7	0.44	-1.3	0.01	-1.6
A_55_P2062793	NM_008546	Mfap2	0.12	-1.3	0.13	-1.3	0	-1.6
A_55_P2091350	AK140187	ND4L	0.19	-1.3	0.67	-1.2	0.01	-1.6
A_55_P2035320	NM_017373	Nfil3	0.16	-1.3	0	-2	0.01	-1.6
A_55_P2158522	NM_183355	Pbx1	0	-1.5	0.01	-1.3	0	-1.6
A_55_P2009449	NM_175498	Pnma2	0.01	-1.5	0.46	-1.2	0	-1.6
A_55_P2079064	XM_001471675	Ppnr	0.03	-1.2	0.11	-1.2	0	-1.6
A_55_P2000039	NM_011073	Prf1	0.18	-1.2	0.01	-1.5	0	-1.6
A_55_P1981829	NM_001004193	Rhox8	0.01	-1.5	0.59	-1.2	0	-1.6
A_55_P2009576	NR_004432	Rnu12	0.25	-1.3	0.43	-1.3	0.01	-1.6
A_55_P2020361	NM_197945	RP23-100C5.8	0.14	-1.4	0.79	-1.2	0	-1.6
A_55_P2159026	NM_026517	Rpl22l1	0.09	-1.2	0.21	-1.2	0	-1.6
A_55_P2144436	NM_010925	Rrp1	0.05	-1.3	0.65	-1.1	0	-1.6
A_55_P2146185	NM_011302	Rs1	0.08	-1.5	0.8	-1.2	0.01	-1.6
A_66_P112065	AK142268	Sertad2	0.13	-1.2	0.1	-1.3	0	-1.6
A_51_P421958	NM_144792	Sgms1	0.14	-1.3	0.69	-1.2	0	-1.6
A_55_P1968763	NM_199022	Shc4	0.11	-1.4	0.78	-1.2	0.01	-1.6
A_55_P2043277	NM_145581	Siglec5	0.59	-1.2	0.01	-2.1	0.05	-1.6
A_55_P1965030	NM_001003915	Slc5a12	0	-1.5	0	-1.6	0	-1.6
A_51_P299632	NM_027641	Spef1	0.88	-1.1	0.71	-1.2	0.02	-1.6
A_55_P1996456	NM_016799	Srrm1	0.03	-2	0.01	-2.2	0.09	-1.6
A_55_P2026125	NM_175229	Srrm2	0	-1.5	0.06	-1.3	0	-1.6
A_55_P2118609	NM_011371	St6galnac1	0.08	-1.4	0.69	-1.2	0.01	-1.6
A_55_P2005873	NM_146224	Suhw4	0.02	-1.4	0.49	-1.2	0	-1.6
A_51_P318830	NM_018803	Syt10	0.04	-1.5	0.72	-1.2	0	-1.6
A_51_P501364	NM_019507	Tbx21	0.1	-1.3	0.05	-1.5	0	-1.6
A_55_P1958245	NM_011552	Tcof1	0.01	-1.5	0.47	-1.2	0	-1.6

A_55_P1989865	NM_146153	Thrap3	0.01	-1.5	0.44	-1.2	0	-1.6
A_51_P485458	NM_001005506	Txlna	0.09	-1.3	0.06	-1.4	0	-1.6
A_51_P200610	NM_198886	Zbtb12	0.15	-1.4	0.47	-1.3	0	-1.6
A_55_P2025820	XM_001480287		0.01	-1.5	0.2	-1.3	0	-1.7
A_30_P01032760			0	-1.5	0.06	-1.3	0	-1.7
A_30_P01028770			0	-1.5	0.01	-1.5	0	-1.7
A_55_P2183668			0	-1.5	0.3	-1.2	0	-1.7
A_55_P2064171			0.01	-1.6	0.48	-1.2	0	-1.7
A_30_P01020465			0	-1.6	0.18	-1.3	0	-1.7
A_30_P01032670			0	-1.6	0.01	-1.4	0	-1.7
A_30_P01030106			0	-1.6	0.15	-1.3	0	-1.7
A_30_P01022751			0	-1.6	0.06	-1.3	0	-1.7
A_30_P01022343			0	-1.7	0.06	-1.4	0	-1.7
A_30_P01020476			0	-1.7	0.04	-1.4	0	-1.7
A_30_P01024297			0	-1.7	0	-1.4	0	-1.7
A_30_P01030436			0.04	-1.3	0.07	-1.3	0	-1.7
A_55_P2109564	AK039146		0	-1.4	0.14	-1.2	0	-1.7
A_55_P2085835	NM_001033304	5330417C22Rik	0.7	-1.2	0.77	-1.2	0.01	-1.7
A_55_P2105321	NM_023190	Acin1	0	-1.5	0.14	-1.3	0	-1.7
A_55_P2266977	NM_021414	Ahcyl2	0.53	-1.1	0.02	-1.5	0	-1.7
A_55_P2403769	BB498095	AI481121	0.17	-1.4	0.29	-1.4	0.01	-1.7
A_55_P1977875	NM_176849	Arglu1	0	-1.5	0	-1.5	0	-1.7
A_55_P2003823	NM_009714	Asgr1	0.98	-1	0.53	-1.2	0	-1.7
A_55_P2325378	AK039484	AW046287	0.51	-1.3	1	1	0.03	-1.7
A_55_P2033215	NM_013877	Cabp5	0.08	-1.5	0.75	-1.2	0	-1.7
A_51_P509573	NM_013652	Ccl4	0	-1.6	0.01	-1.6	0	-1.7
A_55_P2018061	NM_133238	Cd209a	0.84	-1.1	0.14	-1.3	0	-1.7
A_51_P200667	NM_053155	Clmn	0.22	-1.2	0.37	-1.2	0	-1.7
A_55_P2143837	NM_007735	Col4a4	0	-1.7	0.3	-1.2	0	-1.7
A_55_P2148402	NM_009946	Cplx2	0.14	-1.5	0.65	-1.3	0.01	-1.7
A_55_P1959973	NM_175539	Dcaf12l2	0.02	-1.5	0.75	-1.1	0	-1.7
A_51_P334104	NM_007833	Dcn	0.4	-1.3	0.01	-1.8	0.01	-1.7
A_55_P2430472	NM_011805	Dido1	0.03	-1.5	0.29	-1.3	0	-1.7
A_55_P2014978	NM_007889	Dvl3	0	-1.7	0.14	-1.4	0	-1.7
A_55_P1952618	NM_007895	Ear2	0.16	-1.5	0.11	-1.7	0.03	-1.7
A_51_P388478	NM_010110	Efnb1	0	-1.5	0	-1.5	0	-1.7
A_55_P2016105	NM_133753	Errfi1	0.85	-1.1	0.61	-1.2	0	-1.7
A_51_P283473	NM_026271	Fibin	0	-1.6	0.06	-1.4	0	-1.7
A_55_P2153783	NM_010231	Fmo1	0.04	-1.3	0.03	-1.4	0	-1.7
A_51_P483159	NM_177157	Gchfr	0.81	-1.1	0.4	-1.4	0.01	-1.7
A_55_P2154107	NM_008103	Gcm1	0.01	-1.5	0.55	-1.2	0	-1.7
A_55_P2005420	XM_001478188	Gm16439	0.45	-1.3	0.94	-1.1	0.01	-1.7
A_55_P1999818	XM_001472026	Gm2006	0	-1.7	0.15	-1.4	0	-1.7
A_52_P332788	XM_001473420	Gm2397	0.23	-1.2	0.94	-1	0	-1.7
A_55_P2068247	XM_001473755	Gm2488	0.01	-1.5	0.53	-1.2	0	-1.7
A_55_P2171788	XM_001474084	Gm2598	0.01	-1.5	0.7	-1.2	0	-1.7
A_55_P2082841	XM_001474255	Gm2658	0	-1.6	0.43	-1.2	0	-1.7
A_55_P1966383	XM_001475163	Gm2936	0.07	-1.4	0.38	-1.3	0	-1.7
A_55_P1992571	XM_001475200	Gm2952	0.26	-1.4	0.81	-1.2	0.02	-1.7
A_66_P125110	XM_001475977	Gm3006	0	-1.5	0.23	-1.3	0	-1.7
A_66_P113505	XM_001475977	Gm3006	0.01	-1.4	0.29	-1.2	0	-1.7
A_55_P2042184	XM_001476516	Gm3181	0.07	-1.5	0.56	-1.3	0.01	-1.7
A_55_P2041457	XM_001475948	Gm3195	0.01	-1.5	0.49	-1.2	0	-1.7
A_66_P140533	XM_001478088	Gm3813	0.05	-1.3	0.12	-1.3	0	-1.7
A_55_P1996953	XM_001479342	Gm4072	0.06	-1.5	0.39	-1.3	0	-1.7

A_55_P2159850	XM_001479402	Gm4191	0.05	-1.5	0.54	-1.2	0	-1.7
A_55_P1979067	XM_001481217	Gm4720	0.1	-1.3	0.55	-1.2	0	-1.7
A_65_P15809	NR_003967	Gm4759	0.01	-1.4	0.21	-1.2	0	-1.7
A_55_P2010586	AK132285	Gm6252	0.04	-1.4	0.39	-1.2	0	-1.7
A_55_P2028576	XM_001478577	Gm7057	0.19	-1.4	0.63	-1.2	0	-1.7
A_55_P1957865	XM_001472293	Gm8090	0.14	-1.5	0.72	-1.2	0.01	-1.7
A_55_P1967002	XM_001473953	Gm9441	0.1	-1.5	0.78	-1.2	0.01	-1.7
A_52_P554703	NM_183183	Gprin3	0.07	-1.4	0.06	-1.5	0	-1.7
A_55_P1982451	NM_016710	Hmgn5	0.16	-1.5	1	1	0.02	-1.7
A_52_P141161	NM_133834	Hnrnpf	0	-1.6	0.7	-1.1	0	-1.7
A_52_P12877	NM_031165	Hspa8	0.97	-1	0.04	-1.6	0	-1.7
A_51_P150710	NM_152839	Igj	0.09	-1.4	0.12	-1.5	0	-1.7
A_51_P505617	NM_008365	Il18r1	0	-1.5	0	-1.8	0	-1.7
A_55_P2408415	NR_001461	Kcnq1ot1	0	-1.5	0.02	-1.4	0	-1.7
A_55_P2151209	NM_130873	Krtap16-4	0	-1.7	0.26	-1.3	0	-1.7
A_55_P2148935	XM_001474432	LOC100040286	0.01	-1.5	0.39	-1.2	0	-1.7
A_55_P2014326	XM_001478106	LOC100047416	0.19	-1.4	0.83	-1.1	0	-1.7
A_55_P1982227	XM_001480822	LOC100048656	0.02	-1.5	0.6	-1.2	0	-1.7
A_66_P112862	NR_033146	LOC100316870	0.07	-1.4	0.35	-1.3	0	-1.7
A_51_P136521	NM_026671	Lypd2	1	-1	0.82	-1.2	0.04	-1.7
A_51_P243514	NM_001163136	Macc1	0	-1.6	0.79	-1.1	0	-1.7
A_51_P151433	NM_026554	Ncbp2	0.15	-1.3	0.04	-1.5	0	-1.7
A_55_P1960999	NM_011082	Pigr	0	-1.5	0.32	-1.2	0	-1.7
A_51_P381558	NM_133914	Rasa4	0.12	-1.2	0.05	-1.3	0	-1.7
A_55_P2172934	NM_029879	Rgs7bp	0.07	-1.5	0.78	-1.2	0	-1.7
A_51_P246066	NM_029612	Slamf9	0.05	-1.3	0.01	-1.5	0	-1.7
A_51_P210143	NM_001005510	Syne2	0.08	-1.4	0.12	-1.4	0	-1.7
A_55_P2126363	NM_011718	Wnt10b	0.01	-1.4	0.03	-1.5	0	-1.7
A_30_P01022310			0.01	-1.5	0.17	-1.4	0	-1.8
A_55_P1960148	XM_001472091		0.05	-1.8	0.02	-2.1	0.01	-1.8
A_30_P01032942			0	-1.8	0.18	-1.4	0	-1.8
A_30_P01020935			0	-1.8	0.2	-1.3	0	-1.8
A_52_P18596			0.43	-1.4	0.95	1.1	0.03	-1.8
A_66_P131433			0	-1.4	0	-1.4	0	-1.8
A_30_P01019583			0.13	-1.4	0.96	-1.1	0	-1.8
A_30_P01020677			0.18	-1.4	0.99	1	0	-1.8
A_30_P01022120			0.28	-1.4	0.26	-1.4	0	-1.8
A_55_P2098275	XM_001474884	5730416O20Rik	0.13	-1.4	0.11	-1.5	0	-1.8
A_51_P338443	NM_020581	Angptl4	0.36	-1.3	0.46	-1.4	0.01	-1.8
A_52_P351925	NM_146005	Ank3	0.02	-1.5	0.19	-1.3	0	-1.8
A_52_P650387	NM_001045530	Ccnjl	0	-1.9	0	-2.1	0	-1.8
A_55_P2050226	AY072938	Ccrl1	0.45	-1.2	0.39	-1.3	0	-1.8
A_55_P1969131	NM_178373	Cidec	0.92	-1.1	0.22	-1.5	0	-1.8
A_55_P2128853	NM_001012766	Ear12	0.38	-1.5	0.02	-2.6	0.05	-1.8
A_55_P2089710	NM_007904	Ednrb	0.75	-1.1	0.18	-1.3	0	-1.8
A_55_P2399718	AK086046	ENSMUSG00000065996	0	-1.5	0.07	-1.3	0	-1.8
A_52_P493620	NM_026218	Fgfr1op2	0.27	-1.3	0.2	-1.4	0	-1.8
A_52_P508991	NM_010231	Fmo1	0.04	-1.4	0.07	-1.4	0	-1.8
A_51_P462428	AK019470	Galntl2	0.61	-1.2	0.45	-1.4	0.01	-1.8
A_55_P2102738	XM_001478003	Gm3781	0.21	-1.5	0.94	-1.1	0.02	-1.8
A_55_P1996952	XM_001479342	Gm4072	0.02	-1.6	0.25	-1.4	0	-1.8
A_55_P2063505	XM_001479508	Gm4235	0	-1.5	0.57	-1.2	0	-1.8
A_55_P2144364	XM_001479624	Gm4320	0.05	-1.5	0.55	-1.3	0	-1.8
A_55_P2120141	XM_001480410	Gm4522	0	-1.5	0.64	-1.2	0	-1.8
A_55_P2135064	NM_008204	H2-M2	0.49	-1.2	0.02	-1.5	0	-1.8

A_55_P2027737	NM_001025602	Il1rl1	0.05	-1.4	0.21	-1.3	0	-1.8
A_55_P2002933	NM_008456	Klk1b5	0.02	-1.8	0	-2.1	0	-1.8
A_55_P2004527	NM_010650	Klra8	0.09	-1.6	0.3	-1.5	0.01	-1.8
A_66_P118093	XM_001480420	LOC100048617	0.21	-1.3	0.02	-1.5	0	-1.8
A_55_P2129469	XM_918544	LOC641199	0.02	-1.5	0	-1.8	0	-1.8
A_55_P1993858	NR_001460	Rmrp	0.13	-1.5	0.48	-1.3	0.01	-1.8
A_55_P2030160	NM_001142920	Tcf7l2	0	-1.5	0	-1.5	0	-1.8
A_52_P45738	NM_029979	Trim35	0	-1.9	0.12	-1.6	0	-1.8
A_55_P2123716	NR_002321	Tug1	0	-1.7	0.12	-1.4	0	-1.8
A_55_P1956762	AK082974		0	-1.6	0.19	-1.3	0	-1.9
A_30_P01031585			0	-1.6	0	-1.6	0	-1.9
A_55_P2167803	NM_001014423	Abi3bp	0	-1.5	0	-1.5	0	-1.9
A_55_P2087984	NM_001164671	Dnaja1	0.14	-1.6	0.04	-1.9	0.01	-1.9
A_51_P153486	NM_018808	Dnajb1	0.08	-1.7	0.09	-1.8	0.01	-1.9
A_52_P577662	NM_007904	Ednrb	0.7	-1.1	0.14	-1.4	0	-1.9
A_55_P2135967	XM_979793	Gm7816	0.19	-1.5	0.03	-1.8	0	-1.9
A_51_P128463	NM_001099296	Grrp1	0.01	-1.5	0	-1.7	0	-1.9
A_51_P155323	NM_010406	Hc	0.44	-1.4	0.48	-1.5	0.03	-1.9
A_52_P163849	NM_001039511	Ivns1abp	0.01	-1.4	0.03	-1.4	0	-1.9
A_55_P2004526	NM_010650	Klra8	0.01	-1.5	0.02	-1.6	0	-1.9
A_55_P2000409	NM_001002786	Rab44	0.31	-1.4	0.99	-1	0.01	-1.9
A_55_P2413458	NM_023755	Tcfcp2l1	0	-1.6	0.15	-1.3	0	-1.9
A_55_P2063465	NM_013691	Thbs3	0.26	-1.2	0	-1.4	0	-1.9
A_55_P2055985	NM_130904	Cd209d	0.36	-1.3	0.04	-1.6	0	-2
A_51_P167292	NM_009892	Chi3l3	0.35	-1.4	0	-2.9	0.01	-2
A_55_P2131766	NM_023665	D4Wsu53e	0.05	-2.4	0.02	-2.9	0.06	-2
A_52_P412585	NM_007894	Ear1	0.33	-1.5	0.01	-2.8	0.02	-2
A_51_P455338	NM_053113	Ear11	0.2	-1.6	0.02	-2.3	0.01	-2
A_51_P487818	NM_017399	Fabp1	0.49	1.2	0.07	-1.5	0	-2
A_55_P2049771	XM_001480935	Gm4621	0.37	-1.3	0.36	-1.4	0	-2
A_51_P356055	NM_175012	Grp	0.16	-1.3	0	-2.1	0	-2
A_55_P1963533	NM_001003672	Pcdhac2	0.35	-1.2	0.6	-1.2	0	-2
A_52_P140881	NM_177615	Slc26a10	0	-1.9	0	-1.9	0	-2
A_55_P2136657	NM_013691	Thbs3	0.14	-1.2	0	-1.5	0	-2
A_55_P1978636			0.61	-1.3	0.99	-1	0.01	-2.1
A_55_P2175469	NM_001081345	Chd2	0.02	-1.5	0.26	-1.3	0	-2.1
A_55_P2157008	NM_053112	Ear10	0.16	-1.6	0.01	-2.8	0.01	-2.1
A_55_P2151638	NM_013793	Klra15	0.09	-1.5	0.06	-1.6	0	-2.1
A_55_P2031989	NM_028724	Rin2	0.04	-1.8	0.14	-1.7	0	-2.1
A_55_P1985950	NM_133213	Xpnpep2	0.98	-1	0.84	-1.2	0	-2.1
A_55_P2465382	AK172117		0.65	-1.3	0.97	-1.1	0.01	-2.2
A_66_P124164	AK089567		0.54	-1.3	0.9	-1.2	0.01	-2.2
A_55_P2138100			0.53	-1.3	0.94	-1.1	0	-2.2
A_55_P2093862	AK156257		0.49	-1.3	0.95	-1.1	0	-2.2
A_55_P2003513	NM_013559	Hsph1	0.95	-1.1	0.33	-1.7	0.01	-2.2
A_55_P2141479	NR_004414	Rnu2	0	-2.1	0.19	-1.6	0	-2.2
A_52_P381484	NM_133903	Spon2	0.07	-1.7	0	-2.8	0	-2.2
A_52_P229052	NM_019790	Tmeff2	0.05	-1.6	0.01	-1.8	0	-2.2
A_55_P2138104	AK089567		0.43	-1.4	0.9	-1.2	0.01	-2.3
A_55_P2003824	NM_009714	Asgr1	0.56	-1.2	0.09	-1.7	0	-2.3
A_51_P287100	NM_007663	Cdh16	0.91	-1.1	0.39	-1.5	0	-2.3
A_51_P124345	NM_153166	Cpne5	0.02	-1.5	0.13	-1.5	0	-2.3
A_55_P2216976	AK143258	D13Ertd608e	0	-2.2	0	-2.4	0	-2.3
A_51_P312485	NM_013521	Fpr1	0.04	-1.6	0	-2.5	0	-2.3
A_51_P179258	NM_001161665	Kif26b	0	-1.7	0.09	-1.5	0	-2.3

A_55_P2000973	NM_181529	Syt15	0.01	-1.4	0	-1.8	0	-2.3
A_55_P2165554			0.53	-1.3	0.94	-1.1	0	-2.4
A_55_P2408588	NM_007489	Arntl	0.08	-1.6	0	-2.9	0	-2.4
A_52_P514407	NM_013793	Klra15	0.02	-1.7	0.03	-1.8	0	-2.4
A_55_P2165790	NM_178706	Siglech	0	-1.9	0	-2.4	0	-2.5
A_30_P01024606			0	-1.7	0.06	-1.5	0	-2.6
A_30_P01032068			0	-1.9	0	-1.9	0	-2.6
A_55_P2068459	NM_010479	Hspa1a	0.19	-1.9	0.07	-2.7	0.01	-2.6
A_52_P257625	NM_023612	Esm1	0.06	-1.6	0	-2.1	0	-2.7
A_55_P1973930	NM_146063	Krt79	0.38	-1.2	0.1	-1.4	0	-2.8
A_52_P638459	NM_013653	Ccl5	0	-2.1	0.01	-2.4	0	-3.3
A_55_P2004536	NM_010649	Klra4	0	-2.4	0	-2.5	0	-3.5
A_55_P2028734	NM_013794	Klra16	0	-2.4	0.01	-2.5	0	-3.7
A_55_P2170349	NM_053152	Klra22	0	-2.2	0.01	-2.2	0	-3.9
A_51_P175424	NM_011797	Car14	0	-2.7	0	-3.6	0	-4.1
A_51_P331328	NM_026730	Gpihbp1	0.08	-1.6	0	-2.3	0	-5.4
A_55_P2094060	NM_010370	Gzma	0	-3	0	-5.1	0	-9.5
A_55_P2011341	XM_001474162	LOC100045268	0	-3.1	0	-5.1	0	-9.5

2.3 CT-Mix. Significant probe list. List of all significantly differentially expressed probes in at least one treatment group (FDR P ≤ 0.05, fold change ± 1.5) in response to sub-chronic oral exposure to 1.25, 2.5, and 5.1 mg BaP equivalents /kg-bw/day of a mixture of 8 PAHs in the lungs. The list is sorted from highest to lowest fold change in the 5.1 mg BaP equivalents/kg-bw/day treatment group.

			Low dose 1.25 mg BaP equivalents/ kg-day		Medium dose 2.5 mg BaP equivalents/ kg-day		High dose 5.1 mg BaP equivalents/ kg-day	
Agilent Probe	Accession Number	Gene Symbol	*FDR P value*	*Fold change*	*FDR P value*	*Fold change*	*FDR P value*	*Fold change*
A_51_P279693	NM_009992	Cyp1a1	0.75	3	0	7.1	0	9.2
A_55_P1960735	NM_011819	Gdf15	0	4.9	0	6.5	0	9.1
A_51_P255456	NM_009994	Cyp1b1	0.53	1.9	0	3.7	0	7.9
A_51_P363947	NM_007669	Cdkn1a	0	2.2	0	2.9	0	4.9
A_55_P1959500	NM_172759	Ces5	0	2.7	0	3.7	0	4.7
A_55_P2032081	NM_016974	Dbp	0	4.4	0	5.5	0	4.5
A_55_P1986282	NM_001111099	Cdkn1a	0	2.3	0	3.2	0	4.3
A_55_P2032079	NM_016974	Dbp	0	4.1	0	5.6	0	4.2
A_51_P254425	NM_009644	Ahrr	0.19	1.9	0	3.3	0	3.7
A_51_P329928	NM_013750	Phlda3	0	2	0	2.5	0	3.3
A_55_P1972948	NM_176954	Brunol5	0.92	1.3	0	2	0	2.9
A_66_P118600	NM_008480	Lama1	0.13	2.2	0	2.5	0	2.8
A_55_P2137406	NM_007527	Bax	0.37	1.9	0	2.6	0	2.7
A_51_P414396	NM_153127	Mmrn2	0.15	2.1	0.06	2.1	0	2.7
A_51_P247184	NM_008728	Npr3	0.18	2.1	0.03	2.3	0	2.6
A_51_P453736	NM_001143686	Apol11b	0.9	1.9	0.82	1.6	0.03	2.6
A_55_P1965154	NM_025565	Spc25	0	2.1	0	2.6	0	2.5
A_52_P612803	NM_009831	Ccng1	0.07	1.5	0	2	0	2.5
A_52_P29953	NM_175398	6530418L21Rik	0.15	1.8	0	2	0	2.4
A_51_P231320	NM_008611	Mmp8	0.98	1.5	0.24	1.8	0	2.4
A_55_P2145804	NM_026531	Aen	0	1.7	0	2	0	2.3

A_55_P2141860	NM_026531	Aen	0	1.7	0	1.9	0	2.3
A_66_P115580	AK076360		0	1.6	0	1.9	0	2.3
A_52_P539310	NM_001160326	Serp2	0.48	1.4	0	1.9	0	2.3
A_55_P2005213	NM_145603	Ces2	0.75	1.5	0	1.9	0	2.3
A_52_P162099	NM_001004140	Ckap2	0.87	1.5	0.1	1.9	0	2.3
A_51_P253803	NM_001081117	Mki67	0.49	1.8	0.04	2.1	0	2.2
A_55_P2002577	NM_010145	Ephx1	0.15	1.6	0	1.9	0	2.2
A_30_P01027010			0.19	1.5	0	1.8	0	2.2
A_51_P307168	NM_026993	Ddah1	0.67	1.5	0.19	1.6	0	2.2
A_55_P2101340	NM_019511	Ramp3	0.13	1.7	0.06	1.6	0	2.2
A_51_P451151	NM_026785	Ube2c	0.83	1.5	0.32	1.6	0	2.2
A_55_P2002578	NM_010145	Ephx1	0	1.7	0	2	0	2.1
A_51_P481920	NM_009828	Ccna2	0.5	1.8	0.03	2	0.01	2.1
A_51_P449824	XM_001471750	Exoc3l2	1	1.4	0.02	1.9	0	2.1
A_55_P1954835	XM_001475752	LOC100046186	0.07	1.9	0.02	1.8	0	2.1
A_51_P323620	NM_144543	Thyn1	0	1.4	0	1.7	0	2.1
A_52_P588881	NM_001033484	Iqgap3	1	1.5	0.38	1.7	0.02	2.1
A_30_P01026923			0.87	1.5	0.17	1.8	0	2.1
A_51_P329332	NM_054087	Slc19a2	0.67	1.4	0.06	1.6	0	2.1
A_51_P185906	NM_025659	Abi3	1	1.2	0.03	2.3	0.01	2
A_51_P455897	NM_144526	Fam64a	0.22	1.8	0.02	2	0.01	2
A_55_P2173982	NM_009104	Rrm2	0.3	1.5	0	1.9	0	2
A_55_P1983773	NM_001012273	Birc5	0.38	1.6	0.03	1.8	0	2
A_55_P1967291	NM_144818	Ncaph	0.13	1.6	0.02	1.8	0	2
A_55_P2031999	NM_145448	9030617O03Rik	0.95	1.3	0.02	1.7	0	2
A_51_P353392	NM_019936	Cript	0.11	1.6	0.05	1.6	0	2
A_55_P2119257	NM_008871	Serpine1	0.25	1.4	0	1.6	0	2
A_55_P1988228	NM_009791	Aspm	1	1.5	0.41	1.7	0.03	2
A_55_P2007713	NM_053082	Tspan4	0.43	2	0.1	2.1	0.03	2
A_55_P2056729	NM_008342	Igfbp2	1	1.3	0.37	1.7	0.03	2
A_51_P149714	NM_026835	Ms4a6d	1	1.4	0.47	1.5	0.02	2
A_51_P223776	NM_145434	Nr1d1	0.22	1.8	0.08	1.9	0.01	2
A_30_P01026536			0.8	1.4	0.19	1.6	0	2
A_55_P2094925	NM_011157	Srgn	0.77	1.3	0.13	1.5	0	2
A_55_P2035286	NM_010931	Uhrf1	0.19	1.5	0	2	0	1.9
A_51_P240453	NM_133851	Nusap1	0.6	1.5	0.02	1.9	0.01	1.9
A_51_P175580	NM_021897	Trp53inp1	0.11	1.5	0	1.8	0	1.9
A_30_P01020960			0.5	1.5	0	1.7	0	1.9
A_51_P383032	NM_010819	Clec4d	0.88	1.3	0.04	1.6	0	1.9
A_51_P415220	NM_009517	Zmat3	0.36	1.3	0	1.6	0	1.9
A_52_P232637	NM_007857	Dhh	0.53	1.7	0.54	1.5	0.04	1.9
A_55_P2129316			1	1.4	0.91	1.2	0.03	1.9
A_55_P2073377	NM_001081117	Mki67	0.72	1.6	0.23	1.7	0.02	1.9
A_30_P01019901			1	1.3	0.37	1.6	0.02	1.9
A_51_P515965	NM_008685	Nfe2	0.58	1.7	0.2	1.7	0.02	1.9
A_51_P133137	NM_009004	Kif20a	0.8	1.5	0.35	1.6	0.01	1.9
A_55_P2143025	NM_013657	Sema3c	0.87	1.4	0.37	1.4	0.01	1.9
A_66_P135391	NM_008342	Igfbp2	1	1.3	0.24	1.7	0.01	1.9
A_51_P230098	NM_023209	Pbk	0.96	1.5	0.22	1.8	0.01	1.9
A_55_P2063146			0.75	1.5	0.28	1.5	0	1.9
A_55_P1960238	NM_172659	Slc2a6	1	1.1	0.48	1.4	0	1.9
A_52_P151393	NM_198860	AI646023	1	1.2	0.4	1.5	0	1.9
A_52_P700056	NM_001081957	Gm11428	1	1.3	0.21	1.5	0	1.9
A_55_P2062543	NM_053173	Kifc1	0.89	1.4	0.21	1.6	0	1.9
A_51_P487999	NM_028232	Sgol1	0.25	1.5	0.03	1.7	0	1.8

A_51_P156438	NM_027460	Slc25a33	0.5	1.4	0.02	1.7	0	1.8
A_51_P148105	NM_011234	Rad51	0.85	1.3	0.03	1.6	0	1.8
A_55_P2168628	NM_133678	Sac3d1	0.19	1.4	0	1.5	0	1.8
A_51_P350403	NM_009148	Exoc4	0.15	1.4	0	1.5	0	1.8
A_30_P01025790			0.19	1.3	0	1.5	0	1.8
A_52_P686785	NM_053247	Lyve1	0.53	1.7	0.09	1.8	0.03	1.8
A_55_P2064771	NM_010741	Ly6c1	0.93	1.4	0.78	1.3	0.02	1.8
A_55_P1996946	NM_023223	Cdc20	1	1.3	0.3	1.5	0.02	1.8
A_51_P204442	NM_028716	Phf19	0.96	1.4	0.11	1.6	0.01	1.8
A_51_P405397	NM_007899	Ecm1	1	1.3	0.33	1.4	0	1.8
A_52_P281145	NM_172872	Kank4	0.81	1.3	0.41	1.3	0	1.8
A_55_P2152035	NM_001163763	Tcf19	1	1.3	0.22	1.6	0	1.8
A_55_P2062598	NM_178609	E2f7	0.49	1.5	0.11	1.6	0	1.8
A_55_P2110245	NM_001163359	Fignl1	0.92	1.3	0.1	1.6	0	1.8
A_55_P1979893	NM_017376	Tef	0.19	2	0.02	2.3	0.1	1.7
A_52_P303891	NM_011584	Nr1d2	0.32	1.9	0	2.2	0.07	1.7
A_30_P01030240			1	1.3	0.59	1.4	0.04	1.7
A_51_P507801	NM_028784	F13a1	1	1.3	0.16	1.7	0.04	1.7
A_51_P115005	NM_010104	Edn1	0.99	1.4	0.11	1.7	0.03	1.7
A_55_P2026223	NM_011373	St6galnac4	1	1.2	0.96	1.2	0.03	1.7
A_51_P513530	NM_017407	Spag5	1	1.3	0.77	1.3	0.02	1.7
A_30_P01031674			1	1.2	0.68	1.4	0.02	1.7
A_55_P2158011	NM_026412	D2Ertd750e	1	1.3	0.42	1.4	0.02	1.7
A_30_P01023554			1	1.3	0.49	1.4	0.02	1.7
A_55_P2143572	AK020725		1	1.3	0.7	1.3	0.02	1.7
A_55_P2011937	NM_001163359	Fignl1	1	1.2	0.26	1.5	0.01	1.7
A_51_P164014	NM_173762	Cenpe	0.65	1.4	0.31	1.5	0.01	1.7
A_55_P2127702	NM_012025	Racgap1	0.98	1.3	0.16	1.5	0.01	1.7
A_66_P111562	NM_007631	Ccnd1	1	1.2	0.6	1.3	0.01	1.7
A_55_P2164469	NM_001163318	Gm11744	0.19	1.7	0.09	1.6	0.01	1.7
A_55_P1976127	NM_007900	Ect2	1	1.3	0.18	1.5	0	1.7
A_52_P675395	NM_007722	Cxcr7	1	1.2	0.45	1.4	0	1.7
A_55_P2042016	XM_001479435	LOC100048058	0.6	1.3	0.4	1.3	0	1.7
A_52_P69558	XM_001475933	Gm8221	0.87	1.3	0.16	1.5	0	1.7
A_30_P01028287			0.74	1.4	0.37	1.4	0	1.7
A_51_P270184	NM_025811	Nhlrc2	0.87	1.4	0.1	1.6	0	1.7
A_51_P179697	NM_026884	Fam57b	0.82	1.3	0.19	1.4	0	1.7
A_51_P185660	NM_011338	Ccl9	1	1.2	0.34	1.5	0	1.7
A_51_P204402	NM_011369	Shcbp1	0.6	1.4	0.07	1.5	0	1.7
A_51_P330213	NM_024184	Asf1b	0.94	1.3	0.17	1.5	0	1.7
A_55_P2040276	NM_001163318	Gm11744	0.62	1.4	0.11	1.5	0	1.7
A_51_P125135	NM_026410	Cdca5	0.58	1.4	0.06	1.6	0	1.7
A_51_P282760	NM_011066	Per2	0.38	1.8	0.02	2.1	0.09	1.6
A_55_P2168168	NM_031384	Tex11	0.39	1.5	0.02	1.8	0.01	1.6
A_55_P2052062	NM_010818	Cd200	0.15	1.6	0	1.8	0.01	1.6
A_51_P161946	NM_175332	E130012A19Rik	0.13	1.5	0	1.8	0.01	1.6
A_55_P2095271	NM_153805	Pkn3	0.69	1.4	0.05	1.5	0	1.6
A_55_P2039320	NM_178679	Zfp365	0.93	1.2	0	1.5	0	1.6
A_30_P01029956			0.39	1.2	0	1.5	0	1.6
A_51_P472217	NM_001081085	2010317E24Rik	0.63	1.5	0.56	1.4	0.04	1.6
A_55_P2038106	NM_009978	Cst8	0.36	1.6	0.14	1.6	0.03	1.6
A_66_P104815	NM_007899	Ecm1	1	1.3	0.6	1.4	0.03	1.6
A_51_P369200	NM_028109	Tpx2	0.98	1.3	0.24	1.5	0.03	1.6
A_30_P01019152			1	1.1	0.71	1.3	0.03	1.6
A_55_P2000833	NM_001013368	E2f8	0.96	1.3	0.42	1.4	0.03	1.6

A_51_P457528	NM_007630	Ccnb2	1	1.3	0.41	1.5	0.03	1.6
A_55_P2011146	NM_178608	Reep1	0.58	1.4	0.18	1.5	0.02	1.6
A_55_P1999561	NM_001002842	Pram1	1	1.1	0.33	1.4	0.02	1.6
A_51_P433615	NM_183390	Klhl6	1	1.2	0.45	1.4	0.02	1.6
A_51_P331752	NM_011330	Ccl11	1	1.2	0.82	1.2	0.02	1.6
A_55_P2103706	XM_485921	Gm5593	1	1.3	0.37	1.4	0.02	1.6
A_52_P40504	AK162948		1	1.2	0.32	1.4	0.02	1.6
A_51_P239984	NM_012012	Exo1	1	1.2	0.37	1.4	0.02	1.6
A_30_P01023701			0.53	1.5	0.51	1.4	0.02	1.6
A_51_P290576	NM_152804	Plk2	1	1.1	0.61	1.3	0.02	1.6
A_51_P158210	NM_008564	Mcm2	1	1.2	0.33	1.4	0.01	1.6
A_55_P2085295	NM_198411	Inf2	0.51	1.3	0.06	1.4	0.01	1.6
A_51_P367310	NM_028083	Chaf1b	0.98	1.3	0.49	1.3	0.01	1.6
A_51_P516133	NM_015786	Hist1h1c	1	1.2	0.53	1.2	0.01	1.6
A_52_P30989	NM_028222	Cdkn3	1	1.2	0.51	1.3	0.01	1.6
A_55_P2000533	NM_012048	Polk	1	1.1	0.49	1.3	0.01	1.6
A_55_P2072631	NM_001083810	Prr5l	0.87	1.3	0.13	1.5	0.01	1.6
A_55_P2106150	NM_021790	Cenpk	1	1.2	0.22	1.4	0.01	1.6
A_55_P2090359	NM_148937	Plcd4	0.81	1.3	0.34	1.4	0	1.6
A_52_P89567	NM_007483	Rhob	1	1.2	0.16	1.4	0	1.6
A_52_P220810	NM_144551	Trib2	0.6	1.2	0	1.4	0	1.6
A_55_P2173313	NM_019677	Plcb1	0.53	1.4	0.75	1.3	0	1.6
A_55_P2083023	NM_133678	Sac3d1	0.15	1.3	0	1.4	0	1.6
A_55_P2021114	NM_010500	Ier5	0.07	1.5	0.02	1.4	0	1.6
A_55_P2027836	NM_020275	Tnfrsf10b	0.23	1.4	0.31	1.3	0	1.6
A_52_P527800	NM_145158	Emilin2	1	1.2	0.17	1.4	0	1.6
A_55_P1976204	U09507		0.98	1.2	0.15	1.4	0	1.6
A_51_P413785	NM_147778	Commd3	0.67	1.2	0	1.4	0	1.6
A_55_P2045258	NM_010119	Ehd1	1	1.2	0.65	1.3	0	1.6
A_52_P167278	NM_172308	Mthfd1l	0.8	1.4	0.04	1.7	0.05	1.5
A_55_P2083919	NM_175549	Robo2	0.51	1.4	0.04	1.6	0.03	1.5
A_51_P155458	AK170454		0.11	1.5	0	1.6	0	1.5
A_55_P1986833	NM_054040	Tulp4	0.25	1.4	0	1.6	0	1.5
A_66_P136186	NM_009516	Wee1	1	1.2	0.04	1.5	0.03	1.5
A_55_P2011620	NM_181754	Gpr141	1	1.2	0.67	1.3	0.04	1.5
A_51_P401907	NM_001082547	Gm5483	1	1.1	0.73	1.2	0.04	1.5
A_52_P679105	NM_029614	Prss23	1	1.2	0.4	1.4	0.04	1.5
A_30_P01019819			1	1.2	0.77	1.3	0.04	1.5
A_52_P431859	AK167772		1	1.2	0.9	1.2	0.04	1.5
A_52_P114905	BC049666		0.93	1.3	0.79	1.2	0.04	1.5
A_51_P203675	NM_021407	Trem3	1	1.2	0.66	1.3	0.04	1.5
A_51_P498631	NM_018769	Dfna5	1	1.1	0.82	1.2	0.03	1.5
A_52_P607128	NM_031195	Msr1	1	1	0.85	1.2	0.03	1.5
A_51_P354706	NM_010094	Lefty1	0.94	1.3	0.42	1.3	0.03	1.5
A_51_P181286	NM_001033122	Cd69	1	-1	0.79	1.2	0.03	1.5
A_51_P431996	NM_175494	Zfp367	1	1.1	0.53	1.3	0.02	1.5
A_55_P2013336	NM_010790	Melk	1	1.2	0.55	1.3	0.02	1.5
A_55_P2077263	NM_021790	Cenpk	0.94	1.2	0.19	1.4	0.02	1.5
A_51_P264527	NM_019833	Fam69b	0.96	1.2	0.09	1.4	0.02	1.5
A_52_P311853	NM_030143	Ddit4l	1	1.1	0.85	1.2	0.02	1.5
A_55_P2405784	AK141429	BC023202	1	1.2	0.77	1.2	0.02	1.5
A_51_P418116	NM_146162	Tmem119	1	1.2	0.57	1.3	0.02	1.5
A_52_P536494	NM_008709	Mycn	1	1.2	0.47	1.3	0.02	1.5
A_55_P1996941	NM_026785	Ube2c	0.94	1.3	0.45	1.3	0.02	1.5
A_55_P2078365	NM_173402	Rgs12	0.74	1.3	0.1	1.4	0.01	1.5

A_55_P2004801	NM_001040435	Tacc3	0.94	1.2	0.14	1.4	0.01	1.5
A_55_P2048119	NM_146257	Slc29a4	0.98	1.2	0.56	1.3	0.01	1.5
A_30_P01023785			0.32	1.4	0.31	1.4	0.01	1.5
A_30_P01024344			1	1.2	0.05	1.4	0.01	1.5
A_30_P01032002			1	1.1	0.17	1.3	0.01	1.5
A_55_P2177154	NM_178200	Hist1h2bm	1	1.1	0.88	1.2	0.01	1.5
A_51_P195875	NM_010929	Notch4	0.33	1.3	0.16	1.3	0.01	1.5
A_51_P133612	NM_026014	Cdt1	1	1.2	0.09	1.5	0.01	1.5
A_55_P1953728	NM_016701	Nes	0.65	1.2	0.07	1.3	0	1.5
A_30_P01025511			1	1.2	0.2	1.4	0	1.5
A_55_P2169227	NM_177716	AI836003	1	1.2	0.09	1.4	0	1.5
A_51_P337089	NM_027014	Gins1	1	1	0.15	1.3	0	1.5
A_55_P1965150	NM_021549	Pnkp	1	1.2	0.6	1.2	0	1.5
A_51_P246903	NM_026467	Rps27l	0.43	1.2	0	1.3	0	1.5
A_55_P2125208	NM_145996	Arid5a	0.88	1.3	0.82	1.2	0	1.5
A_51_P126626	NM_145459	Zfp503	0.5	1.2	0	1.4	0	1.5
A_66_P119034	NM_013737	Pla2g7	1	1.2	0.2	1.4	0	1.5
A_30_P01025143			0.36	1.3	0.02	1.4	0	1.5
A_52_P629748	AK028718		0.76	1.2	0.89	1.1	0	1.5
A_55_P1981949	NM_175174	Klhl5	0.44	1.2	0	1.4	0	1.5
A_55_P2027022	NM_007548	Prdm1	1	1.2	0.07	1.4	0	1.5
A_51_P156434	NM_027460	Slc25a33	1	1.2	0.12	1.4	0	1.5
A_52_P393314	NM_011027	P2rx7	0.93	1.2	0.11	1.4	0	1.5
A_55_P2043554	NM_009195	Slc12a4	0.51	1.3	0.09	1.3	0	1.5
A_51_P224564	NM_176833	Ppm1f	0.53	1.2	0.07	1.3	0	1.5
A_52_P139650	NM_025581	Ska1	0.64	1.3	0.02	1.6	0.04	1.4
A_52_P348250	NM_001164493	Klhl29	0.81	1.3	0	1.6	0.06	1.4
A_51_P477121	NM_021451	Pmaip1	0	1.4	0	1.5	0.02	1.4
A_55_P1973906	NM_021897	Trp53inp1	0.65	1.3	0	1.5	0.02	1.4
A_51_P258493	NM_011067	Per3	0.36	1.7	0.04	1.9	0.37	1.3
A_55_P2039225	NM_001081155	Rap1gap	0.53	1.4	0.04	1.6	0.14	1.3
A_52_P293120	NM_001081135	Prrg3	0.13	1.4	0.02	1.5	0.07	1.3
A_52_P354123	NM_026967	Rhebl1	0.19	1.4	0	1.5	0.07	1.3
A_30_P01025072			0	1.5	0.09	1.3	0.49	1.1
A_55_P1979674	NM_011772	Ikzf4	0.93	-1.3	0.03	-1.5	0.32	-1.2
A_55_P2179604	NM_177354	Vash1	1	1	0.99	-1	0.04	-1.5
A_51_P347452	NM_028242	Htatsf1	1	-1.2	0.95	-1.1	0.04	-1.5
A_55_P2087182	NM_007607	Car4	1	-1.1	0.97	-1.1	0.04	-1.5
A_55_P1989813	NM_008103	Gcm1	1	1	1	-1	0.04	-1.5
A_55_P2146483	NM_027230	Zmynd8	1	-1.1	0.98	-1.1	0.04	-1.5
A_51_P485458	NM_001005506	Txlna	1	-1.1	0.94	-1.1	0.04	-1.5
A_55_P2067960	NM_009946	Cplx2	1	-1	0.99	-1	0.04	-1.5
A_52_P256279	NM_173026	Zbtb11	1	-1.1	0.97	-1.1	0.04	-1.5
A_30_P01018341			1	-1.2	0.39	-1.3	0.04	-1.5
A_55_P2133646	NM_199316	4922501C03Rik	1	-1.2	0.63	-1.3	0.04	-1.5
A_55_P2173373	NM_001024846	Zfp62	1	-1.1	0.81	-1.2	0.03	-1.5
A_30_P01031360			1	-1	0.95	-1.1	0.03	-1.5
A_30_P01032449			1	-1.1	0.66	-1.3	0.03	-1.5
A_55_P1959639	XM_001473594	LOC100045061	1	-1	0.99	-1.1	0.03	-1.5
A_55_P2082478	NM_001081378	Kidins220	1	-1.1	0.89	-1.2	0.03	-1.5
A_55_P2126627	NM_001081409	Phf20l1	1	-1.1	0.82	-1.2	0.03	-1.5
A_30_P01029077			1	-1.1	0.9	-1.1	0.03	-1.5
A_55_P2002449	XM_001473799	4932422M17Rik	1	-1.2	0.5	-1.3	0.03	-1.5
A_55_P2102454	XM_356668	Gm5196	1	-1.1	0.91	-1.2	0.03	-1.5
A_55_P2020004	XM_001476005	Gm3218	1	-1	0.99	-1.1	0.03	-1.5

A_30_P01019455			1	-1.2	0.97	-1.1	0.03	-1.5
A_30_P01021097			1	-1.1	0.75	-1.3	0.03	-1.5
A_51_P229599	NM_029250	Etnk1	1	-1.1	0.8	-1.2	0.03	-1.5
A_55_P2040774	XM_900336	Gm6837	1	-1	0.99	-1	0.03	-1.5
A_30_P01025590			1	-1.1	0.95	-1.1	0.03	-1.5
A_30_P01018146			1	-1.2	0.88	-1.2	0.03	-1.5
A_55_P2096121	XM_001473876	Gm2528	1	-1	0.99	-1	0.03	-1.5
A_30_P01030728			1	-1.1	0.65	-1.3	0.02	-1.5
A_30_P01017999			1	-1.1	0.96	-1.1	0.02	-1.5
A_55_P2399718	AK086046	ENSMUSG00000065996	1	-1.1	0.84	-1.2	0.02	-1.5
A_30_P01031683			1	-1.1	0.7	-1.2	0.02	-1.5
A_30_P01031680			1	-1.2	0.85	-1.2	0.02	-1.5
A_55_P2378827	AK047890	LOC553096	1	1	0.79	-1.2	0.02	-1.5
A_52_P501049	BC002059		1	-1.1	0.83	-1.2	0.02	-1.5
A_55_P2005720	XM_001479578	Gm4253	1	1	0.99	-1	0.02	-1.5
A_55_P2131520	NM_177354	Vash1	1	-1	0.99	-1.1	0.02	-1.5
A_55_P2143119	XM_001476489	LOC100046136	0.96	-1.3	0.53	-1.3	0.02	-1.5
A_55_P1987245	NM_011740	Ywhaz	1	-1	0.95	-1.1	0.02	-1.5
A_55_P2167447	NM_001039551	Cnnm3	1	-1.1	0.97	-1.1	0.02	-1.5
A_52_P291971	NM_025303	Stau2	1	-1.1	0.18	-1.5	0.02	-1.5
A_55_P2129867	NM_008018	Sh3pxd2a	1	-1	0.75	-1.2	0.02	-1.5
A_55_P2375121	AK045926	AI225934	1	1	1	1	0.02	-1.5
A_55_P2020472	NM_027425	Rufy2	1	-1.1	0.86	-1.2	0.02	-1.5
A_65_P01643	NM_001081345	Chd2	1	-1.1	0.98	-1.1	0.02	-1.5
A_55_P2161923	NM_001033960	Rabgap1	1	-1.1	0.95	-1.1	0.01	-1.5
A_30_P01025538			1	-1.1	0.88	-1.2	0.01	-1.5
A_55_P2040878	NM_001040691	Ung	1	-1.2	0.66	-1.2	0.01	-1.5
A_30_P01020373			1	-1	0.94	-1.1	0.01	-1.5
A_55_P1970537	NM_009745	Bcl7b	1	-1.1	0.6	-1.3	0.01	-1.5
A_55_P2118609	NM_011371	St6galnac1	1	-1	0.97	-1.1	0.01	-1.5
A_66_P127262	XM_985872	Gm8799	1	-1.1	0.9	-1.2	0.01	-1.5
A_52_P18807	NM_146200	Eif3c	1	-1.2	0.76	-1.2	0.01	-1.5
A_30_P01027369			0.4	-1.3	0.15	-1.3	0.01	-1.5
A_55_P2259125	NR_028425	LOC100303645	1	-1	0.82	-1.2	0.01	-1.5
A_55_P1975660	NM_053149	Hemgn	1	-1.2	0.66	-1.2	0.01	-1.5
A_55_P1967133	NM_177741	Ppp1r3b	1	-1.2	0.78	-1.2	0.01	-1.5
A_55_P2132472	NM_013830	Prpf4b	1	-1.1	0.49	-1.3	0.01	-1.5
A_55_P2131820	AK079921		0.68	-1.2	0.27	-1.2	0.01	-1.5
A_55_P1978441	NM_008443	Kif3a	1	-1.1	0.69	-1.2	0.01	-1.5
A_55_P2015403	NM_001114679	RP23-269N23.3	0.94	-1.2	0.73	-1.2	0.01	-1.5
A_66_P106133	NM_133853	Magi3	1	1.1	0.93	-1.1	0.01	-1.5
A_55_P2112967	NM_007858	Diap1	1	-1.1	0.75	-1.2	0.01	-1.5
A_51_P359272	NM_009655	Alcam	1	-1.1	0.71	-1.2	0.01	-1.5
A_55_P1971867	NM_153143	Kctd11	1	-1	0.82	-1.2	0.01	-1.5
A_51_P445487	NM_026629	2410066E13Rik	1	-1.1	0.93	-1.1	0.01	-1.5
A_30_P01025227			1	-1	0.94	-1.1	0.01	-1.5
A_30_P01026302			1	-1.1	0.81	-1.2	0.01	-1.5
A_51_P379341	NM_010895	Neurod2	1	-1.1	0.85	-1.2	0.01	-1.5
A_55_P2292737	AK086402	2310002F09Rik	1	-1.1	0.75	-1.2	0.01	-1.5
A_55_P2235387	AK079318	9630020I17Rik	1	-1.1	0.49	-1.2	0	-1.5
A_55_P2122633	AK032756	Airn	1	-1.1	0.48	-1.2	0	-1.5
A_55_P2156556	XM_001472797	Gm2212	1	-1.1	0.7	-1.2	0	-1.5
A_30_P01025521			1	-1.1	0.41	-1.2	0	-1.5
A_55_P2082614	NM_001037745	Zfp791	1	-1.1	0.58	-1.2	0	-1.5
A_55_P2075894	XM_356811	Gm5215	1	-1.1	0.89	-1.1	0	-1.5

A_55_P1973399	AK163102		1	-1.1	0.53	-1.2	0	-1.5
A_55_P2144436	NM_010925	Rrp1	1	-1.1	0.34	-1.3	0	-1.5
A_55_P2099890	NM_139227	Atxn7	1	-1.2	0.48	-1.2	0	-1.5
A_55_P2169069	AK138421		1	-1.1	0.52	-1.2	0	-1.5
A_51_P484842	NM_007493	Asgr2	0.5	-1.3	0.39	-1.3	0	-1.5
A_55_P1966583	NM_177772	Bpil2	0.8	-1.2	0.38	-1.2	0	-1.5
A_55_P2173664	XM_001474080	Gm2595	0.95	-1.2	0.47	-1.2	0	-1.5
A_55_P2130905	XM_001471976	Gm1983	1	-1.1	0.95	-1.1	0	-1.5
A_55_P2423586	NM_010164	Eya1	1	-1.2	0.49	-1.2	0	-1.5
A_52_P518434	NM_177338	Hmbox1	1	-1.1	0.1	-1.3	0	-1.5
A_55_P2067952	XM_991957	Gm5097	0.61	-1.3	0.44	-1.2	0	-1.5
A_55_P1997300	NM_027930	Fam54a	1	-1.2	0.64	-1.2	0	-1.5
A_30_P01026271			1	-1	0.97	-1.1	0	-1.5
A_55_P2209308	AK079287	9530083O12Rik	1	-1.2	0.42	-1.2	0	-1.5
A_55_P2067463	XM_001477589	Gm3618	1	-1.2	0.53	-1.2	0	-1.5
A_55_P2090782	AK157058		0.93	-1.2	0.72	-1.2	0	-1.5
A_55_P2062911	NM_007937	Epha5	1	-1.1	0.82	-1.1	0	-1.5
A_55_P2029235	NM_013483	Btn1a1	0.97	-1.2	0.38	-1.2	0	-1.5
A_55_P2054728	NM_178886	Ldlrad3	1	-1.1	0.77	-1.2	0	-1.5
A_55_P1956482	AK165204	Epb4.9	0.95	-1.2	0.19	-1.3	0	-1.5
A_55_P2062793	NM_008546	Mfap2	1	-1.2	0.28	-1.4	0	-1.5
A_30_P01020464			0.73	-1.2	0.19	-1.3	0	-1.5
A_55_P2101666	NM_016872	Vamp5	1	-1	0.91	-1.1	0	-1.5
A_55_P1968863	NM_175132	Synpo2l	1	-1.1	0.72	-1.2	0	-1.5
A_30_P01027949			1	-1.1	0.92	-1.1	0	-1.5
A_55_P2027012	NM_011459	Serpinb8	1	-1.1	0.88	-1.1	0	-1.5
A_30_P01019779			1	-1.1	0.07	-1.3	0	-1.5
A_55_P2035087	NM_177566	Arhgef15	0.58	-1.2	0.03	-1.3	0	-1.5
A_55_P2084251	NM_021275	Kcna4	1	-1.1	0.32	-1.2	0	-1.5
A_55_P2117146	NM_011119	Pa2g4	0.93	-1.2	0.3	-1.3	0	-1.5
A_52_P88054	NM_007699	Chrm4	1	-1	0.33	-1.2	0	-1.5
A_55_P2424767	NM_001081348	Hecw1	1	-1.1	0.67	-1.2	0	-1.5
A_30_P01027463			1	-1.1	0.82	-1.2	0	-1.5
A_55_P2065829	NM_199017	9230110C19Rik	1	-1	0.95	-1.1	0	-1.5
A_51_P368394	NM_019964	Dnajb8	1	-1.1	0.75	-1.1	0	-1.5
A_55_P2005315	AK140012		1	-1.1	0.49	-1.2	0	-1.5
A_30_P01025204			1	-1.1	0.35	-1.2	0	-1.5
A_55_P2017709	NM_033608	Igsf9	0.93	-1.2	0.44	-1.2	0	-1.5
A_55_P2038484	NM_175017	4933427D06Rik	0.76	-1.2	0.42	-1.2	0	-1.5
A_55_P1990874	NM_016812	Banp	1	-1	0.96	-1.1	0	-1.5
A_55_P1987290	NR_004413	Rnu1b6	1	-1.2	0.05	-1.5	0	-1.6
A_55_P2021011	NM_030207	Sfi1	1	-1.3	0.88	-1.2	0.04	-1.6
A_66_P118165	XM_001471574	LOC100038935	1	-1.2	0.81	-1.3	0.04	-1.6
A_55_P1963491	NM_008972	Ptma	1	-1.1	0.97	-1.1	0.04	-1.6
A_55_P2076797	NM_007840	Ddx5	1	-1	0.97	-1.1	0.04	-1.6
A_52_P344376	NM_013506	Eif4a2	1	1	0.91	-1.2	0.04	-1.6
A_55_P2040168	NM_008885	Pmp22	1	-1.1	0.88	-1.2	0.04	-1.6
A_55_P2058601	XM_920293	Gm7097	1	-1.1	0.92	-1.2	0.04	-1.6
A_55_P2139913	NM_009278	Ssb	1	-1.2	0.59	-1.4	0.04	-1.6
A_30_P01028021			1	-1.2	0.93	-1.2	0.04	-1.6
A_55_P1955733	XM_001477303		1	-1.1	0.92	-1.2	0.03	-1.6
A_52_P5549	NM_001042501	Fam133b	1	-1.2	0.83	-1.2	0.03	-1.6
A_55_P2043782	NM_001039104	Trpm1	1	-1.2	0.99	-1.1	0.03	-1.6
A_30_P01018833			1	-1.1	0.72	-1.3	0.03	-1.6
A_51_P265008	NM_001081086	Ppig	1	-1.1	0.42	-1.4	0.02	-1.6

A_30_P01032901			1	-1.1	0.9	-1.2	0.02	-1.6
A_66_P105422	NM_028894	Lonrf3	1	-1.2	0.8	-1.3	0.02	-1.6
A_30_P01023977			1	-1.1	0.88	-1.2	0.02	-1.6
A_55_P2090142	NM_010596	Kcna7	1	-1.1	0.89	-1.2	0.02	-1.6
A_55_P2030771	NM_019406	Fnbp1	1	-1.1	0.86	-1.2	0.02	-1.6
A_52_P590474	NM_019937	Ccnl1	1	-1.1	0.76	-1.3	0.02	-1.6
A_30_P01031405			1	-1.2	0.81	-1.2	0.02	-1.6
A_52_P97670	NM_001099628	Atad2b	1	-1.1	0.93	-1.2	0.02	-1.6
A_30_P01028210			1	-1.2	0.97	-1.1	0.02	-1.6
A_55_P2008197	NM_207707	Esr2	1	-1.2	0.97	-1.1	0.02	-1.6
A_52_P447424			1	-1.1	0.93	-1.2	0.02	-1.6
A_66_P131433			1	-1	0.9	-1.2	0.02	-1.6
A_52_P543040	NM_028276	Utp14a	1	-1.2	0.52	-1.3	0.01	-1.6
A_55_P2028576	XM_001478577	Gm7057	1	-1	0.98	-1.1	0.01	-1.6
A_55_P2086885	NM_001033219	Slc45a4	1	-1.2	0.47	-1.3	0.01	-1.6
A_55_P2180445	XM_001474578	Gm2742	1	-1	1	-1	0.01	-1.6
A_55_P1987409	NM_173444	Nbeal1	1	1	0.98	-1.1	0.01	-1.6
A_52_P96782	NM_028459	Wasl	1	-1.1	0.85	-1.2	0.01	-1.6
A_55_P2137286	XM_001479609	Gm4408	1	1.1	0.99	-1.1	0.01	-1.6
A_55_P2121760	XM_001476650	Gm9147	1	-1	1	-1	0.01	-1.6
A_55_P2072980	NM_001076554	Spna2	1	-1	0.41	-1.4	0.01	-1.6
A_55_P2040775	XM_900336	Gm6837	1	-1.1	0.98	-1.1	0.01	-1.6
A_52_P134023	NM_153100	Rtp3	1	-1	0.78	-1.3	0.01	-1.6
A_30_P01027700			1	-1.1	0.8	-1.2	0	-1.6
A_55_P2072403	NM_022724	Suv39h2	0.85	-1.2	0.52	-1.2	0	-1.6
A_55_P2068496	XM_001474158	Gm11412	1	-1	0.93	-1.1	0	-1.6
A_55_P2152188	XM_001473965	Gm2556	1	-1.1	0.43	-1.3	0	-1.6
A_51_P192089	NM_028228	2610028A01Rik	0.94	-1.2	0.93	-1.1	0	-1.6
A_52_P163515			1	-1	0.39	-1.3	0	-1.6
A_30_P01022868			1	-1.1	0.84	-1.2	0	-1.6
A_55_P2408415	NR_001461	Kcnq1ot1	1	-1.1	0.21	-1.4	0	-1.6
A_55_P1964332	XM_001479828	LOC100048239	1	-1.1	0.9	-1.2	0	-1.6
A_55_P1966383	XM_001475163	Gm2936	1	-1.1	0.97	-1.1	0	-1.6
A_55_P2144364	XM_001479624	Gm4320	1	-1.2	0.67	-1.3	0	-1.6
A_55_P2003199	AK141316		1	-1.1	0.95	-1.1	0	-1.6
A_55_P2462940	NM_001081008	Taf1	1	-1.2	0.74	-1.2	0	-1.6
A_52_P161297	NM_011542	Tcea3	1	-1.3	0.18	-1.5	0	-1.6
A_30_P01029602			1	-1.1	0.88	-1.2	0	-1.6
A_30_P01027795			1	-1.2	0.07	-1.4	0	-1.6
A_55_P2165539	NM_009637	Aebp2	1	-1.1	0.56	-1.2	0	-1.6
A_30_P01031578			1	-1.2	0.87	-1.2	0	-1.6
A_51_P474053	NM_001166206	Erv3	0.89	-1.2	0	-1.4	0	-1.6
A_30_P01030106			1	-1.2	0.29	-1.3	0	-1.6
A_51_P439085	NM_023516	2310016C08Rik	0.18	-1.4	0.02	-1.5	0	-1.7
A_55_P2421835	AK015812	4930517G19Rik	1	-1.2	0.02	-1.5	0	-1.7
A_55_P2058783	NM_023516	2310016C08Rik	0.38	-1.4	0	-1.5	0	-1.7
A_55_P2171623	NM_001077363	Ptbp1	1	-1.1	0.03	-1.8	0	-1.7
A_55_P2108486	XM_903408	LOC630284	1	-1.1	0.95	-1.2	0.04	-1.7
A_66_P112862	NR_033146	LOC100316870	1	-1.2	0.88	-1.3	0.04	-1.7
A_55_P2209053	AK013461	2900001G08Rik	1	-1.2	0.88	-1.3	0.04	-1.7
A_55_P2183498	NM_025668	Spcs2	1	-1.2	0.86	-1.3	0.04	-1.7
A_55_P1968763	NM_199022	Shc4	1	1	0.99	-1.1	0.03	-1.7
A_55_P2102738	XM_001478003	Gm3781	1	1.2	0.99	1.1	0.03	-1.7
A_30_P01026040			1	-1.1	0.97	-1.2	0.03	-1.7
A_30_P01032670			1	-1.1	0.91	-1.2	0.03	-1.7

A_30_P01028808			1	-1.2	0.8	-1.3	0.03	-1.7
A_66_P140533	XM_001478088	Gm3813	1	-1.1	0.94	-1.2	0.02	-1.7
A_55_P1958245	NM_011552	Tcof1	1	-1.3	0.37	-1.5	0.02	-1.7
A_55_P2001238	NM_026313	Luc7l3	1	-1.2	0.91	-1.2	0.02	-1.7
A_52_P45738	NM_029979	Trim35	1	-1.2	0.41	-1.5	0.02	-1.7
A_66_P112065	AK142268	Sertad2	1	-1.1	0.94	-1.2	0.02	-1.7
A_55_P2077866	NM_010918	Nktr	1	-1.1	0.85	-1.2	0.02	-1.7
A_55_P2046728	XM_981891	Gm7792	1	-1.2	0.86	-1.2	0.02	-1.7
A_30_P01020465			1	-1.1	0.92	-1.2	0.02	-1.7
A_55_P2162782	NM_009643	Ahnak	1	-1.1	0.99	-1.1	0.02	-1.7
A_55_P2105517	XM_001473956		1	-1.2	0.55	-1.4	0.02	-1.7
A_55_P2027337	XM_001473753	LOC100045145	1	-1.2	0.91	-1.2	0.02	-1.7
A_51_P116813	NM_007809	Cyp17a1	1	-1.1	0.88	-1.2	0.02	-1.7
A_30_P01026338			1	-1.1	0.89	-1.2	0.02	-1.7
A_55_P1988260	XM_001479382	LOC100048847	1	-1.1	0.84	-1.3	0.01	-1.7
A_52_P565940	NM_008739	Nsd1	1	-1.1	0.78	-1.3	0.01	-1.7
A_55_P2072041	XM_001472087	LOC100038980	1	-1.1	0.96	-1.1	0.01	-1.7
A_55_P2021923	AB241120		1	-1.2	0.84	-1.2	0.01	-1.7
A_52_P508991	NM_010231	Fmo1	1	-1.2	0.91	-1.2	0.01	-1.7
A_30_P01020298			1	-1.2	0.68	-1.3	0.01	-1.7
A_52_P233441	NM_008090	Gata2	1	-1.2	0.45	-1.4	0.01	-1.7
A_30_P01020570			1	-1.1	0.86	-1.2	0.01	-1.7
A_55_P2079116	NR_002142	Rpph1	1	-1.2	0.16	-1.5	0	-1.7
A_55_P2048660	XM_908118	LOC633654	1	-1.2	0.83	-1.2	0	-1.7
A_55_P2081388	NM_008575	Mdm4	1	-1.1	0.92	-1.1	0	-1.7
A_52_P79187	NM_001025572	Ankrd12	1	-1.1	0.85	-1.2	0	-1.7
A_55_P2158522	NM_183355	Pbx1	1	-1.2	0.62	-1.3	0	-1.7
A_55_P2126662	NM_007616	Cav1	1	-1.1	0.34	-1.3	0	-1.7
A_55_P2170834	CA461216		1	-1.2	0.72	-1.3	0	-1.7
A_55_P1977875	NM_176849	Arglu1	1	-1.1	0.15	-1.3	0	-1.7
A_66_P139618	NM_001082545	Stfa2	1	-1.1	0.83	-1.2	0	-1.7
A_55_P2021476	XM_001474821		1	-1.1	0.97	-1.1	0	-1.7
A_55_P2212027	NM_011682	Utrn	1	-1.2	0.73	-1.2	0	-1.7
A_55_P2020361	NM_197945	RP23-100C5.8	1	-1	0.98	-1.1	0	-1.7
A_55_P2148402	NM_009946	Cplx2	1	-1.1	0.99	-1.1	0	-1.7
A_55_P2087567	NM_146539	Olfr373	1	-1.1	0.66	-1.3	0	-1.7
A_55_P2072315	AK133410		1	-1.2	0.7	-1.3	0	-1.7
A_55_P2104917	NM_016902	Nphp1	0.88	-1.3	0.37	-1.4	0	-1.7
A_55_P2083149	XM_001476298	LOC100046485	1	-1.1	0.84	-1.2	0	-1.7
A_55_P2050169	NM_134244	V1rh21	1	-1.2	0.1	-1.4	0	-1.7
A_30_P01022768			1	-1.1	0.64	-1.3	0	-1.7
A_55_P2159850	XM_001479402	Gm4191	1	-1.1	0.96	-1.1	0	-1.7
A_55_P1965030	NM_001003915	Slc5a12	0.36	-1.5	0.15	-1.4	0	-1.7
A_55_P1989865	NM_146153	Thrap3	0.92	-1.3	0.27	-1.4	0	-1.7
A_66_P106774	NM_029990	Lhfpl3	0	-1.6	0	-1.7	0	-1.8
A_52_P650387	NM_001045530	Ccnjl	0.33	-1.9	0.03	-2.2	0.05	-1.8
A_55_P2430472	NM_011805	Dido1	1	-1.2	0.85	-1.3	0.04	-1.8
A_55_P2008437	NM_009877	Cdkn2a	1	-1.3	0.51	-1.5	0.03	-1.8
A_51_P150710	NM_152839	Igj	1	-1.4	0.48	-1.5	0.03	-1.8
A_55_P2179246	XM_001472564	LOC100044553	1	-1.2	0.99	-1.1	0.03	-1.8
A_55_P1962603	NM_009946	Cplx2	1	-1	0.99	1.1	0.03	-1.8
A_52_P140881	NM_177615	Slc26a10	0.59	-1.6	0.07	-2	0.03	-1.8
A_55_P1993858	NR_001460	Rmrp	1	-1.1	0.31	-1.6	0.03	-1.8
A_66_P123735	AK143547		1	-1.2	0.88	-1.2	0.03	-1.8
A_55_P2127174	NM_170759	Zfp628	1	-1.1	0.89	-1.3	0.02	-1.8

A_55_P2018307	XM_001005025	LOC677576	1	-1.2	0.85	-1.3	0.02	-1.8
A_51_P267544	NM_013522	Frg1	1	-1.2	0.73	-1.4	0.02	-1.8
A_51_P179258	NM_001161665	Kif26b	1	-1.3	0.57	-1.4	0.02	-1.8
A_55_P2033215	NM_013877	Cabp5	1	-1	0.98	-1.1	0.02	-1.8
A_55_P1962602	XM_001474429	Gm2690	1	1	0.98	1.1	0.02	-1.8
A_51_P124345	NM_153166	Cpne5	1	-1.2	0.86	-1.2	0.01	-1.8
A_55_P2146185	NM_011302	Rs1	1	-1	0.97	-1.1	0.01	-1.8
A_30_P01025354			1	-1.1	0.87	-1.2	0.01	-1.8
A_55_P2148400	XM_001472970	Gm2264	1	1	1	1	0.01	-1.8
A_51_P128463	NM_001099296	Grrp1	1	-1.1	0.51	-1.4	0.01	-1.8
A_55_P1994173	XM_001474573	EG622110	1	-1.2	0.97	-1.1	0.01	-1.8
A_51_P153423	NM_001081416	Fndc1	0.58	-1.5	0.49	-1.4	0.01	-1.8
A_55_P2126557	XM_619973	Gm5858	0.83	-1.4	0.21	-1.6	0.01	-1.8
A_55_P2133624	XM_001475193	Gm2891	1	-1.2	0.82	-1.3	0.01	-1.8
A_30_P01019885			0.91	-1.2	0.15	-1.4	0	-1.8
A_55_P1992571	XM_001475200	Gm2952	1	-1.1	0.88	-1.2	0	-1.8
A_55_P2030160	NM_001142920	Tcf7l2	1	-1.2	0.05	-1.4	0	-1.8
A_52_P163849	NM_001039511	Ivns1abp	1	-1.2	0.79	-1.3	0	-1.8
A_55_P2039196	NM_020014	Gfra4	1	-1.1	0.59	-1.3	0	-1.8
A_55_P1999818	XM_001472026	Gm2006	1	-1.1	0.81	-1.3	0	-1.8
A_55_P2004168	XM_001476023	LOC100046325	1	-1.1	0.99	-1.1	0	-1.8
A_55_P2403769	BB498095	AI481121	0.48	-1.5	0.37	-1.4	0	-1.8
A_52_P550049	NM_001110832	Nfya	0.88	-1.3	0.13	-1.4	0	-1.8
A_30_P01028277			1	-1.1	0.58	-1.2	0	-1.8
A_55_P2015782	NM_029815	Bcas1	1	-1.1	0	-1.4	0	-1.8
A_55_P2047620	XM_001475740	Gm3124	1	-1.1	0.97	-1.1	0	-1.8
A_55_P2042184	XM_001476516	Gm3181	1	-1.1	0.85	-1.2	0	-1.8
A_55_P2183668			1	-1.2	0.62	-1.4	0	-1.8
A_51_P310164	NM_001144992	2810459M11Rik	1	-1.2	0	-1.4	0	-1.8
A_30_P01031586			1	-1.1	0.49	-1.3	0	-1.8
A_30_P01022751			1	-1.2	0.37	-1.3	0	-1.8
A_30_P01031694			0.2	-1.7	0	-1.9	0.01	-1.9
A_30_P01020334			0.11	-2	0.04	-2	0.01	-1.9
A_55_P2064025	XM_001474990		1	-1.2	0.85	-1.3	0.03	-1.9
A_30_P01029062			1	-1.2	0.91	-1.3	0.03	-1.9
A_30_P01025909			1	-1.2	0.93	-1.3	0.03	-1.9
A_51_P464822	NM_015787	Hist1h1e	1	1	0.73	-1.4	0.03	-1.9
A_55_P2017769	NM_001033769	B020031M17Rik	1	-1.3	0.97	-1.2	0.03	-1.9
A_55_P2138739	XM_001473119	LOC100039570	1	-1.2	0.99	-1.1	0.02	-1.9
A_30_P01026192			0.74	-1.5	0.61	-1.4	0.01	-1.9
A_55_P1967002	XM_001473953	Gm9441	1	-1.1	0.98	-1.1	0.01	-1.9
A_55_P2165790	NM_178706	Siglech	1	-1.3	0.7	-1.3	0.01	-1.9
A_51_P509679	XM_001474025		0.8	-1.5	0.07	-1.8	0.01	-1.9
A_30_P01030968			1	-1.1	0.68	-1.3	0.01	-1.9
A_30_P01025536			1	-1.2	0.58	-1.4	0	-1.9
A_55_P2151209	NM_130873	Krtap16-4	1	-1.2	0.72	-1.3	0	-1.9
A_55_P2009042	XM_001481023	Gm4635	1	-1.2	0.91	-1.3	0	-1.9
A_55_P1959973	NM_175539	Dcaf12l2	1	-1.1	0.97	-1.1	0	-1.9
A_55_P2025820	XM_001480287		1	-1	0.67	-1.4	0	-1.9
A_55_P2183672	NM_018858	Pebp1	1	-1.2	0.78	-1.3	0	-1.9
A_55_P2014978	NM_007889	Dvl3	1	-1.2	0.6	-1.3	0	-1.9
A_66_P125110	XM_001475977	Gm3006	1	-1.1	0.89	-1.2	0	-1.9
A_55_P2076805	AK129022		1	-1.1	0.85	-1.2	0	-1.9
A_55_P2172934	NM_029879	Rgs7bp	1	-1.1	0.98	-1.1	0	-1.9
A_52_P544476	NR_001579	Terc	0.81	-1.2	0	-1.6	0	-2

A_55_P1960148	XM_001472091		0.83	-1.5	0.04	-1.8	0	-2
A_55_P1968085			1	-1.2	0.99	-1.1	0.04	-2
A_30_P01032760			1	-1.2	0.86	-1.4	0.03	-2
A_55_P2020035	XM_001473697	LOC100045113	1	-1.2	0.99	-1.1	0.03	-2
A_55_P1981829	NM_001004193	Rhox8	1	-1.2	0.98	-1.1	0.02	-2
A_55_P2183518	XM_001473350	LOC100044930	1	-1.4	0.94	-1.2	0.02	-2
A_55_P2073935	NM_013790	Abcc5	1	-1.3	0.68	-1.4	0.02	-2
A_65_P15809	NR_003967	Gm4759	1	-1.2	0.92	-1.3	0.02	-2
A_55_P2174743	NM_018747	Akap7	1	-1.3	0.99	-1.1	0.01	-2
A_30_P01018086			1	-1.3	0.85	-1.3	0.01	-2
A_55_P2044380	XM_001476392	LOC100046544	1	-1.2	0.86	-1.3	0.01	-2
A_30_P01022120			1	-1.3	0.36	-1.5	0.01	-2
A_66_P113505	XM_001475977	Gm3006	1	-1.2	0.88	-1.3	0.01	-2
A_52_P467690	NM_175836	Spnb2	1	-1.2	0.58	-1.4	0	-2
A_55_P2454099	NM_023799	Mgea5	1	-1.3	0.32	-1.5	0	-2
A_55_P2092831	XM_001472097	Gm2008	1	-1.2	0.97	-1.1	0	-2
A_30_P01031356			0.68	-1.2	0	-1.4	0	-2
A_30_P01026230			1	-1.2	0.82	-1.3	0	-2
A_55_P1977812	NM_018729	Cd244	0.97	-1.2	0.1	-1.4	0	-2
A_52_P311031	AK016943		1	-1.2	0.78	-1.3	0	-2
A_30_P01020476			1	-1.2	0.83	-1.3	0	-2
A_55_P2115906	NM_181680	Dsg1c	0.85	-1.2	0.04	-1.4	0	-2
A_55_P2249379	AK016549	ENSMUSG00000038461	0.95	-1.2	0.02	-1.4	0	-2
A_55_P2025483	NM_178916	Rfesd	1	-1.1	0.48	-1.3	0	-2
A_30_P01023509			0.93	-1.2	0	-1.4	0	-2
A_55_P2010586	AK132285	Gm6252	1	-1.1	0.67	-1.3	0	-2
A_55_P2408588	NM_007489	Arntl	0	-2.4	0	-2.9	0.03	-2.1
A_55_P1975412	NM_146067	Cpped1	0.98	-1.2	0	-1.5	0	-2.1
A_55_P2022629	NM_181859	Oxct2b	0.95	-1.2	0.02	-1.6	0	-2.1
A_55_P1991783	XM_001477578	Gm7149	1	-1.3	0.97	-1.2	0.02	-2.1
A_55_P2028847	XM_001476302	Gm9468	1	-1.2	0.96	-1.2	0.02	-2.1
A_55_P2021398	XM_001473399	Gm2393	1	-1.3	0.97	-1.2	0.01	-2.1
A_55_P2116149			1	-1.2	0.96	-1.2	0.01	-2.1
A_55_P1961760	XM_001476058	Gm3237	1	-1.3	0.95	-1.2	0.01	-2.1
A_55_P2114697	NM_008103	Gcm1	1	-1.2	0.95	-1.2	0.01	-2.1
A_55_P2077497	XM_001476406	Gm6940	1	-1.3	0.97	-1.2	0.01	-2.1
A_55_P2035038	XM_001473590	Gm2437	1	-1.2	0.99	-1.1	0.01	-2.1
A_55_P2070766	XM_001476070	Gm3241	1	-1.2	0.98	-1.1	0.01	-2.1
A_30_P01024297			1	-1.2	0.72	-1.4	0	-2.1
A_55_P2019833	XM_001477941	Gm3790	1	-1.3	0.97	-1.2	0	-2.1
A_30_P01022343			1	-1.2	0.53	-1.4	0	-2.1
A_55_P2086954	XM_001479991	Gm4372	1	-1.3	0.94	-1.2	0	-2.1
A_55_P2014326	XM_001478106	LOC100047416	1	-1.2	0.88	-1.2	0	-2.1
A_55_P1982227	XM_001480822	LOC100048656	1	-1.2	0.97	-1.2	0	-2.1
A_55_P2001494	NM_013598	Kitl	1	-1.1	0.87	-1.3	0	-2.1
A_55_P2075731	XM_001475177	Gm2943	1	-1.3	0.98	-1.2	0	-2.1
A_55_P2105321	NM_023190	Acin1	1	-1.2	0.67	-1.4	0	-2.1
A_52_P221776	NM_010616	Kif12	0.98	-1.2	0.02	-1.5	0	-2.2
A_30_P01022623			0.69	-1.3	0.02	-1.5	0	-2.2
A_55_P2002309	NM_198191	Pip5kl1	0.73	-1.2	0	-1.5	0	-2.2
A_55_P1998937	NM_177635	H2-M11	1	-1.2	0	-1.5	0	-2.2
A_55_P2071526	NM_130887	Papln	0.64	-1.2	0	-1.6	0	-2.2
A_55_P1968365	XM_001472316	LOC100044456	0.85	-1.2	0	-1.6	0	-2.2
A_55_P2029902	NM_181584	Gab3	1	-1.3	0.98	-1.2	0.04	-2.2
A_55_P1960738	XM_001472780	Gm4470	1	-1.1	0.88	-1.4	0.04	-2.2

A_55_P2041457	XM_001475948	Gm3195	1	-1.3	0.97	-1.2	0.03	-2.2
A_55_P2148935	XM_001474432	LOC100040286	1	-1.3	0.97	-1.2	0.03	-2.2
A_55_P2096127	XM_001473875	Gm2527	1	-1.3	0.97	-1.2	0.03	-2.2
A_55_P1962699	XM_001477233	LOC100047021	1	-1.3	0.97	-1.2	0.02	-2.2
A_55_P2068247	XM_001473755	Gm2488	1	-1.3	0.98	-1.2	0.02	-2.2
A_55_P2009449	NM_175498	Pnma2	1	-1.3	0.99	-1.1	0.02	-2.2
A_55_P2139430	XM_001479756	LOC100048207	1	-1.3	0.97	-1.2	0.02	-2.2
A_55_P1967591	XM_001472709	LOC100044615	1	-1.3	0.98	-1.2	0.02	-2.2
A_55_P2143516	XM_001473058	Gm2291	1	-1.3	0.95	-1.2	0.02	-2.2
A_55_P1960999	NM_011082	Pigr	1	-1.3	0.97	-1.2	0.01	-2.2
A_55_P2068248	XM_001473755	Gm2488	1	-1.3	0.97	-1.2	0.01	-2.2
A_55_P1960479	XM_001475709	Gm3114	1	-1.2	0.97	-1.2	0.01	-2.2
A_51_P262701	NM_019775	Cpb2	1	-1.4	0.51	-1.5	0.01	-2.2
A_30_P01032942			1	-1.2	0.75	-1.3	0	-2.2
A_55_P2171788	XM_001474084	Gm2598	1	-1.3	0.97	-1.2	0	-2.2
A_55_P1979067	XM_001481217	Gm4720	1	-1.3	0.58	-1.5	0	-2.2
A_51_P210143	NM_001005510	Syne2	0.92	-1.3	0.18	-1.5	0	-2.2
A_55_P2153620	NM_001039959	Ahnak	1	-1.3	0.75	-1.4	0	-2.2
A_55_P2165249	NM_130887	Papln	1	-1.2	0.09	-1.5	0	-2.2
A_55_P2175469	NM_001081345	Chd2	1	-1.3	0.64	-1.5	0	-2.2
A_55_P2082841	XM_001474255	Gm2658	1	-1.3	0.98	-1.2	0	-2.2
A_30_P01033617			0.42	-1.4	0.03	-1.5	0	-2.3
A_66_P113141	AK042438		0.97	-1.2	0	-1.6	0	-2.3
A_55_P2177347	AK132138		0.81	-1.3	0	-1.6	0	-2.3
A_55_P1981830	XM_001475952	LOC100041203	1	-1.3	0.98	-1.2	0.04	-2.3
A_55_P2165554			1	-1.5	0.23	-2.1	0.03	-2.3
A_55_P1974780	XM_001476301	Gm3306	1	-1.3	0.97	-1.2	0.01	-2.3
A_55_P2077501	XM_901095	Gm6940	1	-1.3	0.97	-1.2	0.01	-2.3
A_52_P461343	NM_023478	Upk3a	0.5	-1.8	0.07	-2.1	0.01	-2.3
A_55_P2120141	XM_001480410	Gm4522	1	-1.3	0.98	-1.2	0.01	-2.3
A_30_P01032775			0.97	-1.2	0.1	-1.4	0	-2.3
A_66_P103231	XM_001474378	Gm2682	1	-1.2	0.06	-1.5	0	-2.3
A_66_P126323	AK020309		1	-1.2	0.02	-1.6	0	-2.4
A_55_P2113310			1	-1.2	0	-1.6	0	-2.4
A_30_P01022027			0.9	-1.2	0	-1.6	0	-2.4
A_55_P2465382	AK172117		1	-1.6	0.1	-2.5	0.04	-2.4
A_55_P2063594	NR_002889	Gm5801	0.87	-1.8	0.07	-2.4	0.03	-2.4
A_55_P2136752	NM_029972	Ermn	1	-1.3	0.97	-1.2	0.01	-2.4
A_55_P2064171			1	-1.3	0.98	-1.2	0.01	-2.4
A_55_P2063505	XM_001479508	Gm4235	1	-1.3	0.98	-1.2	0.01	-2.4
A_51_P331328	NM_026730	Gpihbp1	1	-1.1	0.75	-1.4	0	-2.5
A_30_P01019238			1	-1.2	0.21	-1.5	0	-2.5
A_55_P2020726	XM_001472218	Gm16505	1	-1.2	0	-1.8	0	-2.7
A_55_P2141479	NR_004414	Rnu2	1	-1.3	0.45	-1.7	0	-2.7
A_51_P356055	NM_175012	Grp	0	-2	0	-2.2	0	-2.8
A_55_P2115567	NM_174870	Slc26a1	1	-1.2	0.04	-1.7	0	-2.8
A_30_P01027915			0.61	-1.4	0	-1.8	0	-2.8
A_30_P01030041			0.07	-2.5	0	-2.7	0	-2.8
A_30_P01032068			0.74	-1.5	0.02	-2.1	0	-2.9
A_51_P175424	NM_011797	Car14	0	-2	0	-2.8	0	-3.3
A_55_P2145307	NM_028020	Cpsf3l	0.88	-2.4	0.11	-3.6	0.03	-3.5
A_51_P501844	NM_175475	Cyp26b1	0.61	-2.6	0.18	-3.2	0	-4.3
