# Supplementary figures and images for: A framework for the use of single-chemical transcriptomics data in predicting the hazards associated with complex mixtures of polycyclic aromatic hydrocarbons
Source: Arch Toxicol. 2016 Nov 17;91(7):2599–616. doi: 10.1007/s00204-016-1891-8 (PMC5489644; doi:10.1007/s00204-016-1891-8)

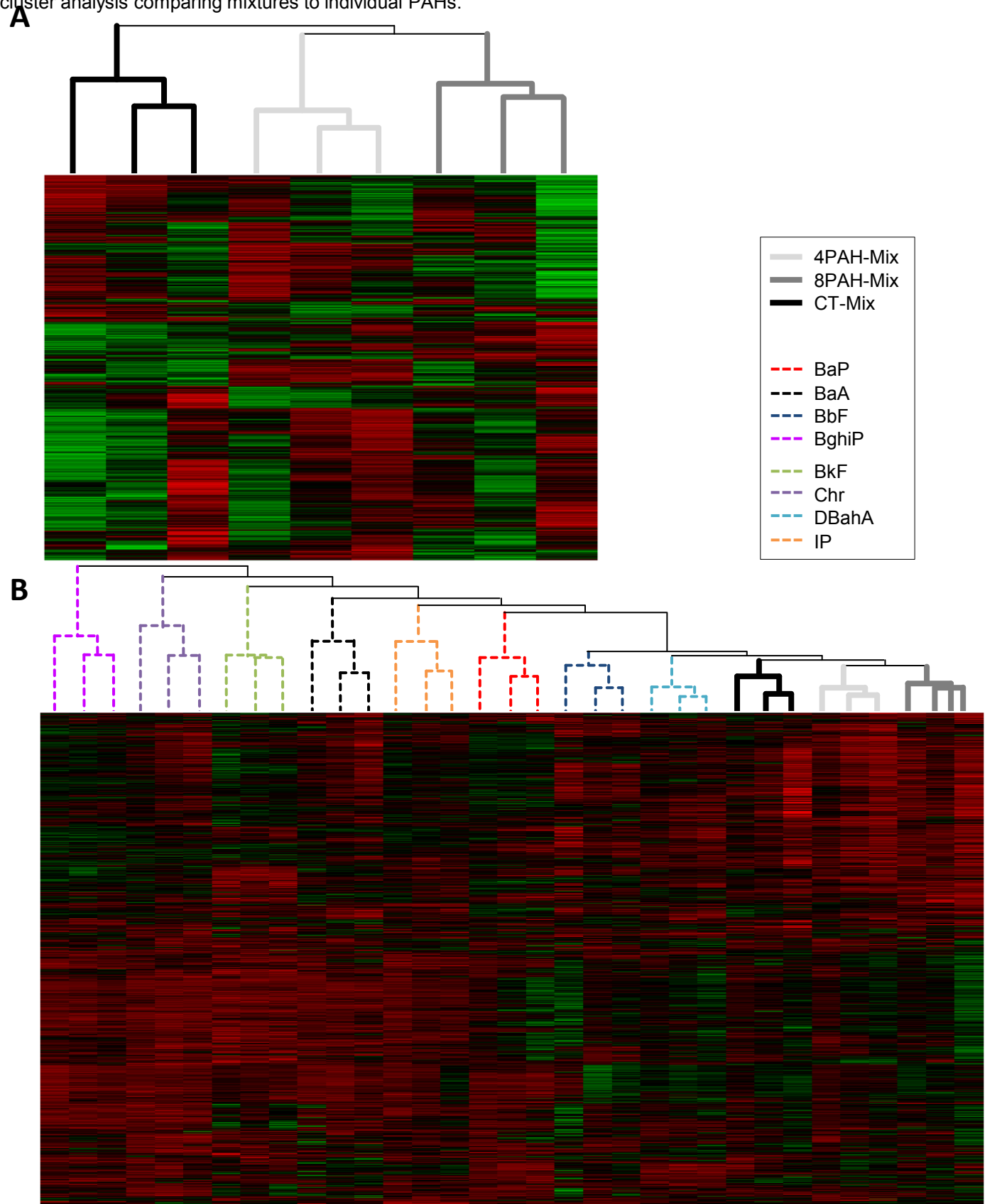

Supplement: Supplementary file 3 — Hierarchical cluster analyses (PDF 330 kb) [file 204_2016_1891_MOESM3_ESM.pdf]
