# Supplementary material for: A framework for the use of single-chemical transcriptomics data in predicting the hazards associated with complex mixtures of polycyclic aromatic hydrocarbons
Source: Arch Toxicol. 2016 Nov 17;91(7):2599–616. doi: 10.1007/s00204-016-1891-8 (PMC5489644; doi:10.1007/s00204-016-1891-8)
Supplement: Supplementary file 4 — List of median and mean gene expression changes for each pathway in lungs from mice exposed to the 4PAH-Mix, 8PAH-Mix, CT-Mix, and the individual PAHs (PDF 274 kb) [file 204_2016_1891_MOESM4_ESM.pdf]

Article Title: A framework for the Use of Single Chemical Transcriptomics Data in Predicting the Hazards Associated with Complex Mixtures of Polycyclic Aromatic Hydrocarbons
Journal: Archives of Toxicology
Authors: Sarah Labib, Andrew Williams, Byron Kuo, Carole L Yauk, Paul A White, Sabina Halappanavar*
Corresponding Author: *Sabina Halappanavar, Health Canada, sabina.halappanavar@hc-sc.gc.ca

Online Resource 4. Median and mean fold changes for AHR signaling, apoptosis, cell cycle, circadian rhythm, P53 signaling, and xenobiotic metabolism signaling pathways. The max gene column shows the fold change for the maximally perturbed gene associated with the pathway.

Treatment	Dose group	Dose (mg/kg-day)	AHR Signaling			Apoptosis			Cell cycle			Circadian rhythm			P53 signaling			Xenobiotic metabolism		
			median	mean	max gene	median	mean	max gene	median	mean	max gene	median	mean	max gene	median	mean	max gene	median	mean	max gene
BaP	D1	25	1.38	1.59	2.58	1.31	1.39	2.58	1.40	1.51	2.58	1.70	1.70	1.74	1.42	1.56	2.58	1.29	1.38	1.41
	D2	50	1.40	2.54	6.10	1.51	1.80	6.10	1.51	2.10	6.10	1.61	1.81	1.87	1.70	2.31	6.10	1.38	1.29	1.32
	D3	75	1.56	4.34	10.00	1.70	2.22	10.00	1.75	2.81	10.00	1.81	1.61	1.64	2.04	3.11	10.00	1.65	1.65	1.73
BaA	D1	20	1.50	1.55	1.81	1.18	1.25	2.28	1.19	1.23	2.28	1.13	1.25	1.64	1.22	1.34	1.81	1.22	1.40	2.28
	D2	40	1.58	1.56	1.65	1.41	1.48	2.94	1.42	1.44	2.06	1.57	1.77	2.78	1.61	1.56	2.14	1.46	1.48	1.81
	D3	80	1.61	1.78	2.30	1.57	1.69	3.39	1.62	1.67	2.33	1.87	2.13	3.54	1.62	1.82	3.07	1.48	1.55	2.20
BbF	D1	25	1.48	1.68	3.13	1.21	1.29	3.13	1.27	1.37	3.13	1.48	1.93	4.42	1.22	1.48	3.13	1.29	1.44	4.40
	D2	50	1.63	1.87	4.14	1.33	1.47	4.14	1.34	1.50	4.14	1.54	2.32	6.00	1.46	1.72	4.14	1.36	1.51	4.57
	D3	100	2.08	4.79	19.06	1.62	2.09	17.52	1.59	2.03	19.06	1.92	2.89	7.68	1.70	2.33	7.18	1.67	3.36	19.06
CHR	D1	17.5	1.16	1.49	2.18	1.12	1.20	2.28	1.13	1.19	2.18	1.17	1.16	1.18	1.07	1.33	2.28	1.12	1.23	2.18
	D2	50	1.89	1.89	1.89	1.17	1.32	2.92	1.19	1.24	1.89	1.64	1.62	2.74	1.47	1.67	2.92	1.11	1.24	1.89
	D3	150	1.48	1.64	1.98	1.68	2.13	9.66	1.59	1.74	3.00	2.04	2.07	1.91	2.74	2.49	3.83	1.60	1.72	2.31
BghiP	D1	6.25	1.23	1.19	1.26	1.22	1.25	1.77	1.17	1.16	1.34	1.32	1.30	1.61	1.04	1.04	1.06	1.11	1.11	1.11
	D2	12.5	1.31	1.26	1.65	1.38	1.40	2.02	1.37	1.35	1.78	2.14	2.21	2.88	1.03	1.03	1.04	1.48	1.48	1.48
	D3	25	1.48	1.54	1.70	1.65	1.62	2.09	1.59	1.59	1.71	2.21	2.11	2.65	1.65	1.65	1.70	1.50	1.50	1.50
BkF	D1	25	1.29	3.68	16.96	1.23	1.45	10.08	1.16	1.77	16.96	1.16	1.46	2.17	1.20	1.25	1.51	1.34	3.13	16.96
	D2	50	1.77	12.91	80.15	1.46	2.20	41.55	1.31	4.12	80.15	1.34	1.58	2.44	1.41	1.55	2.12	1.49	9.48	80.15
	D3	100	1.76	19.42	117.29	1.56	2.62	65.63	1.59	5.63	117.29	1.64	2.12	3.38	1.65	1.62	1.95	1.67	14.18	117.29
DBahA	D1	6.25	1.60	2.41	4.40	1.36	1.60	6.13	1.52	1.68	4.40	1.38	1.70	3.27	1.53	1.83	4.40	1.31	1.77	4.29
	D2	12.5	1.95	2.78	6.34	1.55	1.91	9.13	1.62	1.84	6.34	1.52	2.28	5.15	1.71	2.21	6.34	1.46	1.81	5.07
	D3	25	1.94	2.96	7.09	1.66	2.08	11.56	1.66	1.92	7.09	1.58	2.33	5.13	1.82	2.43	7.09	1.53	1.92	5.27
IP	D1	12.5	1.35	1.46	2.05	1.31	1.41	2.61	1.15	1.16	1.53	1.56	1.46	1.74	1.16	1.24	1.63	1.32	1.45	2.05
	D2	25	1.98	2.56	5.52	1.51	1.73	3.01	1.24	1.42	5.52	2.61	2.62	3.75	1.50	1.65	3.01	2.06	2.44	5.52
	D3	50	2.52	5.20	17.83	1.71	2.23	12.54	1.65	2.22	17.83	2.67	3.20	5.21	1.82	1.90	2.23	2.11	5.59	17.83
4PAH-Mix	D1	12.5	1.45	1.71	3.39	1.32	1.46	4.10	1.32	1.39	3.39	1.71	2.01	3.69	1.34	1.52	3.39	1.25	1.59	3.97
	D2	25	1.55	2.26	5.94	1.54	1.77	6.91	1.47	1.65	5.94	1.83	2.39	4.92	1.49	1.92	5.94	1.50	1.89	4.76
	D3	50	2.02	2.82	7.55	1.66	2.03	7.55	1.57	1.85	7.55	1.89	2.57	5.44	1.62	2.23	7.55	2.01	2.33	6.25
8PAH-Mix	D1	15	1.31	1.54	3.24	1.26	1.39	3.24	1.35	1.40	2.53	1.30	1.59	2.79	1.31	1.38	2.33	1.30	1.71	3.95
	D2	30	1.40	2.42	7.99	1.42	1.60	5.75	1.47	1.68	7.99	1.64	2.07	4.32	1.42	1.56	2.40	1.36	2.52	7.99
	D3	60.1	1.78	2.81	9.27	1.69	1.97	9.48	1.57	1.83	9.27	1.70	2.14	3.52	1.66	1.84	2.92	1.79	2.99	9.27
CT-Mix	D1	1.3	1.78	1.74	2.97	1.28	1.43	4.94	1.26	1.34	2.97	1.82	2.45	4.35	1.34	1.45	2.31	1.48	1.65	2.97
	D2	2.5	2.03	2.55	7.08	1.46	1.66	6.52	1.40	1.55	7.08	2.02	2.48	5.59	1.51	1.75	3.19	1.68	2.43	7.08
	D3	5.1	2.11	3.43	9.15	1.62	2.02	9.10	1.62	1.81	9.15	2.07	3.00	4.54	1.78	2.12	4.91	1.93	3.28	9.15
