# Supplementary material for: A framework for the use of single-chemical transcriptomics data in predicting the hazards associated with complex mixtures of polycyclic aromatic hydrocarbons
Source: Arch Toxicol. 2016 Nov 17;91(7):2599–616. doi: 10.1007/s00204-016-1891-8 (PMC5489644; doi:10.1007/s00204-016-1891-8)

Article Title: A framework for the Use of Single Chemical Transcriptomics Data in Predicting the Hazards Associated with Complex Mixtures of Polycyclic Aromatic Hydrocarbons
Journal: Archives of Toxicology
Authors: Sarah Labib, Andrew Williams, Byron Kuo, Carole L Yauk, Paul A White, Sabina Halappanavar*
Corresponding Author: *Sabina Halappanavar, Health Canada, sabina.halappanavar@hc-sc.gc.ca

Online Resource 5. Impact of no statistical filtering on mixture predictions using mathematical models of additivity

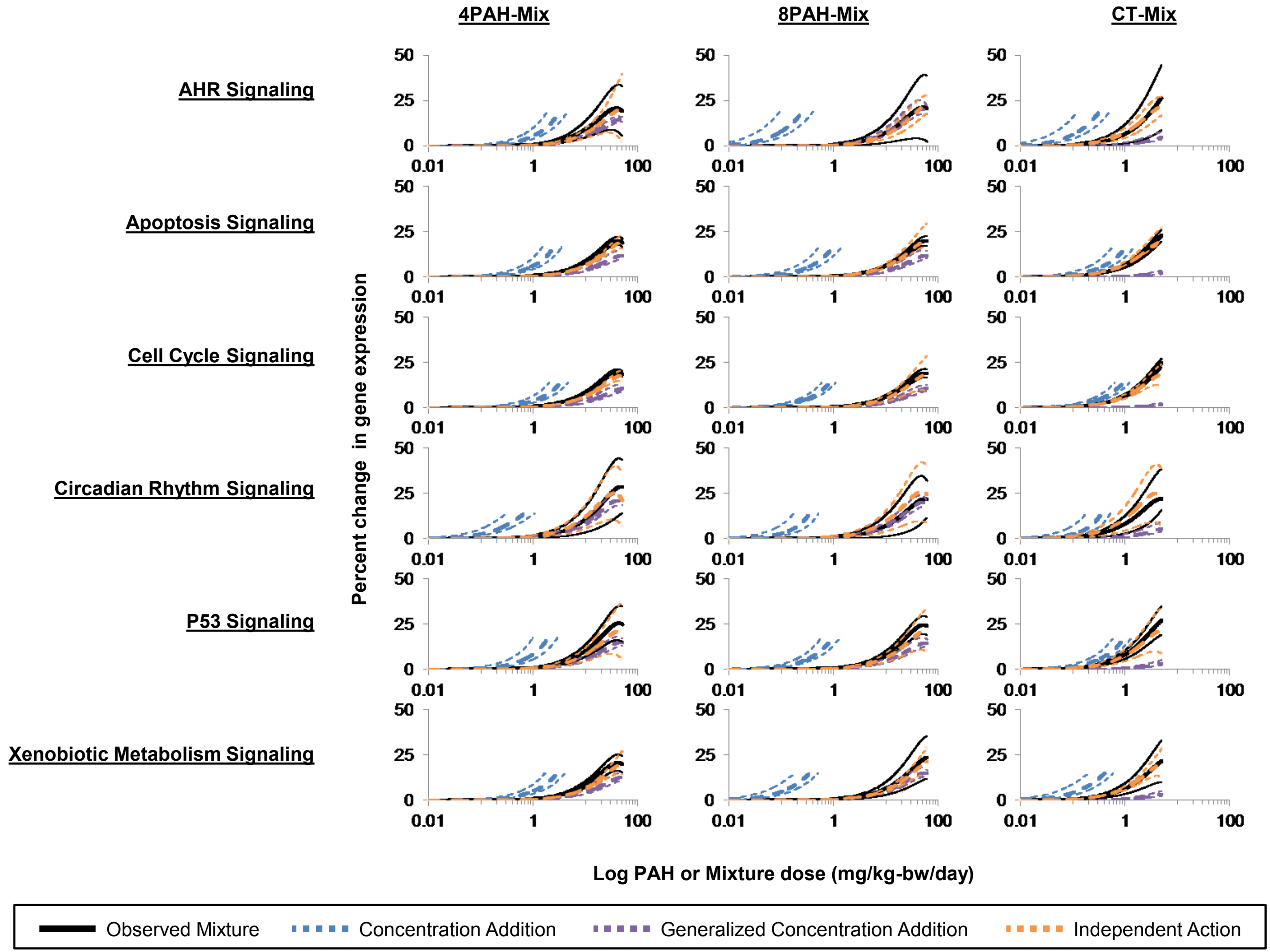

Supplement: Supplementary file 5 — Impact of no statistical filtering on mixture predictions using mathematical models of additivity (PDF 185 kb) [file 204_2016_1891_MOESM5_ESM.pdf]
